# Innovative Molecular Imaging for Clinical Research, Therapeutic Stratification, and Nosography in Neuroscience

**DOI:** 10.3389/fmed.2019.00268

**Published:** 2019-11-27

**Authors:** Marie Beaurain, Anne-Sophie Salabert, Maria Joao Ribeiro, Nicolas Arlicot, Philippe Damier, Florence Le Jeune, Jean-François Demonet, Pierre Payoux

**Affiliations:** ^1^CHU de Toulouse, Toulouse, France; ^2^ToNIC, Toulouse NeuroImaging Center, Inserm U1214, Toulouse, France; ^3^UMR 1253, iBrain, Université de Tours, Inserm, Tours, France; ^4^Inserm CIC 1415, University Hospital, Tours, France; ^5^CHRU Tours, Tours, France; ^6^Inserm U913, Neurology Department, University Hospital, Nantes, France; ^7^Centre Eugène Marquis, Rennes, France; ^8^Leenards Memory Centre, Department of Clinical Neuroscience, Centre Hospitalier Universitaire Vaudois, Lausanne, Switzerland

**Keywords:** molecular imaging, clinical research, neurology, psychiatry, PET, SPECT

## Abstract

Over the past few decades, several radiotracers have been developed for neuroimaging applications, especially in PET. Because of their low steric hindrance, PET radionuclides can be used to label molecules that are small enough to cross the blood brain barrier, without modifying their biological properties. As the use of 11C is limited by its short physical half-life (20 min), there has been an increasing focus on developing tracers labeled with 18F for clinical use. The first such tracers allowed cerebral blood flow and glucose metabolism to be measured, and the development of molecular imaging has since enabled to focus more closely on specific targets such as receptors, neurotransmitter transporters, and other proteins. Hence, PET and SPECT biomarkers have become indispensable for innovative clinical research. Currently, the treatment options for a number of pathologies, notably neurodegenerative diseases, remain only supportive and symptomatic. Treatments that slow down or reverse disease progression are therefore the subject of numerous studies, in which molecular imaging is proving to be a powerful tool. PET and SPECT biomarkers already make it possible to diagnose several neurological diseases *in vivo* and at preclinical stages, yielding topographic, and quantitative data about the target. As a result, they can be used for assessing patients' eligibility for new treatments, or for treatment follow-up. The aim of the present review was to map major innovative radiotracers used in neuroscience, and explain their contribution to clinical research. We categorized them according to their target: dopaminergic, cholinergic or serotoninergic systems, β-amyloid plaques, tau protein, neuroinflammation, glutamate or GABA receptors, or α-synuclein. Most neurological disorders, and indeed mental disorders, involve the dysfunction of one or more of these targets. Combinations of molecular imaging biomarkers can afford us a better understanding of the mechanisms underlying disease development over time, and contribute to early detection/screening, diagnosis, therapy delivery/monitoring, and treatment follow-up in both research and clinical settings.

## Introduction

Molecular imaging is the visualization, characterization, and measurement of biological processes at the molecular and cellular levels in humans and other living systems (1). Over the past few years, rapid improvement in molecular imaging has led to gain in specificity and quantification helpful for early diagnosis and disease follow-up, particularly within the field of neurology. A key advantage of *in vivo* molecular imaging is its ability to identify pathological processes without the need for invasive biopsies or surgical procedures (2).

This imaging technique is currently performed with positron emission tomography (PET) and single-photon emission tomography (SPECT). Several PET and SPECT radiotracers have been developed for neuroimaging applications. The first ones, namely 123I-labeled amines, 99mTc-hexamethylpropyleneamine-oxime (99mTc-HMPAO), and 99mTc-ethyl cysteinate dimer (99mTc-ECD), were developed in the 1990s to measure regional cerebral blood flow in the presurgical evaluation of patients with refractory partial epilepsy (3). The 2000s saw the advent of PET with the use of fluorine-18 fluorodeoxyglucose ([18F]FDG) in clinical routine, for the assessment of cerebral glucose metabolism. As such, it has also been used in the preoperative evaluation of partial epilepsy, but its indications equally include the early diagnosis and differential diagnosis of dementing disorders, differential diagnosis of cerebral space-occupying lesions, detection of viable tumor tissue (recurrence), non-invasive grading, and differentiation between Parkinson's disease and atypical Parkinsonian syndromes (4).

During the past decade, advances in molecular imaging have enabled scientists to focus on specific brain targets, such as receptors, neurotransmitter transporters, or abnormal protein deposits. There are a growing number of radiotracers, which are regarded as valuable tools for many medical imaging applications, including early detection, diagnosis, and treatment follow-up (2). New imaging biomarkers (e.g., amyloid peptide) allow for the diagnosis of neurological diseases at an early stage, thus contributing to the emergence of the concept of preclinical disease (5, 6). Several PET and SPECT radiotracers are used for both routine clinical applications and research that aim to improve the prevention, diagnosis and treatment of brain diseases. For instance, molecular imaging biomarkers can be used for treatment follow-up, or for selecting patients to be included in clinical trials, or for exploring the neurobiological underpinnings of disease progression.

The aim of the present review was to map out the main innovative radiotracers used in neurology, and explain their role in clinical research. We did not explore 11C-labeled tracers in any depth, as they are not widely used for clinical purposes, owing to their short half-life (20 min). We classified the radiotracers according to their target.

## Dopaminergic System

Today, the main class of radiotracers targeting neurotransmission is the one that enables the dopaminergic pathways to be explored (7). These molecules allow for the imaging of nigrostriatal neurons and dopamine receptors. They are used as PET or SPECT radiotracers and assist with the diagnosis of Parkinson's disease (PD), other Parkinsonian syndromes, and Lewy body dementia (LBD) (8).

The first radiotracer to be introduced for the non-invasive assessment of nigrostriatal terminals was [18F]-DOPA in 1983 (9). This radiotracer reflects the activity of aromatic amino acid decarboxylase (AADC), an enzyme that converts L-DOPA to dopamine, through its subsequent accumulation in the dopamine neurons (10). Striatal F-DOPA uptake has been found to be closely related to the nigral cell count (11), except at the beginning of the disease as a consequence of functional compensation (F-DOPA uptake is preserved while motor symptoms can be already presents) (12). This molecule has a history of more than 30 years in clinical research and for the diagnosis of PD. However, in the past decade, the clinical practice led to prefer instead tracers targeting the plasma membrane dopamine transporter (DAT). The latter is easier to use and has a high sensitivity for detecting presynaptic dopaminergic degeneration at early-stage of PD. F-DOPA has recently regained interest in the context of regenerative therapy for PD such as the implantation of dopamine cells or the infusion of drugs with regenerating effects into the striatum (13, 14). The purpose of this therapy is to regenerate the dopaminergic presynaptic function by converting L-DOPA to dopamine. In that cases, DAT tracers are considered to be less relevant for measuring therapeutic response than F-DOPA.

As mentioned above, the second presynaptic dopaminergic target is the DAT, located on dopamine nerve cell terminals. In contrast to the AADC, the DAT is only expressed within dopamine neurons. However, the ligands used for its imaging may also bind to related transporters, such as the serotonine reuptake transporter (SERT) or the norepinephrine reuptake transporter (10). In SPECT imaging, several radiotracers have been developed. The most commonly used are the two cocaine derivatives: [123I]-βCIT and [123I]-FPCIT (8). Compared with [123I]-βCIT, [123I]-FPCIT has better selectivity for DAT vs. SERT, and due to its lower DAT affinity, it has better kinetic properties, with a striatal peak time at 148 min after intravenous injection (15). Although direct comparison of FP-CIT SPECT and F-DOPA PET has shown that both FP-CIT SPECT scans and F-DOPA PET scans are able to distinguish patients with PD from healthy controls with high levels of sensitivity and specificity, the decrease in [123I]-βCIT binding more closely mirrors the reduction in dopaminergic neurons than the decrease in F-DOPA uptake does, suggesting that β-CIT binding is a better index of dopaminergic neuron loss (16). These different sensitivity of the two tracers to a reduction in dopamine transmission is linked to differing degrees of decrease in the striatal uptake of the two tracers, with less striatal FP-CIT uptake than F-DOPA uptake at the early phase of disease (17). [123I]-FPCIT was licensed as DaTSCAN (Amersham Health) in Europe in 2000, and is now a frequently used SPECT radioligand in clinical routine, particularly as an ancillary tool for diagnosing patients with movement disorders, but also in clinical research (15). In the latter context, [123I]-FPCIT has been used in numerous studies seeking to determine the sensitivity and specificity of this tracer in the differentiation of several causes of dementia (18), as well as to study variations in DAT density after different treatments, such as antipsychotics in patients with schizophrenia (19), or psychotherapy in individuals with depression (20).

Tropane derivatives have also been labeled with 99mTc: TRODAT-1 has been compared with F-DOPA in patients with PD (21), and may represent a reliable alternative. 99mTc-labeled ligands are less expensive, and may therefore be more easily accessible, and more suitable for routine use (22–24).

Another tracer has been developed to image the DAT: PE2I. Like FP-CIT and β-CIT, this molecule is a cocaine derivative, which can be labeled with iodine-123 or−125, carbon-11, or tritium (25). This ligand has about a 30-fold higher affinity for DAT than for SERT, and its lower affinity for DAT makes [123I]-PE2I kinetics better than that of [123I]-FPCIT, with a striatal peak time of 30–60 min. However, despite its favorable properties, [123I]-PE2I is not currently licensed as a SPECT radioligand for clinical use (15).

The excellent properties of PE2I mentioned above recently were exploited to develop a new DAT tracer: LBT-999, exploited by Zionexa, which could be used in future PET explorations using fluorine-18 (26–28). Because of its higher resolution, PET imaging is more useful than SPECT for accurate *in vivo* quantification of DAT density. LBT-999 is a phenyltropane derivative that has demonstrated its suitability for *in vivo* quantification of DAT in non-human primates (29). An *in vivo* kinetic study in baboons confirmed that LBT-999 brain uptake is fast, high, and mainly located in the putamen and caudate, with peak uptake in these regions at 30 min postinjection.

A third way of investigating the function of dopamine terminals is to measure the density of vesicular monoamine transporter (VMAT2), which is responsible for taking up neurotransmitters into presynaptic secretory vesicles. Although a majority of VMAT2 are expressed in dopaminergic terminals, this transporter is also located in various monoaminergic neurons, and is involved in the vesicular trapping of a wide variety of neurotransmitters including dopamine, serotonin, norepinephrine, and epinephrine. This target can be investigated with [11C]-DTBZ, or more recently with fluorinated analog [18F]-AV-133, by PET (30). This presynaptic marker follows very typical patterns in several neurodegenerative diseases affecting dopaminergic function, such as PD, LBD, multiple system atrophy (MSA), progressive supranuclear palsy (PSP), and corticobasal syndrome (CBS). Their uptake/binding is altered in several brain areas, depending on the disease and its stage (31). In contrast to AADC activity or DAT binding, it has been suggested that VMAT2 activity is less inclined to changes induced by medication or compensatory mechanisms. However, VMAT2 activity can be impacted by the amount of vesicular dopamine, competing at the recognition site. Hence, the level of VMAT2 binding may decrease with levodopa administration (32). These tracers have a future in early detection/screening, diagnosis, and neuroprotective treatment follow-up of these neurodegenerative diseases, as well as in the monitoring of neural grafted cells after transplantation (8).

**Figure 1:**
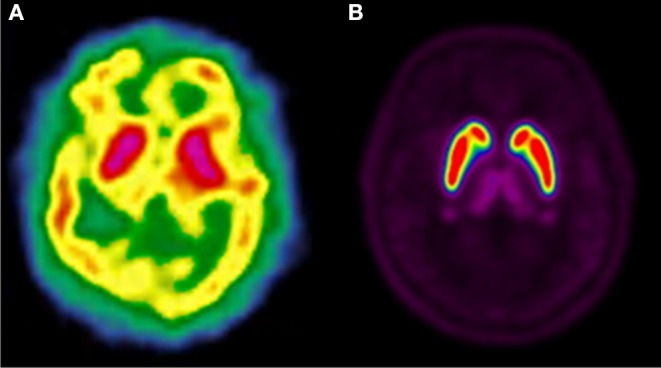
Comparison of [123I]-IBZM image **(A)** and [18F]-fallypride image **(B)** within the same individual.

Dopaminergic neurotransmission can also be explored by visualizing postsynaptic D2 receptors. The binding potential of these receptors can be assessed using SPECT with the ligands [123I-IBZM and 123I]-IBF, as well as PET with [11C]-raclopride and [18F]-fallypride as radiotracers (Figure 1) (8, 31). The concomitant study of DAT and D2 receptors may improve the diagnostic value of molecular imaging in differentiating between PD and other parkinsonian syndromes (33, 34). Nowadays, however, the measurement of cardiac [123I]-MIBG uptake remains the most frequently used technique to differentiate PD and MSA (35). Molecular imaging of dopamine D2 receptors has also been used to study dopamine's role in drug abuse and addiction (36), and to evaluate several neuropsychiatric disorders (37).

**Figure 2:**
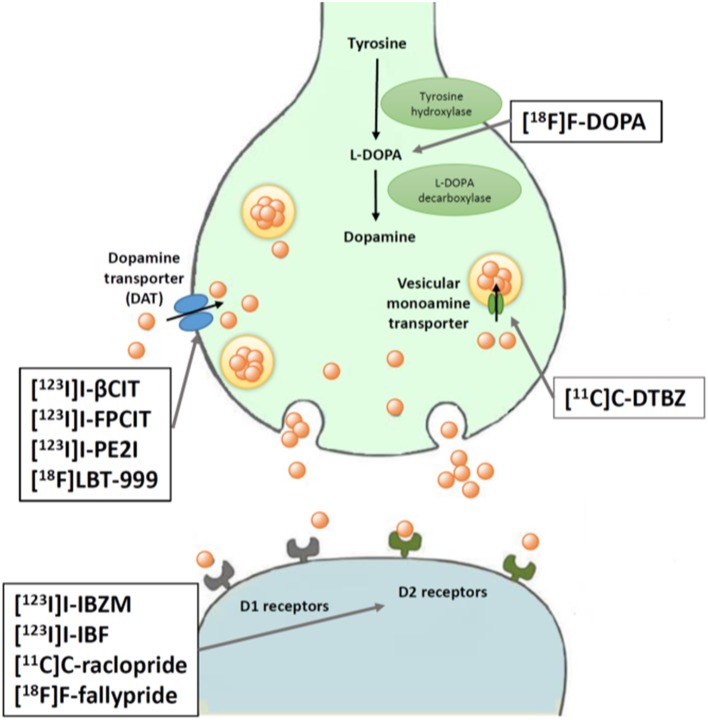
Schematic illustration of PET and SPECT techniques for assessing presynaptic and postsynaptic dopaminergic targets. L-DOPA is converted to dopamine by DOPA decarboxylase, then stored in vesicles by a vesicular monoamine transporter. Dopamine reuptake into presynaptic neurons occurs via a dopamine transporter (DAT). Two different types of dopamine receptors are expressed on postsynaptic neurons: D1 and D2.

Key features of all these tracers are summarized in Figure 2 and Table 1.

**Table 1:** Main SPECT and PET dopaminergic tracers, molecular strucures, pharmacological properties, and examples of clinical studies.

**Compounds**	**Imaging modality**	**Target/ measure**	**Affinity (nM)**	**Clinical studies**	**Compounds**	**Imaging modality**	**Target/ measure**	**Affinity (nM)**	**Clinical studies**
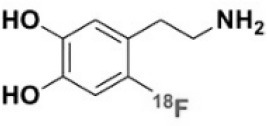 [^18^F]-DOPA	PET	AADC activity	Uptake	PD (10, 13, 14) LBD (38, 39) MSA (40) PSP (41)	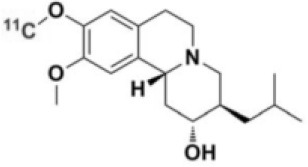 [^11^C]-DTBZ	PET	VMAT2 density	K_i_ = 2 (42)	PD (43, 44) LBD (44, 45) MSA (46, 47)
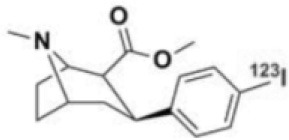 [^123^I]-βCIT	SPECT	DAT density	K_i_ = 27 ± 2 (DAT) K_i_ = 3 ± 0.2 (SERT) K_i_ = 80 ± 28 (NET) (48)	PD (49–52) LBD (53) MSA (34)	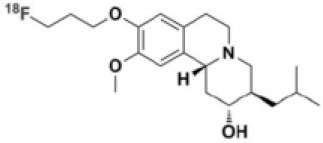 [^18^F]-AV133	PET	VMAT2 density	K_d_ = 0.19 (striatum) K_d_ = 0.25 (hypothalamus)(54)	PD (55) LBD (56)
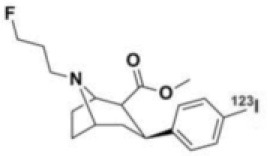 [^123^I]-FPCIT	SPECT	DAT density	K_i_ = 3.5 (DAT) K_i_ = 9.7 (SERT) (48)	PD (57–59) LBD (60) MSA (61) PSP (58)	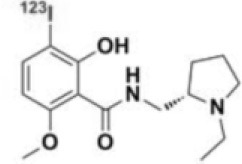 [^123^I]-IBZM	SPECT	D2 receptors density	K_d_ = 3.1 ± 0.62 K_i_ = 0.32 (D2) K_i_ = 4143 (D1) (62)	MSA (63, 64) PSP (63, 65)
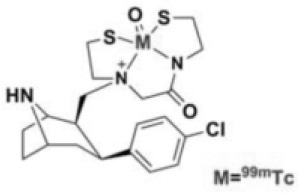 [^99m^Tc]-TRODAT-1	SPECT	DAT density	K_i_ = 14.1 ± 2.1 (DAT) K_i_ = 360 ± 44 (SERT) (23)	PD (21, 66)	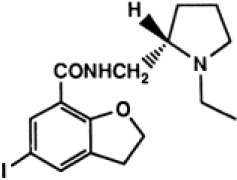 [^123^I]-IBF	SPECT	D2 receptors density	K_d_ = 0.106 ± 0.015 K_i_ = 0.015 ± 0.002 (D2) K_i_ = 820 ± 164 (D1) (67)	MSA (68) PSP (68, 69)
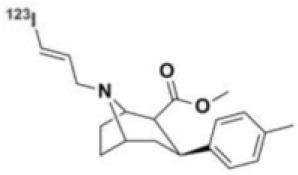 [^123^I]-PE2I	SPECT	DAT density	K_i_ = 17 ± 7 (DAT) K_i_ = 500 ± 30 (SERT) K_i_ >1000 (NET) (25)	PD (70, 71) LBD (72) PSP (73)	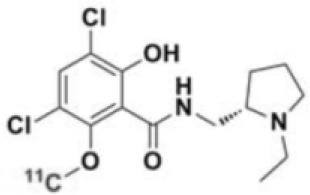 [^11^C]-raclopride	PET	D2 receptors density	K_i_ = 7.5 (74)	MSA (75, 76)
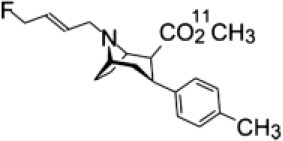 [^18^F]-LBT-999	PET	DAT density	Kd = 9.15 ± 2.8 (DAT) IC_50_ >1000 (SERT and NET) (26)	PD (77)^*^	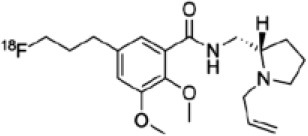 [^18^F]-fallypride	PET	D2 receptors density	IC_50_ = 0.6 (78)	Epilepsy (79)

*Kd, dissociation constant; Ki, inhibition constant; IC50, half maximal inhibitory concentration*.

* *Preclinical study*.

## Amyloid Imaging

β-amyloid (Aβ) plaques in the brain are one of the key histopathologic lesions of Alzheimer's disease (AD) (80). Advances in the understanding of the physiopathology of AD suggest that progressive amyloid accumulation begins during the presymptomatic phase, followed by synaptic dysfunction, tau-mediated neuronal injury, a reduction in brain volume, and finally the emergence of cognitive symptoms, followed by a clinical syndrome of overt dementia (81). This suggest that Aβ imaging is a critical step for the early diagnosis of AD.

These deposits were first imaged in PET in 2002, using a thioflavin-T derivative: 11C-Pittsburgh compound B ([11C]-PIB) (82). Although this is the best known compound, its use is restricted to the research field, owing to the short half-life of 11C. Numerous studies have showed that [11C]-PIB binds to Aβ plaques in several cortical regions in patients with AD (82–84). [11C]-PIB binding is correlated with a reduction in cerebrospinal fluid Aβ42 (85), cerebral atrophy (86), and episodic memory impairment in apparently healthy elderly individuals and those with mild cognitive impairment (MCI) (87). These studies have paved the way for the development of several Aβ plaque PET tracers labeled with 18F. To date, three radiopharmaceuticals with equivalent diagnostic performances have been authorized by the European Medicines Agency and the US Food and Drug Administration: 18F-florbetapir, 18F-florbetaben, and 18F-flutemetamol (88) (Table 2).

**Table 2:** Main amyloid PET tracer, molecular structures, pharmacological properties, and examples of clinical trials in AD.

**Compounds**	**Target/ measure**	**Affinity (nM)(88)**	**Clinical studies in AD**
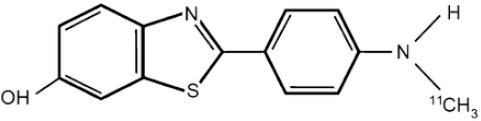 [^11^C]-PIB	Aβ plaques (fibrillar oligomer)	K_i_ = 0.9	(82–84)
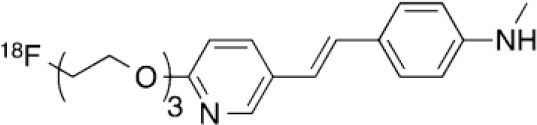 [^18^F]-florbetapir	Aβ plaques (aggregated form)	K_i_ = 2.2	(89–92)
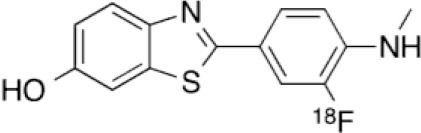 [^18^F]-flutemetamol	Aβ plaques (soluble form)	K_i_ = 0.7	(93–95)
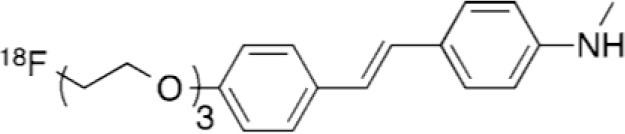 [^18^F]-florbetaben	Aβ plaques (aggregated form)	K_i_ = 2.4	(96–99)

The clinical criteria that are currently used for AD diagnosis have variable specificity and sensitivity, with pooled averages of 70 and 81% (100). A recent review assessing studies published from January 1980 to March 2014 on the diagnostic utility of these three radiotracers demonstrated a pooled weighted sensitivity and specificity of 89.6% and 87.2% for florbetapir, and 89.3 and 87.6% for florbetaben in differentiating patients with AD from age-matched normal controls (101). These results suggest that 18F-labeled tracers have better sensitivity and specificity than clinical diagnosis and other biomarkers commonly used in practice (89), and are comparable to 11C-PiB. They have also been shown to have good patient tolerability (96). However, the extent and distribution of Aβ plaques and amyloid PET tracer binding in patients are only moderately correlated with patterns of neurodegeneration and cognitive deficits (102–104). This suggests that Aβ deposition, which is a prerequisite for diagnosing AD, is just the starting-point of a cascade of other neuropathological events, rather than the actual driver of neurodegeneration and clinical disease progression (105).

In this respect, these tracers are chiefly useful for their good negative predictive value. A negative scan (i.e., amyloid burden undetectable or extremely low) is considered to be incompatible with a diagnosis of AD. Although a moderate-to-high amyloid plaque density may point to AD, a positive test is not sufficient to diagnose this disorder, especially in elderly participants. It was in this context that the Society of Nuclear Medicine and Molecular Imaging and the Alzheimer's Association delineated “appropriate use criteria” in 2013, identifying three clinical circumstances in which amyloid PET imaging is recommended to clarify the diagnosis: “Patients with persistent or progressive unexplained mild cognitive impairment”, “Patients satisfying core clinical criteria for possible (as opposed to probable) Alzheimer's disease (i.e., atypical clinical course or etiologically mixed presentation)”, and “Patients with atypically young-onset dementia” (106).

In spite of its excellent diagnostic capacity, the use of amyloid PET imaging in clinical practice is still limited. However, this technique has proved extremely useful in clinical trials. Currently, the treatment options for AD are limited to symptomatic drugs, with no attenuation of the ultimate prognosis (107). Numerous studies are being conducted to find new treatments, as well as to better understand the physiopathology of AD. One of the research approaches to develop new treatments involves targeting the two pathological features associated with AD, namely senile plaques (Aβ) and neurofibrillary tangles (NFTs) composed of aggregates of hyperphosphorylated tau protein in paired helicoid filaments (PHF). According to the amyloid cascade hypothesis, toxic plaques are the earliest manifestation of the disease, a notion supported by evidence of Aβ up to 20 years prior to the onset of symptoms (107). Two main classes of medication are under development as a result: monoclonal anti-amyloid antibodies, and inhibitors of pathogenic cleavage of the amyloid precursor protein (APP). PET amyloid radiotracers in clinical trials evaluating the therapeutic potential of these medications are used for selecting and including patients with significant Aβ, or monitoring disease progression under treatment (108). For example, in an amyloid-based immunotherapy study, PET imaging used for treatment follow-up suggested that anti-amyloid antibodies were more effective in the early stages of amyloid accumulation (108). Soon after this discovery, another study was therefore conducted to study the effect of this class of medication in patients with few or no symptoms (MMSE 20–26) but positive amyloid PET imaging (109). This study failed to show a significant difference in cognitive outcomes between the study group and asymptomatic controls; however other drug studies with similar design using amyloid tracer PET imaging in asymptomatic patients with AD are ongoing.

## Tau Imaging

As previously indicated, several studies have reported that Aβ burden is only moderately correlated with glucose hypometabolism, disease severity, progression, and clinical presentation. Furthermore, clinical trials assessing monoclonal anti-amyloid antibodies have mostly failed to show a clinical benefit in AD. The other main histopathological figure of AD, abnormal tau protein aggregates, has therefore be considered with much interest. Several PET radiopharmaceuticals have therefore been developed to accurately target abnormal tau protein conformations. NFTs composed of aggregated hyperphosphorylated tau in paired helicoid filaments are one of the two key neuropathological substrates of AD, along with Aβ plaques (110). Whereas, Aβ levels stabilize at an early stage, the presence and extent of NFTs and neuronal injury increase in parallel with disease duration and severity of symptoms (111). Moreover, tau has been found to be more closely related to memory decline in post mortem studies of AD than amyloid pathology (112). Abnormal aggregation of tau protein has also been observed in the pathophysiology of other neurodegenerative diseases, including frontotemporal dementia (FTD), CBS, PSP and, to a smaller extent, LBD; the abnormal conformation of tau in these diseases are distinct from that observed in AD which involves paired helicoid filaments (PHF). These pathologies are collectively known as tauopathies. These tauopathies differ by the isomeric form and ultrastructural morphology of aggregated tau, affected brain regions, and spatial patterns of tau accumulation (110).

Over the past few years, six promising tau imaging agents have been developed: [11C]-PBB3, [18F]-AV-1451 (or flortaucipir, previously known as T807), [18F]-T808, and the THK family [18F]-THK523, [18F]-THK5105, and [18F]-THK5351. These radiotracers have been synthesized, using structure–activity relationship software, from N-benzylidene-benzohydrazide compounds used for the detection of tau-paired helical filament (PHF) (88).

One of the first radiotracers developed for tau imaging was [18F]-FDDNP. This tracer is rapidly metabolized in hydrophilic compounds that cross the blood brain barrier (BBB), resulting in non-specific binding and therefore significant background noise. Furthermore, this tracer is not specific to NFTs, but also has an affinity for Aβ plaques, meaning that it is not the best choice for tau assessment (88, 113, 114).

The first tau-selective radioligand, [18F]-THK523 was synthesized by Okamura et al. (115), and its selectivity for phosphorylated tau was confirmed in post mortem studies, as well as in several *in vitro, ex vivo*, and *in vivo* experiments (116). However, this tracer is not able to bind to tau aggregates in non-AD tauopathies such as PSP and CBD, and is characterized by high retention in white matter (117, 118). New THK compounds have since been developed: [18F]-THK5105, [18F]-THK5117, and [18F]-THK5351. The latter has better kinetics, less white matter binding, and a higher affinity for tau than [18F]-THK523 (119). However, it also binds to MAO-B sites, and has a lower binding level in AD than AV-1451 does (110).

[11C]-PBB3 is another tau radiotracer with a high affinity for NFTs, a low level of white matter binding, good BBB penetration and rapid washout. The peculiarity of [11C]-PBB3 is its affinity for the tau isoforms of several non-AD tauopathies. However, it metabolizes to a radiolabeled compound that can cross the BBB, thus limiting its quantification (110).

[18F]-T807 ([18F]-AV1451 developed by Lilly Research Laboratories) and [18F]-T808 belong to the benzimidazole pyrimidine family. They have a nanomolar affinity for the tau PHF found in AD, and are 25 times more selective for tau PHF than for Aβ (120, 121). Today, [18F]-AV-1451 is the most widely used tau radioligand. Like [11C]-PBB3, it has low retention in white matter. Several clinical studies have shown a close correlation between [18F]-AV1451 binding and the neuropathological stages of tau (122), cognitive decline and tau levels in cerebrospinal fluid (123, 124). However, a recent autoradiographic evaluation of AV1451 reported a lower level of binding in non-AD tauopathies, as well as off-target binding in the basal ganglia and substantia nigra in the absence of tau pathology (125).

Recently, another radioligand ([18F]MK-6240, developed by Merck laboratories) was administered to patients with AD with promising results. This tracer showed a high specificity and selectivity for NFTs, good pharmacokinetic properties, and no apparent off-target binding, in contrast to [18F]-AV-1451 (110, 126–128).

As a link has been demonstrated between NFTs and AD symptoms, tau PET tracers are increasingly being used in AD clinical trials, especially those investigating drugs to reduce the tau or Aβ burden (129), such as Aβ monoclonal antibodies. The indirect effect of reducing Aβ on the rate of PHF deposition downstream further supports the amyloid hypothesis, and tau PET imaging may highlight the presumptive disease-modifying impact of these drugs. Furthermore, as tau monoclonal antibodies are designed and investigated, tau PET imaging will be helpful in demonstrating and quantifying the engagement of the molecular target. Many trials currently use cerebrospinal fluid (CSF) biomarkers of tau and phosphorylated tau to detect target engagement, but there are few data on how CSF biomarkers and tau PET imaging correlate. Tau PET imaging may also help to confirm that changes in tau deposition are correlated with clinical disease progression (130). Several tau vaccines have shown efficacity and safety in animal models (131). In a recent study, an anti-tau drug exhibited a good safety profile and even stimulated a positive immune response in human patients (132). Several other early-phase trials of drugs that target tau protein are currently underway, although the results are yet to be published (133).

In this context, like amyloid tracers, tau radioligands (summarized in Table 3) have an important role to play in clinical studies assessing new treatments and measuring disease progression.

**Table 3:** Main tau PET tracers, molecular structures, pharmacological properties, and examples of clinical studies.

**Compounds**	**Target/measure**	**Affinity (nM)**	**Comments**	**Clinical studies**
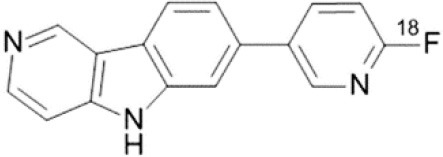 [^18^F]-flortaucipir(AV1451, T807)	PHF-tau	K_d_ = 14.6 (88)	25 time more selective for tau PHF than for Aβ. Low retention in white matter. Off-target binding has been reported in the basal ganglia and substantia nigra in the absence of tau pathology.	AD (134–137)
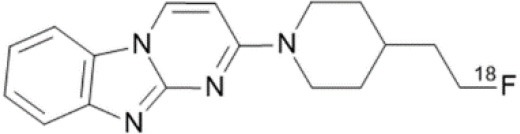 [^18^F]-T808	PHF-tau	K_d_ = 22 (138)	Slow metabolic defluorination (139)	AD (120)
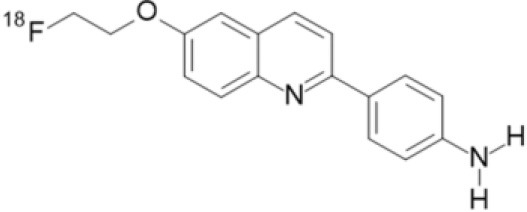 [^18^F]-THK523	PHF-tau	K_d_ = 86 (88)	12-fold selectivity for tau over Aβ. High retention in white matter.	AD (118)
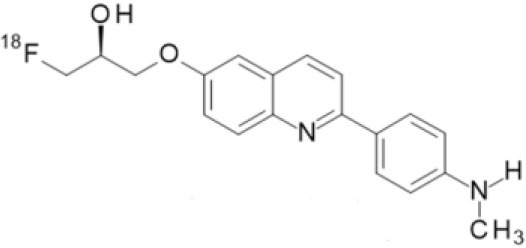 [^18^F]-THK5117	PHF-tau	K_d_ = 5.19 (88)	High binding selectivity to tau over Aβ. Substantial white matter binding.	AD (140)
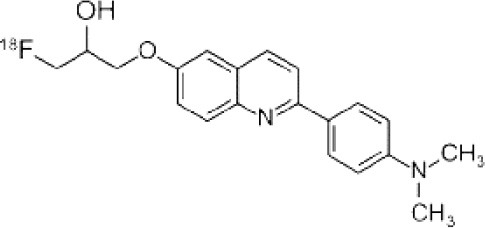 [^18^F]-THK5105	PHF-tau	K_d_ = 2,63 (88)	Higher binding affinity to tau fibrils than to Aβ1–42 fibrils (K_d_ = 35.9 nM) (141) Substantial white matter binding.	AD (115, 142)
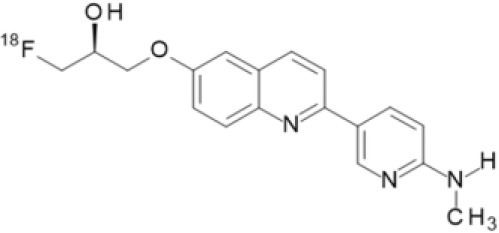 [^18^F]-THK5351	PHF-tau	K_d_ = 2.9 (88)	Low binding affinity for white matter, and rapid pharmacokinetics. It also bind to MAO-B sites (110)	AD (119)
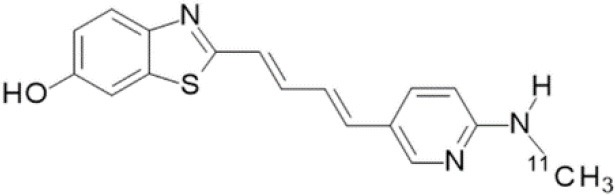 [^11^C]-PBB3	PHF and non-PHF tau	K_d_ = 100 (88)	40–50 fold higher affinityfor NFTs than for Aβ, rapid washout, minimal white matter binding, but it metabolizes to a radiolabeled compound that cross the BBB (110)	AD (143), PSP (144), Amyotrophic lateral sclerosis/parkinsonism dementia complex [ALS/PDC (145)]
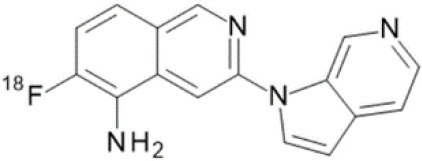 [^18^F]-MK6240	PHF-tau	K_i_ = 0.36 ± 0.8 (88)	Poor affinity for Aβ plaques (K_i_ = 10 μM) (127) No apparent off-target binding.	AD (128)

## Neuroinflammation

Neuroinflammation is an inflammatory and adaptive response within the central nervous system, and depends on several processes mediated by neuronal cells such as astrocytes, as well as by non-neuronal cells such as the brain's resident macrophages and microglia.

Although initiation of an inflammatory response may be beneficial in response to injury of the nervous system, chronic or maladaptive neuroinflammation can have harmful outcomes in many neurological diseases. During inflammatory processes, cytokines, chemokines and reactive oxygen species (ROS) are produced by glial cells, and all these molecules can be targeted by molecular imaging (146).

**Figure 3:**
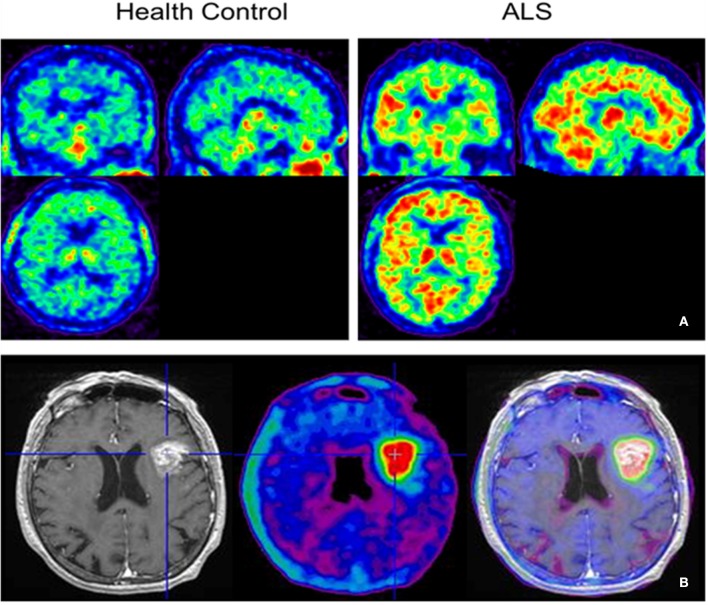
[18F]-DPA-714 images obtained from two clinical studies: **(A)** Comparison between Amyotrophic Lateral Sclerosis (ALS) patients and healthy individuals, and **(B)** stroke patient.

The main target for imaging neuroinflammation is currently translocator protein (TSPO) overexpression in activated microglia. TSPO is a highly hydrophobic protein that is mainly situated in the outer mitochondrial membrane. Classically not present in healthy brain parenchyma, TSPO has been widely identified in microglial cells in dementia neuropathology, which involves neuroinflammatory processes and microglial activation. The most widely used TSPO PET radiopharmaceutical tracer used to be [11C]-(R)-PK11195. A new generation of fluorinated tracers has been developed in the past decade (147, 148), with different compound families such as phenoxyarylacetamides derivatives ([18F]-FEDAA1106, [18F]-FEPPA, [18F]-PBR06), imidazopyridine derivatives ([18F]-PBR111), and pyrazolopyrimidine derivatives ([18F]-DPA-714) (Figure 3). However, while these fluorinated compounds have turned out to be more sensitive and specific, with a clear improvement in the signal-to-noise ratio, a major additional problem has been identified, in the shape of a polymorphism in the TSPO gene (rs6971) that affects TSPO binding, with a significant impact on its visualization and its quantification. To circumvent this drawback, a new generation of rs6971-insensitive TSPO radioligands have been developed, such as flutriciclamide ([18F]-GE180) (149), and this latest generation of tracers is currently under evaluation (150).

Other PET tracers of gliosis have been tested, such as [11C]-DED, which binds to MAO-B, and some results in transgenic animals (151) seem to indicate that gliosis occurs early in AD and precedes the deposition of Aβ senile plaque. Cyclooxygenase was also investigated by Shukuri et al. (152), who showed that [11C]-ketoprofen methyl ester, a specific tracer of COX1, is useful for imaging cerebral inflammation in injured rats, with very different kinetics from TSPO tracers. However, a study in humans with this ketoprofen derivative in 2016 (153) failed to yield positive results, suggesting that COX1 expression is more specific for acute inflammation than for chronic inflammation.

Recently, researchers have shown increasing interest in the ROS system. In cardiology, [18F]-DHMT makes it possible to visualize early ROS activation prior to ventricular function deterioration induced by doxorubicin toxicity (154). In neurology, [18F]-ROStrace, a tracer trapped in the brain when it is metabolized by ROS is currently being assessed in models of AD, PD and other neurodegenerative diseases (155).

These tracers are summarized in Table 4.

**Table 4:** Main PET tracers for neuroinflammation imaging, molecular structures, pharmacological properties, and examples of clinical studies.

**Compounds**	**Target/measure**	**Affinity (nM)**	**Clinical studies**	**Compounds**	**Target/ measure**	**Affinity (nM)**	**Clinical studies**
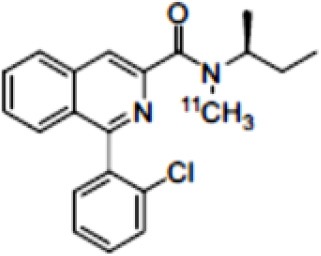 [^11^C]-(R)-PK11195	TSPO density	K_i_ = 9.3 in rat (156)	AD (157, 158), PSP (157), multiple sclerosis (MS), PD, ALS, HI, Rasmussen's encephalitis, Herpes encephalitis, Schizophrenia (156)	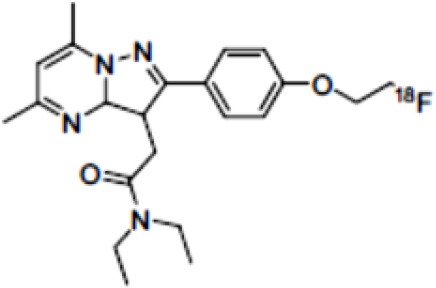 [^18^F]-DPA714	TSPO density	K_i_ = 7.0 in rat (156)	AD (159, 160)
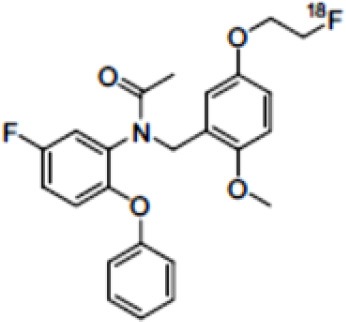 [^18^F]-FEDAA1106	TSPO density	K_i_ = 0.078 in rat (156)	AD (161) MS (162)	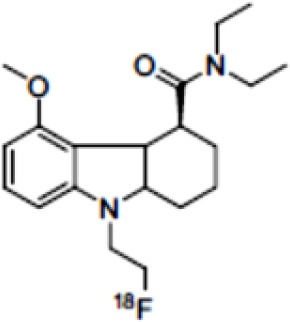 [^18^F]-GE180	TSPO density	K_d_ = 0.87 in rats (163)	MS (164)
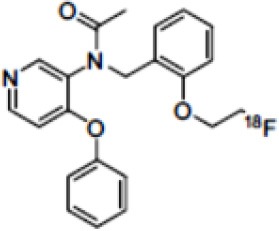 [^18^F]-FEPPA	TSPO density	K_i_ = 0.07 in rat (156)	AD (165)	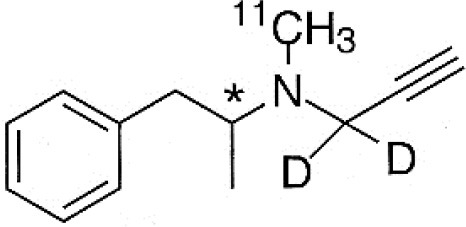 [^11^C]-DED	MAO-B activity	NA	AD (166)
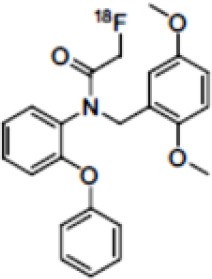 [^18^F]-PBR06	TSPO density	K_i_ = 0.30 in monkey (156)	MS (167)	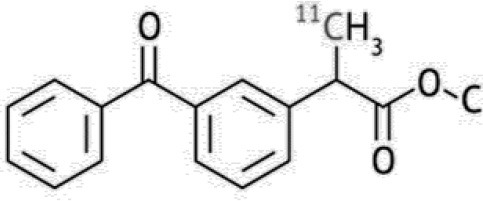 [^11^C]-ketoprofen methyl ester	COX-1	IC_50_ = 47 (COX-1) IC_50_ = 2.9μM (COX-2) (152)	AD (153)
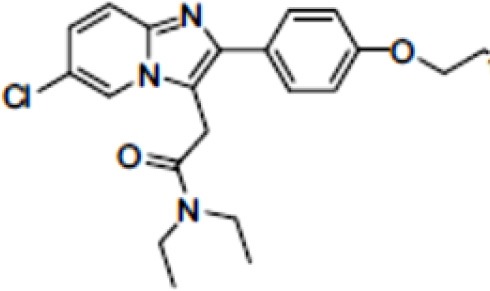 [^18^F]-PBR111	TSPO density	K_i_ = 3.70 in rat (156)	MS (168) Schizophrenia (169) Epilepsy (170)	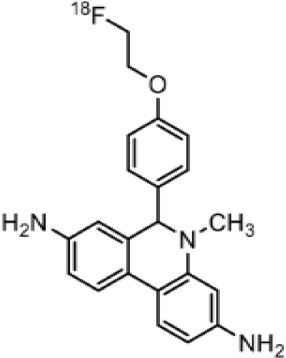 [^18^F]-ROStrace	ROS activity	NA	Preclinical studies

## Glutamate Receptors

Glutamate is the most abundant excitatory neurotransmitter, and glutamate receptors (GluRs) are implicated in plenty of neurological functions within the central nervous system (CNS). GluRs are classified into two groups: ionotropic receptors (iGluRs) and metabotropic receptors (mGluRs). iGluRs form ligand-gated ion channels and are divided into three subtypes based on their pharmacological properties: NMDA (N-methyl-D-aspartate receptors, NMDARs), AMPA (α-amino-3-hydroxy-5-methylisoxazole-4-proprionic acid) receptors, and kainate receptors. mGluRs are G-protein coupled receptors and include eight receptor subtypes, classified into three groups according to their sequence homology, signal transduction, and pharmacological profiles. Group I is comprised of mGluR1 and mGluR5, group II includes mGluR2 and mGluR3, and group III contains mGluR4, mGluR6, mGluR7, and mGluR8 (171). A dysfunction of these receptors may be involved in the pathophysiology of numerous brain disorders. Several PET and SPECT probes have been developed for GluRs imaging (Table 5).

**Table 5:** Main SPECT and PET glutamatergic tracers, molecular structures, pharmacological properties, and examples of clinical studies.

**Compounds**	**Imaging modality**	**Target/ measure**	**Affinity (nM)**	**Clinical studies**	**Compounds**	**Imaging modality**	**Target/ measure**	**Affinity (nM)**	**Clinical studies**
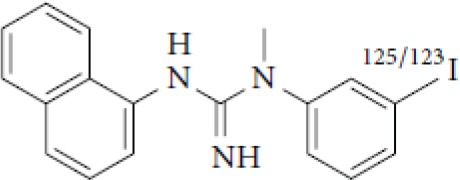 [^123/125^I]-CNS-1261	SPECT	NMDARs density	K_i_ = 4.2 (171)	Schizophrenia (172, 173)	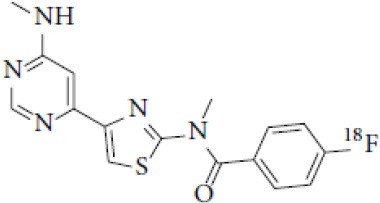 [^18^F]-FIMX	PET	mGlu1Rs density	IC_50_ = 1.8 (171)	(174)
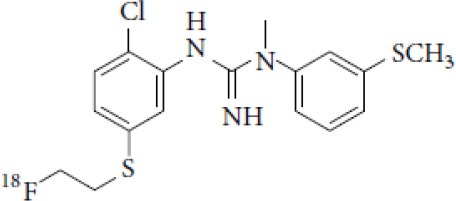 [^18^F]-GE-179	PET	NMDARs density	K_i_ = 2.4 (171)	-	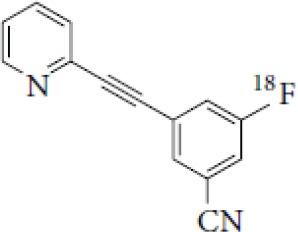 [^18^F]-FPEB	PET	mGlu5Rs density	K_i_ = 0.2 (171)	PD (175), alcohol dependence (176), depression (177), autism (178)
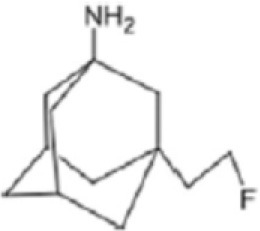 [^18^F]-FNM	PET	NMDARs density	K_i_ = 3500 (179)	Tourette's syndrome (GlutaTour project, ToNIC TMBI)	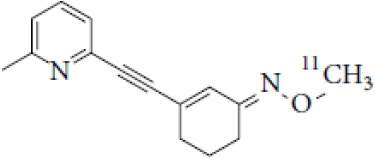 (E)-[^11^C-]ABP688	PET	mGlu5Rs density	K_d_ = 5.7 (171)	Cocaine addiction (180), depression (181), FTD (182), alcohol dependence (183)
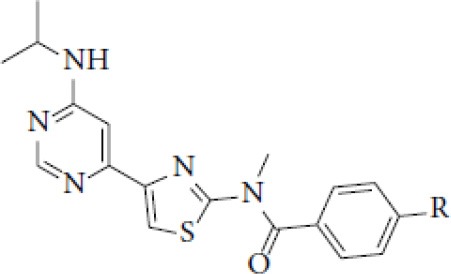 [^11^C]-ITMM: R = O^11^CH3 [^11^C]-ITDM: R = ^11^CH3	PET	mGlu1Rs density	K_i_ = 12.6 ([^11^C]-ITMM) (171)	(184)	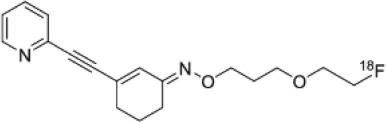 [^18^F]-PSS232	PET	mGlu5Rs density	K_i_ = 1 (E-isomer) (185)	(186)
			K_i_ = 13.6 ([^11^C]-ITDM) (171)	Preclinical studies	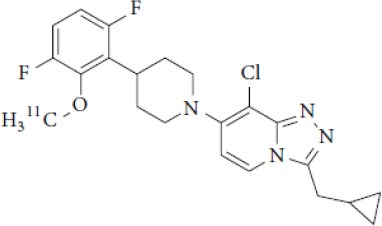 [^11^C]-JNJ42491293	PET	mGlu2Rs density	IC_50_ = 9.2 (171)	(187)

### NMDARs

**Figure 4:**
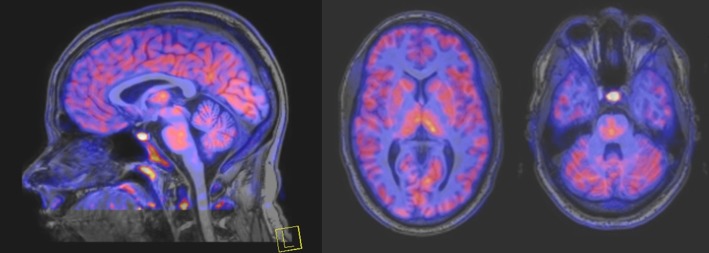
Images from first-in-man injection of [18F]-FNM in a Tourette's syndrome patient (GlutaTour project).

Linked to ligand- and voltage-gated ion channels, NMDARs play an important role in many biological functions, including neurotransmission, neuroprotection, neurodegeneration, long-term potentiation, memory, and neurogenesis (188). These receptors are heteromeric multimers composed of one GluN1 (NR1 subunit) and combinations of GluN2 (NR2 subunits) (189) and GluN3 (NR3 subunits) (190). NR2 subunits come in four subtypes (A D) that determine the type of receptor, with A and B being the most widespread. NR2B subunits, preferentially expressed on primary afferent fibers (PAFs), play a particular role in the transmission of pain messages (191). NMDARs activation requires several types of agonists interacting in cooperation and the simultaneous presence of strong membrane depolarization. Furthermore, NMDARs activation is modulated by extracellular Mg2+, which exerts a voltage-dependent blockade of the open ion channel (192). First, two co-agonists, glutamate and glycine, have to simultaneously bind to their respective sites. Membrane depolarization then causes the release of Mg^2+^ from the channel to allow for the intraneuronal entry of calcium, the starting point for the synthesis of second and third messengers [e.g., prostaglandins and nitric oxide (NO)] (193). Under physiological conditions of synaptic transmission, NMDARs are activated for only brief periods of time. However, in pathological circumstances, their overactivation causes excessive Ca^2+^ influx into nerve cells, and can lead to cell death (194). This abnormal mechanism mediates excitotoxic neuronal injury after acute brain damage (195) and is thought to contribute to disorders of neuronal hyperexcitability (e.g., epilepsy) and chronic neurodegenerative (e.g., AD, Huntington's) (196) and psychotic (197) disorders. Several tracers have been synthesized in order to better understand the physiopathology of these diseases. Most of them are phencyclidine site ligands (PCP) that selectively bind to ion channels in the open and active state. These tracers thus make it possible to visualize only activated NMDARs. Several 123I-, 125I-, 11C-, or 18F-labeled SPECT/PET radiotracers have been developed, based on phencyclidine (PCP), thienylcyclohexyl piperidine (TCP) (198, 199), ketamine (200), memantine (201, 202) or MK-801 (203, 204), as these ligands are known to inhibit the intrachannel PCP sites of NMDARs. Although most of these radiotracers have been found to cross the BBB, none of them have detectable specific binding *in vivo*, owing to high non-specific binding, poor brain retention, or insufficient affinity for the small number of specific binding sites (205, 206). To our knowledge, only few NMDARs radiotracers have been used in human studies. The diarylguanidine analog, [123I]-CNS-1261 exhibited limited success in a clinical study of patients with schizophrenia (207). In PET imaging, despite encouraging results (208), a recent preclinical study using [18F]GE-179 was unable to demonstrate displaceable *in vivo* binding that would have been evidence of an *in vivo* activity-dependent NMDA signal in rats and primates (209–211). Recently, a new [18F]-labeled derivative of memantine, [18F]-fluoroethylnormemantine ([18F]-FNM), was synthesized. *In vivo* evaluation of this novel PET tracer has yielded encouraging results (179, 212), and it had been injected for the first time into humans, in a pilot study to explore the glutamatergic system in patients with Tourette syndrome (GlutaTour project, ToNIC TMBI) (Figure 4).

Other NMDAR binding sites, such as the glycine and NR2B sites located on the receptor's extracellular domain, have been the subject of various studies aimed at developing new tracers. However, radiotracer development for these targets has so far been unsuccessful, owing to the ligands' suboptimum physiochemical and pharmacological characteristics, such as affinity, lipophilicity, stability, BBB penetration and pharmacokinetics (171, 205, 206, 213–215).

### mGluR

#### Group I

Group I mGluRs, predominantly expressed postsynaptically, are involved in modulation of synaptic plasticity, and their activation leads to increased neuronal excitability. They are implicated in the physiopathology of several neurological and psychiatric disorder, such as PD, motor dysfunction, multiple sclerosis, epilepsy and stroke, and are the target of recently developed PET probes (171).

#### mGluR1

mGluR1 are found extensively throughout the brain, but are highly expressed in the cerebellar cortex, hippocampus and thalamus. mGluR1 antagonists have shown promising anxiolytic and antidepressant effects, whereas positive modulators of mGluR1 have been reported to be useful for the treatment of schizophrenia (171). Among all developed molecules to image them, only two radioligands have been injected into humans. The first is [11C]-ITMM. *In vitro* and preclinical studies found that this ligand had high affinity and selectivity for mGluR1, and displayed high brain uptake, with highest uptake in the cerebellum (richest mGluR1 area). This cerebellar uptake has also been observed in human PET studies, however, [11C]-ITMM showed relatively low uptake in the brain regions with modest expression of mGluR1, such as thalamus, hippocampus, and cerebral cortex, making it difficult to examine target density in these regions (184). Nevertheless, [11C]-ITMM could be used to evaluate alterations in cerebellar mGluR1 under pathological conditions, and further clinical studies may be needed to assess the usefulness of this radioligand as a PET probe for mGluR1 quantification. [11C]-ITDM, an analog of ITMM, was considered superior to [11C]-ITMM after *in vivo* studies in monkeys because of its higher regional distribution volume in the mGluR1-rich region (216). To our knowledge, clinical PET studies with this radiotracer have not been published.

Finally, [18F]-FIMX, is the second high affinity mGluR1 radioligand injected into humans. The rank order of this tracer uptake correlated well with mGluR1 expression levels in the human brain, with a highest uptake in the cerebellum (174).

#### mGluR5

mGluR5 are found in the cerebral cortex, hippocampus, accessory olfactory bulbs, and nucleus accumbens (171). In physiological conditions, mGluR5 activates an intracellular cascade by second messenger processes and modulates functions as diverse as memory, anxiety, or learning. It has been demonstrated that the disruption of brain homeostasis in pathological conditions causes hyperactivation of mGluR5, which then contributes to excitotoxicity. mGluR5 dysregulation is therefore implicated in a broad variety of neuropsychiatric disorders and mGluR5 is recognized as a relevant molecular biomarker of glutamate pathology in these diseases. PET imaging of mGluR5 has expanded in recent years and has contributed to go deeper in the pathophysiology of brain diseases and to better evaluate new treatment strategies. Several PET radioligands targeting mGluR5 have been synthetized (205, 217) and the most promising candidates are currently being investigated in several preclinical and clinical studies.

[18F]-FPEB has been developed by Merck Research Laboratories and, regarding its high specificity and selectivity for mGluR5, together with a suitable brain kinetics (218, 219), has been extensively used to investigate mGluR5 density in neurological disorders. In neurology, [18F]-FPEB has shown mGluR5 upregulation in Parkinson's Disease (220), but recent main contributions of [18F]-FPEB imaging are about psychiatry and addictions. Thus, Leurquin-Sterk et al. studied the effects of acute alcohol intake on the glutamatergic system (221), and demonstrated that mGluR5 availability was lower in limbic regions of alcohol-dependent subjects than in healthy controls, suggesting that limbic mGluR5 was involved in a compensatory mechanism helping to reduce craving during abstinence (176). The alteration of mGluR5 availability was also demonstrated in posttraumatic stress disorder, with a higher cortical [18F]-FPEB *in vivo* binding that was positively correlated with avoidance symptoms (222). Besides, [18F]-FPEB PET imaging did not find any mGluR5 contribution in Major Depressive Disorder (177), whereas, considering neurodevelopmental diseases, an increased [18F]-FPEB binding was observed in postcentral gyrus and cerebellum of male individuals with autism Specter disorder (178).

[11C]ABP688 is a selective, high-affinity mGluR5 antagonist widely used in mGluR5 clinical PET imaging (223, 224). Recently, [11C]ABP688 revealed *in vivo* evidence of reduced availability of mGluR5 in behavioral variant frontotemporal dementia (182) and in focal cortical dysplasia, in tissue resected from epilepsy patients (225). Whereas, Akkus et al. reported no significance difference in [11C]ABP688 binding in individuals with schizophrenia compared with healthy controls (226), a multi-modal imaging approach, combining mGluR5 PET imaging with [11C]ABP688 together with fMRI reported a lower mGluR5 availability and related functional connectivity alterations in drug-naïve young adults with major depression (227). Esterlis et al. confirmed this hypothesis and objectified an antidepressant response of ketamine through a change in [11C]ABP688 binding that was associated with a significant reduction in depressive symptoms following ketamine administration (228). In alcohol consumption abuse, [11C]ABP688 evidenced altered mGluR5 signaling in the amygdala, that was correlated with the temptation to drink (183).

Regarding the limitations in clinical availability of [11C]ABP688, due to the short physical half-life of carbon-11, fluorinated ABP688 derivatives have been proposed, including the promising radioligand [18F]PSS232. After a preclinical validation evidencing specific and selective *in vitro* and *in vivo* properties (185), Warnock et al. reported recently the first-in-human evaluation of this tracer, highlighting in healthy volunteers a favorable brain uptake pattern and kinetics of [18F]PSS232 (186).

These clinical studies, with sometimes ambiguous or even discordant results, must be put in perspective with regard to the influence of the intrasynaptic concentration in endogenous glutamate on the binding of radioligands. For that purpose, pharmacological challenges have been performed in both preclinical and clinical settings, using several glutamate modulators, including ceftriaxone, a potent GLT-1 activator that decreases extracellular levels of glutamate, N-acetylcysteine (NAC), a promoter of the cysteine–glutamate antiporter that increases extrasynaptic glutamate release, and ketamine, an NMDA glutamate receptor antagonist, that increases glutamate release when administered at subanesthetic doses. To date, these pharmacological explorations remain equivocal according to: 1- the pharmacological compound used; 2- the tested radioligand; 3- the studied species (rodents, non-human primates, or human subjects). Thus, whereas ketamine administration decreases [11C]ABP688 binding *in vivo* in human subjects (229), this result has not been confirmed in rats (230). On the other hand, [18F]PSS232 binding appears to be not impacted to neither acute glutamate shifts after stimulation with N-acetylcysteine (NAC) in human (231) nor ketamine and ceftriaxone infusions in the rat brain (232). This parameter has to be considered carefully to accurately quantify mGluR5 expression *in vivo* using PET.

#### Group II and III

Group II and III mGluRs are mostly located within presynaptic regions and involved in the inhibition of neurotransmitter release. Of all the subtypes, only an mGluR2 tracer has been the subject of a human PET study. [11C]JNJ42491293 is a selective, high-affinity radioligand for the positive allosteric modulator (PAM) site of mGluR2. This site is a potential target for treating anxiety, schizophrenia or addiction. In the first human study, its *in vivo* distribution was consistent with known mGluR2 expression patterns (highest uptake in the striatum and cerebellum) (187). Unfortunately, recent experiments showed an off-target binding *in vivo* and [11C]JNJ42491293 was considered unsuitable for *in vivo* imaging of mGluR2 (233).

## Cholinergic System

The cholinergic system is well known to be involved in cognitive function, and cholinergic dysfunction has been shown to play a key role in the physiopathology of dementia. Targets have been identified by post mortem studies, which have highlighted alterations in functional components of the cholinergic system (234). These include both presynaptic dysfunction [e.g., in acetylcholinesterase (AChE) or vesicular acetylcholine transporters (VAChTs)] and postsynaptic dysfunction [e.g., in nicotinic acetylcholine receptors (nAChR) or muscarinic acetylcholine receptors (mAChR)] (235, 236). Several radiotracers (summarized in Figure 2) have been developed for each of these targets.

There are two PET tracer substrates for AChE: [11C]-PMP and [11C]-MP4A. These have been used in several clinical studies over the past two decades to highlight modifications in AChE activity in patients with AD, PD, PSP or LBD (237–242). [11C]MP4A has a high specificity for AChE, but also a high rate of hydrolysis by this enzyme, and radioligand uptake in regions with high AChE activity is therefore strongly dependent on the rate of transport into the brain (243). By contrast, [11C]PMP exhibits a hydrolysis rate that is three to four times slower than that of [11C]MP4A, allowing for more precise estimates of AChE activity in regions of moderate-to-high AChE concentration (244). Presynaptic cholinergic terminal density can also be assessed with selective radioligands for presynaptic VAChTs. This has been done in clinical studies with [123I]-IBVM (237, 245) and, more recently, in PET imaging with [18F]FEOBV (246). [18F]FEOBV exhibits lower binding in the mesopontine junction and medulla than [123I]IBVM, providing a robust index of VAChT binding (247).

Postsynaptic cholinergic dysfunction has been assessed in patients with AD, using (S)-[11C]nicotine (248–250). However, these [11C]nicotine studies were hindered by high levels of non-specific binding, rapid metabolism, and washout from the brain, as well as a strong dependence on cerebral blood flow (234). New PET and SPECT radioligands have recently been developed to target α4β2 nAChR, which is the most severely affected receptor subtype in AD, with reductions of up to 50% in the neocortex, entorhinal cortex and hippocampus (251). Some clinical studies using either the SPECT tracer [123I]-5IA, or the PET tracer [18F]-2FA, in patients with AD have highlighted significant reductions in α4β2 nAChR in several brain areas, correlated with cognitive impairment (252, 253). Furthermore, another study found a negative correlation between α4β2 nAChR availability and Aβ load (measured by [11C]-PIB), suggesting that Aβ deposition induces the degeneration of cholinergic neurons (254). It was suggested 10 years ago that the α7 nAChR subtype plays a neuroprotective role, by modulating the neurotrophic system that is needed to maintain cholinergic neuron integrity, and by stimulating signal transduction pathways that support neuron survival. In AD, α7 nAChR is implicated in Aβ toxicity and tau phosphorylation (255). Moreover, deletion of the α7 nAChR gene has been shown to reduce cognitive impairment in animal models of AD (256). Further PET studies using radioligands specific to the α7 nAChR, such as [18F]ASEM, are needed to determine the relationship between α7 nAChR and AD pathology (234).

In PD, LBD or PSP, mAChR has also been imaged with [123I]QNB and [11C]NMPB (257, 258), which are high-affinity mAChR antagonists with similar chemical structures and regional brain distributions. These radiotracers are able to penetrate the BBB efficiently, but non-specifically in relation to the mAChR subtype (234).

**Figure 5:**
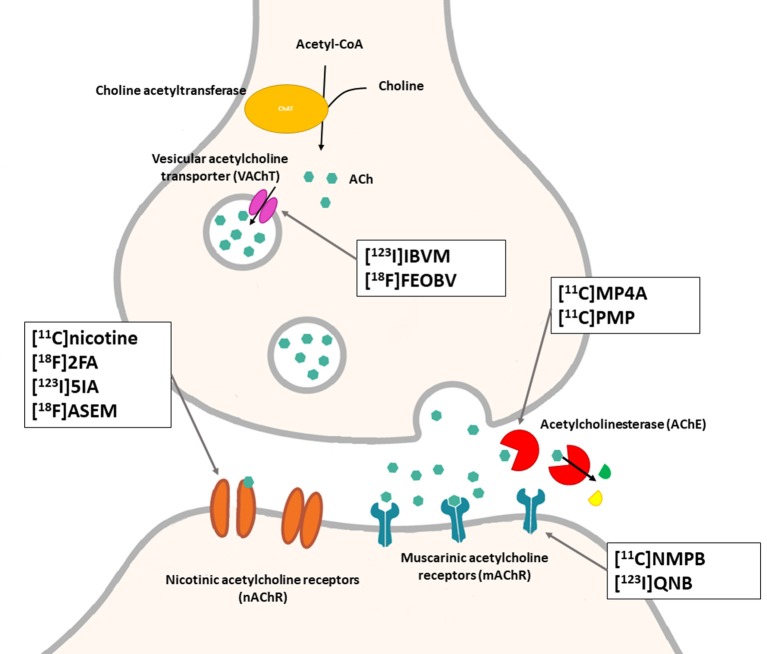
Schematic illustration of the main cholinergic PET and SPECT radioligands, and their presynaptic or postsynaptic targets. Acetylcholine (ACh) is synthesized by choline acetyltransferase from choline and acetylCoA. ACh is released into the synaptic cleft, where it can bind to two types of receptors expressed on postsynaptic neurons: nicotinic receptors (nAChR) and muscarinic receptors (mAChR). ACh is degraded to choline and acetate by acetylcholinesterase (AChE). The reuptake of choline into presynaptic neurons occurs via a choline transporter. Choline is recycled within presynaptic neurons to form ACh, and stored in vesicles by a presynaptic vesicular ACh transporter (VAChT).

All these cholinergic tracers are resumed in Figure 5 and Table 6.

**Table 6:** Main SPECT and PET cholinergic tracers, molecular structures, pharmacological properties, and examples of clinical studies.

**Compounds**	**Imaging modality**	**Target/ measure**	**Affinity (nM)**	**Clinical studies**	**Compounds**	**Imaging modality**	**Target/ measure**	**Affinity (nM)**	**Clinical studies**
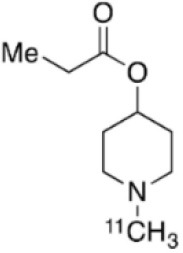 [^11^C]-PMP	TEP	AChE activity	NA	AD (237, 239, 240, 259), PD (239)	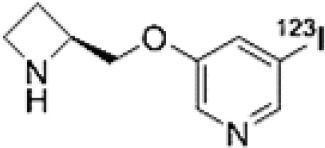 [^123^I]-5IA	SPECT	α4β2 nAChR density	K_d_ = 0.011 in rats (260)	AD (252)
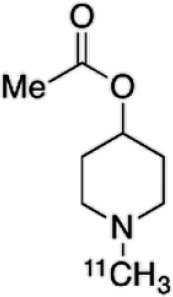 [^11^C]-MP4A	TEP	AChE activity	NA	AD (242), PD (238, 241), LBD (261), PSP (238)	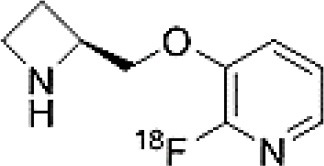 [^18^F]-2FA	TEP	α4β2 nAChR density	K_i_ = 0.046 in rats (262)	AD (253)
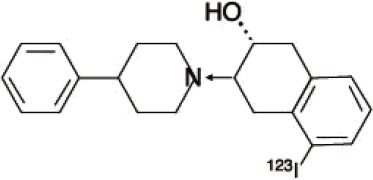 [^123^I]-IBVM	SPECT	VAChT density	IC_50_ = 2.5 ± 0.2 in rats (263)	MSA (245)	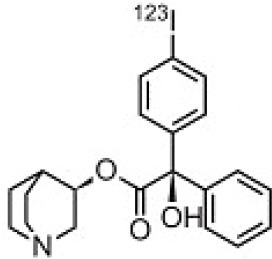 [^123^I]-QNB	SPECT	mAChR density	IC_50_ = 0.8 in mouse (264)	AD (265), PD, LBD (258)
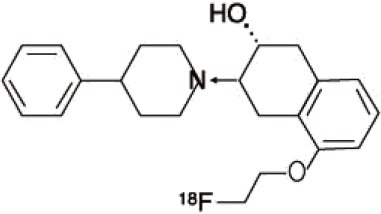 [^18^F]-FEOBV	TEP	VAChT density		AD (266), LBD (246)	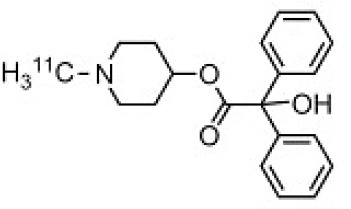 [^11^C]-NMPB	TEP	mAChR density	IC_50_ = 1.8 in mouse (264)	AD (267), PD, PSP (257)
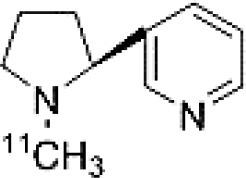 [^11^C]-nicotine	TEP	α4β2 nAChR density	K_d_ = 2.4 in rats (268)	AD (248–250)	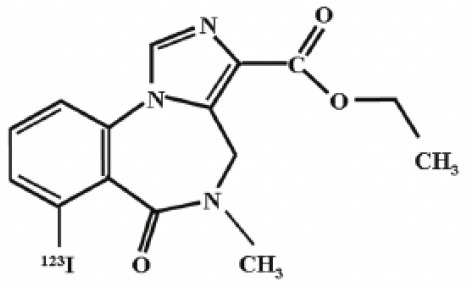 [^18^F]-ASEM	TEP	α7 nAChR density	K_i_ = 0.3 in HEK293 cells stably transfected with rat α7 nAChR (269)	Schizophrenia (270)

## GABA_A_ Receptors

γ-Amino butyric acid (GABA), is the predominant inhibitory neurotransmitter in the central nervous system. This neurotransmitter is able to bind to two types of receptor: ionotropic GABAA/C and metabotropic GABAB. GABAA receptors, also known as the central benzodiazepine receptor, are found on most neurons in the brain, and are part of a superfamily of ligand-gated ion channels. They have a primary binding site for GABA, as well as multiple allosteric modulatory sites. When benzodiazepines, or other allosteric modulators such as barbiturates, bind to GABAA receptors, conformational changes increase the permeability of the central pore to chloride ions, resulting in a chloride flux that hyperpolarizes the neuron (271). GABAA receptors can be composed of several subunit isoforms (272), but only pentamers containing α1, α2, α3, α4, or α5 subunits are benzodiazepine sensitive. These various subunits have a region-specific distribution in the brain, and are believed to subserve different functional and physiological roles and mediate a variety of pharmacological effects. Impairment of GABAA receptor function is increasingly recognized to play a major role in the pathophysiology of several neuropsychiatric diseases such as AD, epilepsy, panic disorders, major depression, cortical brain damage following an acute stroke, anxiety disorders, and chronic alcohol dependency (273). Radiotracers that bind to benzodiazepine sites on GABAA receptors (GABAA-BZ sites) have been shown to be useful for investigating these disorders (274). The first molecules developed for GABAA receptor imaging was carbon-11 labeled benzodiazepines such as [11C]flunitrazepam, [11C]diazepam, or [11C]fludiazepam, but the lack of specificity and *in vivo* affinity of these ligands (Kd ≥ 10 nM) did not allow accurate determination of GABAA receptor density (275). The triazolobenzodiazepine [11C]alprazolam have also been investigated. Despite an increased affinity (Kd = 3.4 nM), PET studies in six healthy volunteers showed a low extraction into brain (<1% of injected dose), and a substantial depot effect probably into the lungs (276). Finally, the imidazobenzodiazepine flumazenil (Ro 15-1788 or N-methyl-11C]flumazenil), became the most commonly used radioligand for GABAA receptor imagingand is still extensively used to quantify benzodiazepine binding in the human brain (277–279). It was used to measure changes in GABA levels (280), as well as to quantify BZ receptors density in the epileptic foci of patients with partial epilepsy (281–283), in schizophrenic patients (284), neuronal loss in stroke (285), and more recently as a tool in clinical research to evaluate GABAA receptor occupancy using molecules with potential anxiolytic properties (286). [123I]iomazenil, a iodo-analog of flumazenil with very similar binding profile, has also been widely used in clinical studies (287–289).

[11C]Ro15-4513 is a partial inverse agonist at the GABAA-BZ site, preferentially targeting α5 subunits (290, 291). Like the previous ones, this tracer has also been used in clinical studies to understand the precise involvement of GABAA receptors in different neuropsychiatric diseases and the relationship between GABAA receptor density and clinical symptoms (292, 293).

Several attempts of fluorine-18 labeling of flumazenil were performed. Thus, [18F]-FEF, [18F]-FFMZ, and [18F]-flumazenil have been tested. Studies have demonstrated the superiority of [18F]-flumazenil because of a higher affinity and lower levels of radiometabolites in brain (275, 294). Because of the longer half-life of the isotope, this tracer could become the “gold standard” in benzodiazepine PET studies.

The development of GABAA radioligands (summarized in Table 7) need several improvements. Several improvements are needed. First, is to develop receptor subtype specific radioligands such as [11C]Ro15-4513. Radioligands specific for all the GABAA receptor subtypes would be of great importance to PET imaging. The second important enhancement is to develop and apply full agonist radioligands sensitive to changes in endogenous neurotransmitter levels. Finally, development of radiotracers specific to other sites than the BZ binding site will be important in order to further investigate GABAA pharmacology as well as to investigate the role of GABAA receptors in various disease staFinally, development of radiotracers specific to other sites than the BZ binding site will be important in order to further investigate GABAA pharmacology as well as to investigate the role of GABAA receptors in various disease states (275).

**Table 7:** Main radioligands for GABAA receptors imaging, molecular structures, pharmacological properties, and examples of clinical studies.

**Compounds**	**Imaging modality**	**Target/ measure**	**Affinity (nM)**	**Clinical trials**
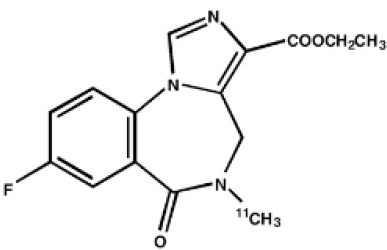 [^11^C]-FMZ	PET	GABA_A_-BZ sites (α1, α2, α3, and α5 subunits)	K_i_ ≈ 1.3 (BZRs containing α1, α2, α3, or α5 subunits) K_i_ ≈ 150 (BZRs containing α4, or α6 subunits) (295)	Epilepsy (281–283) Stroke (285) Schizophrenia (284)
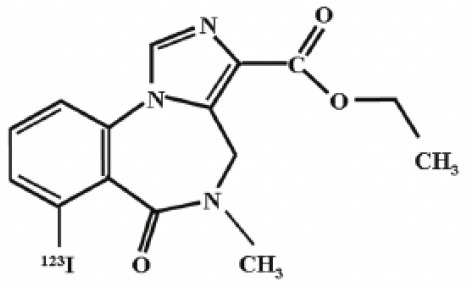 [^123^I]-IMZ	SPECT	GABA_A_-BZ sites	K_i_ = 0.47 (in primates) (296)	Stroke (287) Epilepsy (288) Anorexia nervosa (289)
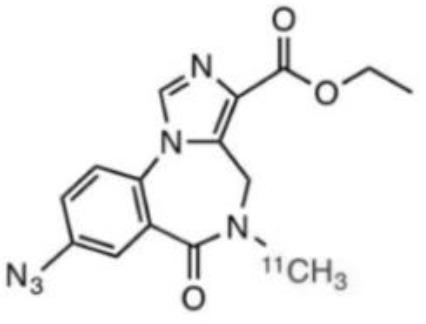 [^11^C]Ro15-4513	PET	GABA_A_-BZ sites α5 subtype	K_i_ = 0.3 (BZRs containing α5 subunits) (290)	Alcohol dependence (292) Schizophrenia (293) Autism (297)
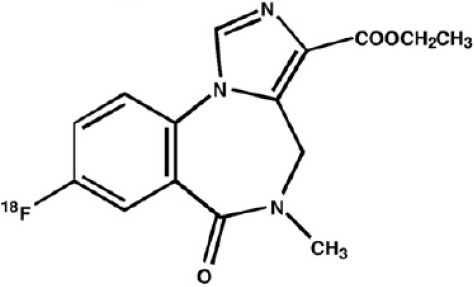 [^18^F]-flumazenil	PET	GABA_A_-BZ sites	–	Epilepsy (298, 299)

## Serotoninergic System

The serotonergic system plays an important modulatory role in many central nervous system functions. It is the target of many drugs commonly used to treat brain disorders, either through reuptake blockade or via interactions with serotonin (5-HT) receptors. Serotonergic dysfunction has been involved in the etiology of many psychiatric disorders, including depression, anxiety and schizophrenia, as well as neurological diseases such as AD and epilepsy. Currently available radiotracers for *in vivo* brain imaging of the 5-HT system in humans include radioligands for the 5-HT1A, 5-HT1B, 5-HT2A and 5-HT4 receptors, and for the 5-HT transporter (SERT) (300).

The 5-HT1A receptor is one of the most extensively studied receptors in the serotonergic family. Like most 5-HT receptors, it is a G protein-coupled receptor (GPCR) with seven membrane-spanning domains. It serves as an inhibitory autoreceptor in the raphe nuclei, and is targeted by serotonin reuptake inhibitors. It also plays a role with 5-HT4 and 5-HT6 receptors in learning and memory (301, 302). Several radioligands have been synthesized up to now, but only three are in frequent use in clinical studies. The two most widely used are [carbonyl-11C]WAY-100635 and [18F]MPPF (300). These two radioligands are selective and high-affinity 5-HT1A receptor antagonists with a high target-to-background ratio. These tracers have been used in numerous studies of patients with psychiatric disorders such as panic disorder (303), bipolar depression (218) and anorexia nervosa (304), as well as in neurological disorders such as epilepsy, cognitive impairment, AD and migraine (305–311). The third 5-HT1A antagonist radioligand used in clinical studies is [18F]-FCWAY (312), a fluorinated analog of WAY-100635, which also has high 5-HT1A affinity and a high hippocampal-to-cerebellar binding ratio (313–317). However, this compound undergoes high defluorination *in vivo*, leading to high bone radioactivity uptake. Although this radiodefluorination has been prevented in humans by preadministering disulfiram, this drawback may explain why its use has not been expanded beyond a single PET center (300). A novel and promising 18F-labeled radiotracer, [18F]MefWAY, that is thought to be resistant to defluorination *in vivo* was recently administered to healthy humans, but no clinical study has yet been published (318). There has been recent interest in the use of 5-HT1A agonists to study variations in endogenous 5-HT levels. [11C]CUMI-101 shows high affinity, but its sensitivity to endogenous 5-HT variations *in vivo* has not yet been reported (319).

Because they are involved in the etiology and treatment of many psychiatric disorders, 5-HT2A receptors have also been imaged. Five specific radioligands of this receptor have successfully been used in clinical studies: [123I]-R91150, and the PET radioligands [18F]setoperone, [18F]altanserin, [18F]deuteroaltanserin, and [11C]MDL 100, 907. Despite its low signal-to-noise ratio, [123I]-R91150 has often been used in drug occupancy studies, on account of the widespread availability of SPECT (320, 321). It has also been used to study changes in 5-HT2A receptor density that are implicated in various diseases, including cognitive decline (322), suicidal behavior (323), and anorexia nervosa (324). [18F]altanserin is the most frequently used PET tracer. Although it is metabolized to lipophilic radiometabolites, which contribute to non-specific binding, like the previous one, this tracer has been used to determine 5-HT2A receptor density in relation to several psychiatric diseases, such as depression (325), cognitive decline (326), Tourette's syndrome (327), schizophrenia (328) and other neuropsychiatric disorders (329, 330).

Another target allowing for serotoninergic system imaging is the SERT. Interest in SERT imaging has been stimulated by the success of serotonin reuptake inhibitors. The three most widely used belong to the diarylsulfide family: [11C]-DASB, [11C]-MADAM, [123I]-ADAM (300). These radiotracers have been successfully used to estimate SERT occupancy by selective serotonin reuptake inhibitors (331–337), in order to demonstrate changes in SERT density in several neuropsychiatric disorders and throughout their treatment (338–344), as well as in healthy individuals to investigate physiological variations such as personality traits (345) or seasonal changes (346). Other specific radiotracers for this target are still being developed: 4-[18F]ADAM has yielded promising results (347, 348).

All these serotoninergic tracers are summarized in Table 8.

**Table 8:** Main serotoninergic radioligands, molecular structures, pharmacological properties, and examples of clinical studies.

**Compounds**	**Imaging modality**	**Target/ measure**	**Affinity (nM)**	**Clinical studies**	**Compounds**	**Imaging modality**	**Target/ measure**	**Affinity (nM)**	**Clinical studies**
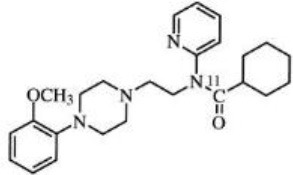 [^11^C]-WAY-100635	PET	5-HT_1A_ density (antagonist)	K_d_ = 0.2–0.4 (300)	PD (306), depression (349), panic disorder (303), social anxiety disorder (302), anorexia nervosa (304)	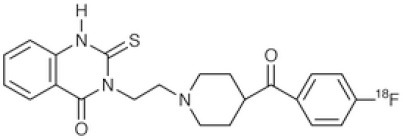 [^18^F]altanserin	PET	5-HT_2A_ density	K_i_ = 0.13 (300)	AD (326), depression (325), schizophrenia (328), Tourette's syndrome (327), anorexia nervosa (329), obsessive compulsive disorder (330)
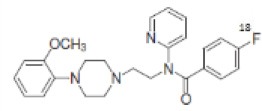 [^18^F]MPPF	PET	5-HT_1A_ density (antagonist)	K_d_ = 0.3 (300)	AD (308), epilepsy (282, 307, 311), migraine (309, 310)	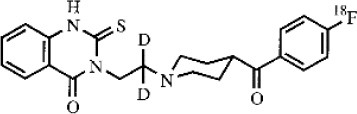 [^18^F]deuteroaltanserin	PET	5-HT_2A_ density	–	AD (350)
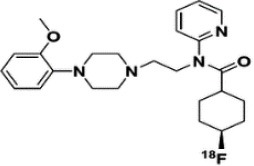 [^18^F]FCWAY	PET	5-HT_1A_ density (antagonist)	K_i_ = 0.25 (300)	Epilepsy (313–315), panic disorder (316)	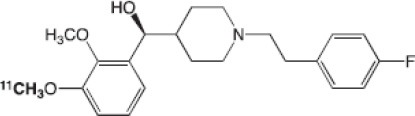 [^11^C]MDL-100,907	PET	5-HT_2A_ density	K_d_ = 0.14–0.19 (300)	Depression (351), obsessive compulsive disorder (352)
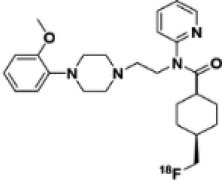 [^18^F]MefWAY	PET	5-HT_1A_ density (antagonist)	IC_50_ = 26 in rats (353)	–	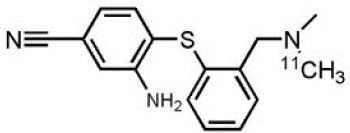 [^11^C]DASB	PET	SERT density	Ki = 0.97 ± 0.07(354)	Depression (340), schizophrenia (341), alcohol dependence (343), obsessive compulsive disorder (342), bipolar disorder (344)
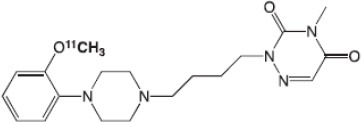 [^11^C]CUMI-101	PET	5-HT_1A_ density (partial agonist)	K_i_ = 0.15 (300)	Measure of endogenous changes in serotonergic neurotransmission (355)	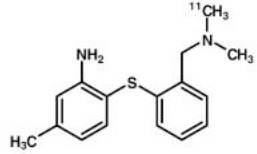 [^11^C]MADAM	PET	SERT density	Kd = 0.02 (356)^*^	–
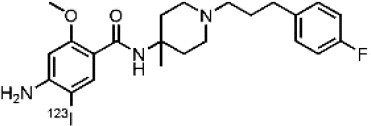 [^123^I]R91150	SPECT	5-HT_2A_ density	K_d_ = 0.11 (300)	AD (322), anorexia nervosa (324), suicidal behavior (323)	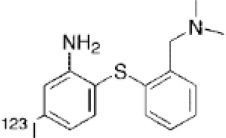 [^123^I]ADAM	SPECT	SERT density	Kd = 0.03 (356)	Depression (338), migraine (339)
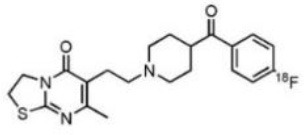 [^18^F]setoperone	PET	5-HT_2A_ density	K_d_ = 0.7 in rats (357)	AD (358), migraine (359), stroke (360), depression (361)	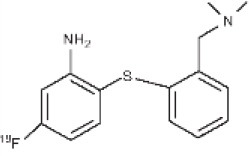 4-[^18^F]ADAM	PET	SERT density	K_i_ = 0.081 (362)	Depression (348)

* *Determined for [3H]MADAM*.

## α-Synuclein

α-synuclein (α-Syn) is a phosphoprotein found in Lewy bodies (LBs), pathological inclusions that are the hallmark of PD and LBD, as well as in the glial cytoplasmic inclusions (GCIs) that are typical of MSA. All these diseases fall now under the heading of synucleinopathies (363). α-Syn aggregates might induce mitochondrial and proteasomal dysfunction, and interfere with vesicular trafficking within dopamine neurons, leading to their degeneration (364). These protein aggregates have been shown to spread from cell to cell via the extracellular space, and the presence of α-Syn has been demonstrated in extracellular matrices such as plasma, conditioned cell media, and cerebrospinal fluid (365, 366). It is thought that occult α-Syn deposition may occur years before the onset of motor symptoms. Hence, accurate and early detection of premotor synucleinopathies may benefit more from α-Syn imaging, rather than from evidence of dopaminergic changes (367, 368). Although several molecules are able to bind to aggregated α-Syn, a selective imaging biomarker has not been found yet. A sensitive and specific α-Syn radiotracer would have to fulfill several criteria. First, α-Syn exist in different forms, including soluble and insoluble oligomers. An imbalance between these two species led to the formation of pathologic aggregates (369, 370), which have to be recognized by the tracer. Secondly, α-Syn aggregates have distinct cellular localization patterns according to the synucleinopathy, with intraneuronal aggregates (e.g., LBs) in PD, and oligodendrocytic aggregates (e.g., GCIs) in MSA. The ideal α-Syn radiotracer would be able to detect and differentiate these different locations, thereby providing a potential tool for differential diagnosis. Third, colocalization between α-Syn aggregates and other aggregating proteins, such as tau and Aβ (371), has frequently been reported. The optimum tracer would have to be able to specifically detect α-Syn with regard to other deposits, despite their small size and low density. Finally, α-Syn undergoes various posttranslational modifications, such as oxidative modification (372), phosphorylation (373, 374), and N-terminal acetylation, all of which the tracer should be able to detect (363).

As explained above, several molecules are able to cross the BBB and bind to aggregated α-Syn. Unfortunately, these molecules also tend to bind to other aggregated proteins, including Aβ plaques. In this context, diverse Aβ-binding compounds have been investigated for potential affinity for α-Syn, such as [11C]-PIB (375), and more especially [18F]-BF227. *In vitro* binding studies indicate that [18F]-BF227 binds with high affinity to two binding sites on Aβ1–42 fibrils, and to one class of binding site on α-Syn fibrils. [18F]-BF227 has been found to bind to Aβ-containing AD brain, but failed to bind to Aβ-free LBD or age-matched control homogenates. Furthermore, [18F]-BF227 labeled both Aβ plaques and LBs in an immunohistochemical/fluorescence analysis of human AD and PD brain sections (376). [18F]-BF227 has also been reported to stain GCIs in post mortem tissues, and [11C]-BF227 PET was used to measure the aggregated α-Syn load in eight cases of probable MSA (377). This study demonstrated high signals in GCI-rich brain regions, including subcortical white matter and the putamen, globus pallidus, primary motor cortex, and anterior and posterior cingulate cortex. However, a very recent autoradiography study failed to support binding of [18F]BF-227 to CGI at concentrations typically achieved in PET experiments (378). The lack of specificity and affinity of [18F]-BF227 means that it cannot be used to diagnose synucleinopathies, although it could, theoretically, still be used to monitor changes in α-Syn aggregate load after interventions such as immunotherapy. Levels of other aggregated proteins, such as Aβ, would first have to be independently determined (368).

The last reported α-Syn radioligand is [125I]-SIL23 (379). This tracer has been found to bind to α-Syn fibrils in post mortem brain tissue from patients with PD, as well as to α-Syn in a transgenic mouse model for PD. However, the affinity of SIL23 for α-Syn vs. Aβ and tau fibrils is not optimum for imaging fibrillar α-Syn *in vivo*. Moreover, high non-specific binding, including non-specific binding in white matter liable to be secondary to lipophilic interactions, also appeared to limit autoradiography with SIL23 in preliminary experiments.

To conclude, the development of an α-Syn PET radiotracer is particularly challenging, and although several studies have tried to develop suitable PET α-Syn radiotracers (380), the ideal candidate remains elusive. These three radiotracers and their main properties are resumed in Table 9.

**Table 9:** Main PET and SPECT radiotracers relevant to α-Syn imaging, molecular structures, pharmacological properties, and examples of clinical trials. ^*^determined for [3H]-PIB.

**Compounds**	**Imaging modality**	**Affinity for α-Syn fibrils (nM)**	**Affinity for Aβ fibrils (nM)**	**Clinical trials**
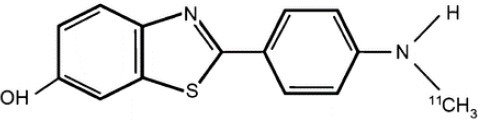 [^11^C]-PIB	PET	K_d_ = 4.16^*^ (381)	K_d1_ = 0.71^*^ K_d2_ = 19.80^*^ (Aβ_1−42_ fibrils) (382)	Not used in clinical trials for α-Syn imaging
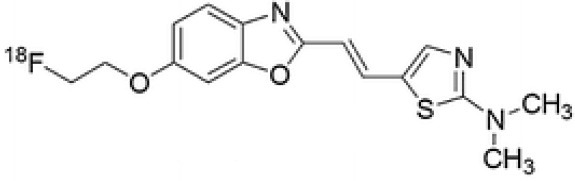 [^18^F]-BF227	PET	K_d_ = 14.03 ± 43.52 (380)	K_d1_ = 0.82 ± 1.08 K_d2_ = 125.2 ± 29.05 (Aβ_42_ fibrils) (380)	MSA (377)
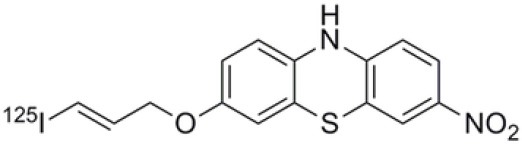 [^125^I]-SIL23	SPECT	K_d_ = 148 (379)	K_d_ = 635 (379)	–

## Discussion

Molecular imaging agents have evolved from non-specific agents to ligands with very high selectivity for specific brain targets such as receptors, neurotransmitter transporters, or abnormal protein deposits over the last decades. Through the nine targets mentioned above, we have seen that the specificity of the ligands for their target is of paramount importance. Indeed, cross binding affinities of several radioligands could reduce the specificity of the results and may interfere with diagnosis.

More and more the diagnosis of dopaminergic disorders is sustained by molecular imaging combined with clinical examination and have been included in guidelines (383, 384). Thus, molecular imaging is used as an ancillary tool when clinical symptoms are insufficient to confirm a diagnosis. Dopaminergic imaging rests on F-DOPA, but mostly on DAT imaging (especially [123I]-FPCIT), which is considered more relevant to evaluate dopaminergic neuron loss. Thus, LBT-999 could be of great interest in the future because of its better sensitivity, and the higher resolution of PET imaging. In parallel, the increase in attempted to graft dopaminergic neurons may drive up F-DOPA imaging to monitor cell survival. An interesting target remain particularly challenging: indeed, to date, α-Syn cannot be specifically detected with existing radiotracers. This target constituting the hallmark of PD, LBD and MSA, its early visualization could be considerably helpful for diagnosis.

In regards to AD imaging, the first investigations was the assessment of cerebral perfusion. Then, [18F]FDG has allowed to assess cerebral glucose metabolism, and remains a widely prescribed exam at present. Within the last decades, amyloid imaging became the most specific examination because of its excellent negative predictive value, and allow therapeutic stratification in clinical trials. In 2007 (later updated in 2010), Dubois and al. published revised criteria for AD that for the first time included AD biomarkers (amyloid PET and CSF Aβ42) as a supportive criteria. However, Aβ plaques are not correlated with cognitive decline, therefore, clinical research is increasingly turning to tau and neuroinflammation imaging to assess new treatments and follow-up disease progression. Further radiotracers targeting other mechanisms, such as [18F]FNM or [18F]-2FA, could be used in AD studies to improve understanding of the cascades of events leading to neurodegeneration.

Psychiatric diseases diagnosis does not call for molecular imaging in clinical routine. However, in psychiatry, physiopathological modifications behind the symptoms remain not well known and understood. Hence, PET and SPECT radioligands such as, serotonergic, GABAergic or glutamatergic tracers, are a powerful tool to improve psychiatric nosography. Nowadays, it is possible to quantify receptors and transporters imbalances in numerous psychiatric diseases including depression, anxiety and schizophrenia, and explore different treatments options. Moreover, several hypothesis suggest a potential link between excitotoxicity and psychiatrics disorders especially schizophrenia. The hypothesis suggest that progressive excitotoxic neural cell death in hippocampal and cortical areas occurs via “disinhibition” of glutamatergic projection to these areas. Disinhibited glutamatergic activity could result from inhibition of glutamate-mediated neurotransmission and a consequent failure to stimulate inhibitory GABAergic neurons, and/or degeneration of inhibitory GABAergic interneurons (385). Unfortunately, too few studies have been performed yet to highlight this hypothesis. Today, more tracers are be needed to explore glutamatergic and GABAergic systems.

## Conclusion

After several decades of research, some radiotracers targeting a hallmark of a disease are valuable diagnostic tools in clinical routine and research, and are used on a large scale. Recently, numerous radiotracers have been developed in order to detect primary changes in brain tissue, and improve our understanding of physiopathological mechanisms of neuropsychiatric diseases. These radioligands provide quantitative and topographical information on the evolution of their target during the course of the disease. More than diagnostic tools, they are one of the only ways to better understand the functioning of the brain in the healthy man and in pathological conditions. Their future usefulness is more focused on therapy monitoring than on the diagnosis itself. As in oncology, molecular neuroimaging is now becoming a therapeutic assistance tool, for screening patient's eligibility for drugs and monitoring the proper functioning of therapy. These new companion drugs are a new challenge for molecular imaging, and quantitative and kinetic analyzes seem to be increasingly relevant for image interpretation. Further development in understanding radiotracer metabolization, binding characteristics, BBB crossing, and clinicopathologic correlations of all these imaging probes will assert their clinical utility, and will lead to the development of more neuroimaging probes in the future.

## Author Contributions

This review was written by MB, A-SS and NA. Correction was made by MR, PD, FL, J-FD, and PP. PP was also involved in the plan development.

### Conflict of Interest

The authors declare that the research was conducted in the absence of any commercial or financial relationships that could be construed as a potential conflict of interest.

## References

[1] Mankoff DA. A definition of molecular imaging. J Nucl Med Off Publ Soc Nucl Med. (2007) 48:18N, 21N. 17536102

[2] Pysz MA Gambhir SS Willmann JK. Molecular imaging: current status and emerging strategies. Clin Radiol. (2010) 65:500–16. 10.1016/j.crad.2010.03.011 20541650 PMC3150531

[3] O'Brien TJ So EL Mullan BP Hauser MF Brinkmann BH Bohnen NI. Subtraction ictal SPECT co-registered to MRI improves clinical usefulness of SPECT in localizing the surgical seizure focus. Neurology. (1998) 50:445–54. 10.1212/WNL.50.2.445 9484370

[4] Varrone A Asenbaum S Vander Borght T Booij J Nobili F Någren K EANM procedure guidelines for PET brain imaging using [18F]FDG, version 2. Eur J Nucl Med Mol Imaging. (2009) 36:2103–10. 10.1007/s00259-009-1264-0 19838705

[5] Goldman JG Holden SK Litvan I McKeith I Stebbins GT Taylor J-P. Evolution of diagnostic criteria and assessments for Parkinson's disease mild cognitive impairment. Mov Disord Off J Mov Disord Soc. (2018) 33:503–10. 10.1002/mds.27323 29488270 PMC12570294

[6] Dubois B Hampel H Feldman HH Scheltens P Aisen P Andrieu S. Preclinical Alzheimer's disease: definition, natural history, and diagnostic criteria. Alzheimers Dement. (2016) 12:292–323. 10.1016/j.jalz.2016.02.002 27012484 PMC6417794

[7] Finnema SJ Scheinin M Shahid M Lehto J Borroni E Bang-Andersen B. Application of cross-species PET imaging to assess neurotransmitter release in brain. Psychopharmacology. (2015) 232:4129–57. 10.1007/s00213-015-3938-6 25921033 PMC4600473

[8] Baulieu J-L Le-Pogam A Leborgne A Guilloteau D Prunier-Aesch C Imagerie moléculaire de la maladie de Parkinson : données actuelles. Médecine Nucl. (2008) 32:236–41. 10.1016/j.mednuc.2008.02.001

[9] Garnett ES Firnau G Nahmias C. Dopamine visualized in the basal ganglia of living man. Nature. (1983) 305:137–8. 10.1038/305137a0 6604227

[10] Becker G Müller A Braune S Büttner T Benecke R Greulich W. Early diagnosis of Parkinson's disease. J Neurol. (2002) 249:iii40–8. 10.1007/s00415-002-1309-9 12522572

[11] Snow BJ Tooyama I McGeer EG Yamada T Calne DB Takahashi H. Human positron emission tomographic [18F]fluorodopa studies correlate with dopamine cell counts and levels. Ann Neurol. (1993) 34:324–30. 10.1002/ana.410340304 8363349

[12] Ribeiro MJ Remy P Bendriem B Almeida P Brulon V Samson Y. Comparison of clinical data sets acquired on different tomographs using 6-18F-L-dopa. Eur J Nucl Med. (2000) 27:707–12. 10.1007/s002590050566 10901458

[13] Ma Y Tang C Chaly T Greene P Breeze R Fahn S. Dopamine cell implantation in Parkinson's disease: long-term clinical and (18)F-FDOPA PET outcomes. J Nucl Med Off Publ Soc Nucl Med. (2010) 51:7–15. 10.2967/jnumed.109.066811 20008998 PMC2946843

[14] Akamatsu G Ohnishi A Aita K Nishida H Ikari Y Sasaki M. A revisit to quantitative PET with 18F-FDOPA of high specific activity using a high-resolution condition in view of application to regenerative therapy. Ann Nucl Med. (2017) 31:163–71. 10.1007/s12149-016-1143-2 27914043

[15] Ziebell M Holm-Hansen S Thomsen G Wagner A Jensen P Pinborg LH. Serotonin transporters in dopamine transporter imaging: a head-to-head comparison of dopamine transporter SPECT radioligands 123I-FP-CIT and 123I-PE2I. J Nucl Med Off Publ Soc Nucl Med. (2010) 51:1885–91. 10.2967/jnumed.110.078337 21078806

[16] Ito Y Fujita M Shimada S Watanabe Y Okada T Kusuoka H. Comparison between the decrease of dopamine transporter and that of L-DOPA uptake for detection of early to advanced stage of Parkinson's disease in animal models. Synap N Y N. (1999) 31:178–85. 10.1002/(SICI)1098-2396(19990301)31:3<178::AID-SYN2>3.0.CO;2-M 10029235

[17] Eshuis SA Maguire RP Leenders KL Jonkman S Jager PL. Comparison of FP-CIT SPECT with F-DOPA PET in patients with *de novo* and advanced Parkinson's disease. Eur J Nucl Med Mol Imaging. (2006) 33:200–9. 10.1007/s00259-005-1904-y 16228235

[18] McKeith I O'Brien J Walker Z Tatsch K Booij J Darcourt J. Sensitivity and specificity of dopamine transporter imaging with 123I-FP-CIT SPECT in dementia with Lewy bodies: a phase III, multicentre study. Lancet Neurol. (2007) 6:305–13. 10.1016/S1474-4422(07)70057-1 17362834

[19] Mateos JJ Lomeña F Parellada E Mireia F Fernandez-Egea E Pavia J Lower striatal dopamine transporter binding in neuroleptic-naive schizophrenic patients is not related to antipsychotic treatment but it suggests an illness trait. Psychopharmacology. (2007) 191:805–11. 10.1007/s00213-006-0570-5 17019564

[20] Lehto SM Tolmunen T Joensuu M Saarinen PI Valkonen-Korhonen M Vanninen R. Changes in midbrain serotonin transporter availability in atypically depressed subjects after one year of psychotherapy. Prog Neuropsychopharmacol Biol Psychiatry. (2008) 32:229–37. 10.1016/j.pnpbp.2007.08.013 17884269

[21] Huang W-S Chiang Y-H Lin J-C Chou Y-H Cheng C-Y Liu R-S. Crossover study of (99m)Tc-TRODAT-1 SPECT and (18)F-FDOPA PET in Parkinson's disease patients. J Nucl Med Off Publ Soc Nucl Med. (2003) 44:999–1005. 12843212

[22] Mozley PD Schneider JS Acton PD Plössl K Stern MB Siderowf A Binding of [99mTc]TRODAT-1 to dopamine transporters in patients with Parkinson's disease and in healthy volunteers. J Nucl Med Off Publ Soc Nucl Med. (2000) 41:584–9. 10768556

[23] Kung MP Stevenson DA Plössl K Meegalla SK Beckwith A Essman WD. [99mTc]TRODAT-1: a novel technetium-99m complex as a dopamine transporter imaging agent. Eur J Nucl Med. (1997) 24:372–80. 10.1007/BF00881808 9096087

[24] Huang WS Lin SZ Lin JC Wey SP Ting G Liu RS. Evaluation of early-stage Parkinson's disease with 99mTc-TRODAT-1 imaging. J Nucl Med Off Publ Soc Nucl Med. (2001) 42:1303–8. 11535717

[25] Emond P Guilloteau D Chalon S. PE2I: a radiopharmaceutical for *in vivo* exploration of the dopamine transporter. CNS Neurosci Ther. (2008) 14:47–64. 10.1111/j.1755-5949.2007.00033.x 18482099 PMC6494010

[26] Chalon S Hall H Saba W Garreau L Dollé F Halldin C. Pharmacological characterization of (E)-N-(4-fluorobut-2-enyl)-2beta-carbomethoxy-3beta-(4'-tolyl)nortropane (LBT-999) as a highly promising fluorinated ligand for the dopamine transporter. J Pharmacol Exp Ther. (2006) 317:147–52. 10.1124/jpet.105.096792 16339913

[27] Dollé F Emond P Mavel S Demphel S Hinnen F Mincheva Z. Synthesis, radiosynthesis and *in vivo* preliminary evaluation of [11C]LBT-999, a selective radioligand for the visualisation of the dopamine transporter with PET. Bioorg Med Chem. (2006) 14:1115–25. 10.1016/j.bmc.2005.09.035 16219467

[28] Saba W Valette H Schöllhorn-Peyronneau M-A Coulon C Ottaviani M Chalon S. [11C]LBT-999: a suitable radioligand for investigation of extra-striatal dopamine transporter with PET. Synap N Y N. (2007) 61:17–23. 10.1002/syn.20337 17068778

[29] Varrone A Stepanov V Nakao R Tóth M Gulyás B Emond P. Imaging of the striatal and extrastriatal dopamine transporter with (18)F-LBT-999: quantification, biodistribution, and radiation dosimetry in nonhuman primates. J Nucl Med Off Publ Soc Nucl Med. (2011) 52:1313–21. 10.2967/jnumed.111.089953 21764797

[30] Brooks DJ. Technology insight: imaging neurodegeneration in Parkinson's disease. Nat Clin Pract Neurol. (2008) 4:267–77. 10.1038/ncpneuro0773 18382437

[31] Ghadery C Strafella AP. New imaging markers for movement disorders. Curr Neurol Neurosci Rep. (2018) 18:22. 10.1007/s11910-018-0830-x 29616343

[32] Fuente-Fernández R de la Sossi V McCormick S Schulzer M Ruth TJ Stoessl AJ Visualizing vesicular dopamine dynamics in Parkinson's disease. Synapse. (2009) 63:713–6. 10.1002/syn.20653 19391152

[33] Plotkin M Amthauer H Klaffke S Kühn A Lüdemann L Arnold G. Combined 123I-FP-CIT and 123I-IBZM SPECT for the diagnosis of parkinsonian syndromes: study on 72 patients. J Neural Transm. (2005) 112:677–92. 10.1007/s00702-004-0208-x 15375677

[34] Knudsen GM Karlsborg M Thomsen G Krabbe K Regeur L Nygaard T. Imaging of dopamine transporters and D2 receptors in patients with Parkinson's disease and multiple system atrophy. Eur J Nucl Med Mol Imaging. (2004) 31:1631–8. 10.1007/s00259-004-1578-x 15583914

[35] Orimo S Suzuki M Inaba A Mizusawa H. 123I-MIBG myocardial scintigraphy for differentiating Parkinson's disease from other neurodegenerative parkinsonism: a systematic review and meta-analysis. Parkinsonism Relat Disord. (2012) 18:494–500. 10.1016/j.parkreldis.2012.01.009 22321865

[36] Volkow ND Fowler JS Wang GJ Baler R Telang F. Imaging dopamine's role in drug abuse and addiction. Neuropharmacology. (2009) 56(Suppl. 1):3–8. 10.1016/j.neuropharm.2008.05.022 18617195 PMC2696819

[37] Shen L-H Liao M-H Tseng Y-C. Recent advances in imaging of dopaminergic neurons for evaluation of neuropsychiatric disorders. J Biomed Biotechnol. (2012) 2012:259349. 10.1155/2012/259349 22570524 PMC3335602

[38] Klein JC Eggers C Kalbe E Weisenbach S Hohmann C Vollmar S. Neurotransmitter changes in dementia with Lewy bodies and Parkinson disease dementia *in vivo*. Neurology. (2010) 74:885–92. 10.1212/WNL.0b013e3181d55f61 20181924

[39] Hu XS Okamura N Arai H Higuchi M Matsui T Tashiro M. 18F-fluorodopa PET study of striatal dopamine uptake in the diagnosis of dementia with Lewy bodies. Neurology. (2000) 55:1575–7. 10.1212/WNL.55.10.1575 11094120

[40] Lewis SJ Pavese N Rivero-Bosch M Eggert K Oertel W Mathias CJ. Brain monoamine systems in multiple system atrophy: a positron emission tomography study. Neurobiol Dis. (2012) 46:130–6. 10.1016/j.nbd.2011.12.053 22266105

[41] Tai YF Ahsan RL de Yébenes JG Pavese N Brooks DJ Piccini P. Characterization of dopaminergic dysfunction in familial progressive supranuclear palsy: an 18F-dopa PET study. J Neural Transm. (2007) 114:337–40. 10.1007/s00702-006-0536-0 16897607

[42] Kilbourn MR. *In vivo* radiotracers for vesicular neurotransmitter transporters. Nucl Med Biol. (1997) 24:615–9. 10.1016/S0969-8051(97)00101-7 9352531

[43] Martin WRW Wieler M Stoessl AJ Schulzer M. Dihydrotetrabenazine positron emission tomography imaging in early, untreated Parkinson's disease. Ann Neurol. (2008) 63:388–94. 10.1002/ana.21320 18240153

[44] Koeppe RA Gilman S Junck L Wernette K Frey KA. Differentiating Alzheimer's disease from dementia with Lewy bodies and Parkinson's disease with (+)-[11C]dihydrotetrabenazine positron emission tomography. Alzheimers Dement J Alzheimers Assoc. (2008) 4(1 Suppl. 1):S67–76. 10.1016/j.jalz.2007.11.016 18632004

[45] Gilman S Koeppe RA Little R An H Junck L Giordani B. Striatal monoamine terminals in Lewy body dementia and Alzheimer's disease. Ann Neurol. (2004) 55:774–80. 10.1002/ana.20088 15174011

[46] Gilman S Chervin RD Koeppe RA Consens FB Little R An H. Obstructive sleep apnea is related to a thalamic cholinergic deficit in MSA. Neurology. (2003) 61:35–9. 10.1212/01.WNL.0000073624.13436.32 12847153

[47] Gilman S Koeppe RA Chervin RD Consens FB Little R An H. REM sleep behavior disorder is related to striatal monoaminergic deficit in MSA. Neurology. (2003) 61:29–34. 10.1212/01.WNL.0000073745.68744.94 12847152

[48] Emond P Garreau L Chalon S Boazi M Caillet M Bricard J. Synthesis and ligand binding of nortropane derivatives: N-substituted 2beta-carbomethoxy-3beta-(4'-iodophenyl)nortropane and N-(3-iodoprop-(2E)-enyl)-2beta-carbomethoxy-3beta-(3',4'-disubstituted phenyl)nortropane. New high-affinity and selective compounds for the dopamine transporter. J Med Chem. (1997) 40:1366–72. 10.1021/jm960795d 9135033

[49] Jennings D Siderowf A Stern M Seibyl J Eberly S Oakes D. Imaging prodromal Parkinson disease: the Parkinson Associated Risk Syndrome Study. Neurology. (2014) 83:1739–46. 10.1212/WNL.0000000000000960 25298306 PMC4239830

[50] Olanow CW Jenner P Brooks D. Dopamine agonists and neuroprotection in Parkinson's disease. Ann Neurol. (1998) 44(3 Suppl. 1):S167–174. 10.1002/ana.410440725 9749590

[51] Parkinson Study Group Dopamine transporter brain imaging to assess the effects of pramipexole vs levodopa on Parkinson disease progression. JAMA. (2002) 287:1653–61. 10.1001/jama.287.13.1653 11926889

[52] Fahn S Oakes D Shoulson I Kieburtz K Rudolph A Lang A. Levodopa and the progression of Parkinson's disease. N Engl J Med. (2004) 351:2498–508. 10.1056/NEJMoa033447 15590952

[53] Lim SM Katsifis A Villemagne VL Best R Jones G Saling M. The 18F-FDG PET cingulate island sign and comparison to 123I-beta-CIT SPECT for diagnosis of dementia with Lewy bodies. J Nucl Med Off Publ Soc Nucl Med. (2009) 50:1638–45. 10.2967/jnumed.109.065870 19759102

[54] Tsao H-H Lin K-J Juang J-H Skovronsky DM Yen T-C Wey S-P. Binding characteristics of 9-fluoropropyl-(+)-dihydrotetrabenzazine (AV-133) to the vesicular monoamine transporter type 2 in rats. Nucl Med Biol. (2010) 37:413–9. 10.1016/j.nucmedbio.2010.01.002 20447551

[55] Hsiao I-T Weng Y-H Hsieh C-J Lin W-Y Wey S-P Kung M-P. Correlation of Parkinson disease severity and 18F-DTBZ positron emission tomography. JAMA Neurol. (2014) 71:758–66. 10.1001/jamaneurol.2014.290 24756323

[56] Villemagne VL Okamura N Pejoska S Drago J Mulligan RS Chételat G. *In vivo* assessment of vesicular monoamine transporter type 2 in dementia with lewy bodies and Alzheimer disease. Arch Neurol. (2011) 68:905–12. 10.1001/archneurol.2011.142 21747030

[57] Moccia M Erro R Picillo M Santangelo G Spina E Allocca R. A four-year longitudinal study on restless legs syndrome in Parkinson Disease. Sleep. (2016) 39:405–12. 10.5665/sleep.5452 26564123 PMC4712388

[58] Filippi L Manni C Pierantozzi M Brusa L Danieli R Stanzione P. 123I-FP-CIT in progressive supranuclear palsy and in Parkinson's disease: a SPECT semiquantitative study. Nucl Med Commun. (2006) 27:381–6. 10.1097/01.mnm.0000202858.45522.df 16531926

[59] Booij J Tissingh G Boer GJ Speelman JD Stoof JC Janssen AG. [123I]FP-CIT SPECT shows a pronounced decline of striatal dopamine transporter labelling in early and advanced Parkinson's disease. J Neurol Neurosurg Psychiatry. (1997) 62:133–40. 10.1136/jnnp.62.2.133 9048712 PMC486723

[60] Walker Z Moreno E Thomas A Inglis F Tabet N Rainer M. Clinical usefulness of dopamine transporter SPECT imaging with 123I-FP-CIT in patients with possible dementia with Lewy bodies: randomised study. Br J Psychiatry J Ment Sci. (2015) 206:145–52. 10.1192/bjp.bp.114.148643 25431431

[61] El Fakhri G Habert M-O Maksud P Kas A Malek Z Kijewski MF. Quantitative simultaneous (99m)Tc-ECD/123I-FP-CIT SPECT in Parkinson's disease and multiple system atrophy. Eur J Nucl Med Mol Imaging. (2006) 33:87–92. 10.1007/s00259-005-1920-y 16180033

[62] Brücke T Yuan Feen Tsai McLellan C Singhanyom W Kung HF Cohen RM. *In vitro* binding properties and autoradiographic imaging of 3-iodobenzamide ([125I]-IBZM): A potential imaging ligand for D-2 dopamine receptors in spect. Life Sci. (1988) 42:2097–104. 10.1016/0024-3205(88)90123-3 3260318

[63] van Royen E Verhoeff NF Speelman JD Wolters EC Kuiper MA Janssen AG. Multiple system atrophy and progressive supranuclear palsy. Diminished striatal D2 dopamine receptor activity demonstrated by 123I-IBZM single photon emission computed tomography. Arch Neurol. (1993) 50:513–6. 10.1001/archneur.1993.00540050063017 8489409

[64] Seppi K Schocke MFH Donnemiller E Esterhammer R Kremser C Scherfler C. Comparison of diffusion-weighted imaging and [123I]IBZM-SPECT for the differentiation of patients with the Parkinson variant of multiple system atrophy from those with Parkinson's disease. Mov Disord Off J Mov Disord Soc. (2004) 19:1438–45. 10.1002/mds.20229 15390073

[65] Arnold G Tatsch K Oertel WH Vogl T Schwarz J Kraft E. Clinical progressive supranuclear palsy: differential diagnosis by IBZM-SPECT and MRI. J Neural Transm Suppl. (1994) 42:111–8. 10.1007/978-3-7091-6641-3_9 7964681

[66] Van Laere K De Ceuninck L Dom R Van den Eynden J Vanbilloen H Cleynhens J. Dopamine transporter SPECT using fast kinetic ligands: 123I-FP-beta-CIT versus 99mTc-TRODAT-1. Eur J Nucl Med Mol Imaging. (2004) 31:1119–27. 10.1007/s00259-004-1480-6 15064872

[67] Kung MP Kung HF Billings J Yang Y Murphy RA Alavi A. The characterization of IBF as a new selective dopamine D-2 receptor imaging agent. J Nucl Med Off Publ Soc Nucl Med. (1990) 31:648–54. 2140409

[68] Kim YJ Ichise M Ballinger JR Vines D Erami SS Tatschida T. Combination of dopamine transporter and D2 receptor SPECT in the diagnostic evaluation of PD, MSA, and PSP. Mov Disord Off J Mov Disord Soc. (2002) 17:303–12. 10.1002/mds.10042 11921116

[69] Oyanagi C Katsumi Y Hanakawa T Hayashi T Thuy D ha D Hashikawa K Comparison of striatal dopamine D2 receptors in Parkinson's disease and progressive supranuclear palsy patients using [123I] iodobenzofuran single-photon emission computed tomography. J Neuroimaging Off J Am Soc Neuroimaging. (2002) 12:316–24. 10.1111/j.1552-6569.2002.tb00139.x 12380478

[70] Sonni I Fazio P Schain M Halldin C Svenningsson P Farde L. Optimal acquisition time window and simplified quantification of dopamine transporter availability using 18F-FE-PE2I in healthy controls and Parkinson disease patients. J Nucl Med Off Publ Soc Nucl Med. (2016) 57:1529–34. 10.2967/jnumed.115.171231 27230923

[71] Prunier C Payoux P Guilloteau D Chalon S Giraudeau B Majorel C. Quantification of dopamine transporter by 123I-PE2I SPECT and the noninvasive Logan graphical method in Parkinson's disease. J Nucl Med Off Publ Soc Nucl Med. (2003) 44:663–70. 12732666

[72] Ziebell M Andersen BB Pinborg LH Knudsen GM Stokholm J Thomsen G Striatal dopamine transporter binding does not correlate with clinical severity in dementia with Lewy bodies. J Nucl Med Off Publ Soc Nucl Med. (2013) 54:1072–6. 10.2967/jnumed.112.114025 23637201

[73] Warren NM Piggott MA Greally E Lake M Lees AJ Burn DJ. Basal ganglia cholinergic and dopaminergic function in progressive supranuclear palsy. Mov Disord Off J Mov Disord Soc. (2007) 22:1594–600. 10.1002/mds.21573 17534953

[74] Hall H Ogren SO Köhler C Magnusson O. Animal pharmacology of raclopride, a selective dopamine D2 antagonist. Psychopharmacol Ser. (1989) 7:123–30. 10.1007/978-3-642-74430-3_13 2687851

[75] Antonini A Leenders KL Vontobel P Maguire RP Missimer J Psylla M. Complementary PET studies of striatal neuronal function in the differential diagnosis between multiple system atrophy and Parkinson's disease. Brain J Neurol. (1997) 120 (Pt 12):2187–95. 10.1093/brain/120.12.2187 9448574

[76] Van Laere K Clerinx K D'Hondt E de Groot T Vandenberghe W. Combined striatal binding and cerebral influx analysis of dynamic 11C-raclopride PET improves early differentiation between multiple-system atrophy and Parkinson disease. J Nucl Med Off Publ Soc Nucl Med. (2010) 51:588–95. 10.2967/jnumed.109.070144 20237023

[77] Sérrière S Tauber C Vercouillie J Guilloteau D Deloye J-B Garreau L. *In vivo* PET quantification of the dopamine transporter in rat brain with [^18^F]LBT-999. Nucl Med Biol. (2014) 41:106–13. 10.1016/j.nucmedbio.2013.09.007 24210285

[78] Mukherjee J Yang Z-Y Brown T Roemer J Cooper M. 18F-desmethoxyfallypride: A fluorine-18 labeled radiotracer with properties similar to carbon-11 raclopride for pet imaging studies of dopamine D2 receptors. Life Sci. (1996) 59:669–78. 10.1016/0024-3205(96)00348-7 8761017

[79] Werhahn KJ Landvogt C Klimpe S Buchholz H-G Yakushev I Siessmeier T. Decreased dopamine D2/D3-receptor binding in temporal lobe epilepsy: an [18F]fallypride PET study. Epilepsia. (2006) 47:1392–6. 10.1111/j.1528-1167.2006.00561.x 16922886

[80] Mathis CA Mason NS Lopresti BJ Klunk WE. Development of positron emission tomography β-amyloid plaque imaging agents. Semin Nucl Med. (2012) 42:423–32. 10.1053/j.semnuclmed.2012.07.001 23026364 PMC3520098

[81] Jack CR Knopman DS Jagust WJ Shaw LM Aisen PS Weiner MW. Hypothetical model of dynamic biomarkers of the Alzheimer's pathological cascade. Lancet Neurol. (2010) 9:119–28. 10.1016/S1474-4422(09)70299-6 20083042 PMC2819840

[82] Klunk WE Engler H Nordberg A Wang Y Blomqvist G Holt DP. Imaging brain amyloid in Alzheimer's disease with Pittsburgh Compound-B. Ann Neurol. (2004) 55:306–19. 10.1002/ana.20009 14991808

[83] Bacskai BJ Frosch MP Freeman SH Raymond SB Augustinack JC Johnson KA. Molecular imaging with Pittsburgh Compound B confirmed at autopsy: a case report. Arch Neurol. (2007) 64:431–4. 10.1001/archneur.64.3.431 17353389

[84] Hatashita S Yamasaki H Clinically different stages of Alzheimer's disease associated by amyloid deposition with [11C]-PIB PET imaging. J Alzheimers Dis JAD. (2010) 21:995–1003. 10.3233/JAD-2010-100222 20693641

[85] Fagan AM Mintun MA Mach RH Lee S-Y Dence CS Shah AR. Inverse relation between *in vivo* amyloid imaging load and cerebrospinal fluid Abeta42 in humans. Ann Neurol. (2006) 59:512–9. 10.1002/ana.20730 16372280

[86] Chételat G Villemagne VL Bourgeat P Pike KE Jones G Ames D. Relationship between atrophy and beta-amyloid deposition in Alzheimer disease. Ann Neurol. (2010) 67:317–24. 10.1002/ana.21955 20373343

[87] Pike KE Savage G Villemagne VL Ng S Moss SA Maruff P. Beta-amyloid imaging and memory in non-demented individuals: evidence for preclinical Alzheimer's disease. Brain J Neurol. (2007) 130(Pt 11):2837–44. 10.1093/brain/awm238 17928318

[88] Payoux P Salabert AS. New PET markers for the diagnosis of dementia. Curr Opin Neurol. (2017) 30:608–16. 10.1097/WCO.0000000000000489 28906268

[89] Newberg AB Arnold SE Wintering N Rovner BW Alavi A. Initial clinical comparison of 18F-florbetapir and 18F-FDG PET in patients with Alzheimer disease and controls. J Nucl Med Off Publ Soc Nucl Med. (2012) 53:902–7. 10.2967/jnumed.111.099606 22577238

[90] Del Campo N Payoux P Djilali A Delrieu J Hoogendijk EO Rolland Y. Relationship of regional brain β-amyloid to gait speed. Neurology. (2016) 86:36–43. 10.1212/WNL.0000000000002235 26643548 PMC4731288

[91] Bailly M Ribeiro MJS Vercouillie J Hommet C Gissot V Camus V. 18F-FDG and 18F-florbetapir PET in clinical practice: regional analysis in mild cognitive impairment and Alzheimer disease. Clin Nucl Med. (2015) 40:e111–6. 10.1097/RLU.0000000000000666 25549345

[92] Kobylecki C Langheinrich T Hinz R Vardy ERLC Brown G Martino M-E. 18F-florbetapir PET in patients with frontotemporal dementia and Alzheimer disease. J Nucl Med Off Publ Soc Nucl Med. (2015) 56:386–91. 10.2967/jnumed.114.147454 25655625

[93] de Lartigue J. Flutemetamol (18F): a β-amyloid positron emission tomography tracer for Alzheimer's and dementia diagnosis. Drugs Today. (2014) 50:219–29. 10.1358/dot.2014.050.03.2116672 24696867

[94] Duara R Loewenstein DA Shen Q Barker W Potter E Varon D. Amyloid positron emission tomography with (18)F-flutemetamol and structural magnetic resonance imaging in the classification of mild cognitive impairment and Alzheimer's disease. Alzheimers Dement J Alzheimers Assoc. (2013) 9:295–301. 10.1016/j.jalz.2012.01.006 23178035 PMC5610964

[95] Morris E Chalkidou A Hammers A Peacock J Summers J Keevil S. Diagnostic accuracy of (18)F amyloid PET tracers for the diagnosis of Alzheimer's disease: a systematic review and meta-analysis. Eur J Nucl Med Mol Imaging. (2016) 43:374–85. 10.1007/s00259-015-3228-x 26613792 PMC4700091

[96] Barthel H Luthardt J Becker G Patt M Hammerstein E Hartwig K. Individualized quantification of brain β-amyloid burden: results of a proof of mechanism phase 0 florbetaben PET trial in patients with Alzheimer's disease and healthy controls. Eur J Nucl Med Mol Imaging. (2011) 38:1702–14. 10.1007/s00259-011-1821-1 21547601

[97] Chiaravalloti A Danieli R Lacanfora A Palumbo B Caltagirone C Schillaci O. Usefulness of 18F florbetaben in diagnosis of Alzheimer's disease and other types of dementia. Curr Alzheimer Res. (2017) 14:154–60. 10.2174/1567205013666160620114309 27334940

[98] Sabri O Sabbagh MN Seibyl J Barthel H Akatsu H Ouchi Y. Florbetaben PET imaging to detect amyloid beta plaques in Alzheimer's disease: phase 3 study. Alzheimers Dement J Alzheimers Assoc. (2015) 11:964–74. 10.1016/j.jalz.2015.02.004 25824567

[99] Ong KT Villemagne VL Bahar-Fuchs A Lamb F Langdon N Catafau AM. Aβ imaging with 18F-florbetaben in prodromal Alzheimer's disease: a prospective outcome study. J Neurol Neurosurg Psychiatry. (2015) 86:431–6. 10.1136/jnnp-2014-308094 24970906

[100] Knopman DS DeKosky ST Cummings JL Chui H Corey-Bloom J Relkin N. Practice parameter: diagnosis of dementia (an evidence-based review). Report of the Quality Standards Subcommittee of the American Academy of Neurology. Neurology. (2001) 56:1143–53. 10.1212/WNL.56.9.1143 11342678

[101] Yeo JM Waddell B Khan Z Pal S. A systematic review and meta-analysis of (18)F-labeled amyloid imaging in Alzheimer's disease. Alzheimers Dement Amst Neth. (2015) 1:5–13. 10.1016/j.dadm.2014.11.004 27239488 PMC4876886

[102] Rabinovici GD Furst AJ Alkalay A Racine CA O'Neil JP Janabi M Increased metabolic vulnerability in early-onset Alzheimer's disease is not related to amyloid burden. Brain J Neurol. (2010) 133(Pt 2):512–28. 10.1093/brain/awp326 PMC2858015 20080878

[103] Lehmann M Ghosh PM Madison C Laforce R Corbetta-Rastelli C Weiner MW. Diverging patterns of amyloid deposition and hypometabolism in clinical variants of probable Alzheimer's disease. Brain J Neurol. (2013) 136(Pt 3):844–58. 10.1093/brain/aws327 23358601 PMC3580269

[104] Jung Y Whitwell JL Duffy JR Strand EA Machulda MM Senjem ML. Regional β-amyloid burden does not correlate with cognitive or language deficits in Alzheimer's disease presenting as aphasia. Eur J Neurol. (2016) 23:313–9. 10.1111/ene.12761 26101072 PMC4689664

[105] Laforce R Soucy J-P Sellami L Dallaire-Théroux C Brunet F Bergeron D. Molecular imaging in dementia: Past, present, and future. Alzheimers Dement. (2018) 14:1522–52. 10.1016/j.jalz.2018.06.2855 30028955

[106] Johnson KA Minoshima S Bohnen NI Donohoe KJ Foster NL Herscovitch P. Appropriate use criteria for amyloid PET: a report of the Amyloid Imaging Task Force, the Society of Nuclear Medicine and Molecular Imaging, and the Alzheimer's Association. Alzheimers Dement. (2013) 9:e-1-16. 10.1016/j.jalz.2013.01.002 23360977 PMC3733252

[107] Weller J Budson A. Current understanding of Alzheimer's disease diagnosis and treatment. F1000Res. (2018) 7:F1000 Faculty Rev-1161. 10.12688/f1000research.14506.1 30135715 PMC6073093

[108] Delrieu J Ousset PJ Voisin T Vellas B. Amyloid beta peptide immunotherapy in Alzheimer disease. Rev Neurol. (2014) 170:739–48. 10.1016/j.neurol.2014.10.003 25459121

[109] Honig LS Vellas B Woodward M Boada M Bullock R Borrie M. Trial of solanezumab for mild dementia due to Alzheimer's disease. N Engl J Med. (2018) 378:321–30. 10.1056/NEJMoa1705971 29365294

[110] Hall B Mak E Cervenka S Aigbirhio FI Rowe JB O'Brien JT. *In vivo* tau PET imaging in dementia: pathophysiology, radiotracer quantification, and a systematic review of clinical findings. Ageing Res Rev. (2017) 36:50–63. 10.1016/j.arr.2017.03.002 28315409

[111] Gómez-Isla T Hollister R West H Mui S Growdon JH Petersen RC. Neuronal loss correlates with but exceeds neurofibrillary tangles in Alzheimer's disease. Ann Neurol. (1997) 41:17–24. 10.1002/ana.410410106 9005861

[112] van Rossum IA Visser PJ Knol DL van der Flier WM Teunissen CE Barkhof F Injury markers but not amyloid markers are associated with rapid progression from mild cognitive impairment to dementia in Alzheimer's disease. J Alzheimers Dis JAD. (2012) 29:319–27. 10.3233/JAD-2011-111694 22233766

[113] Agdeppa ED Kepe V Liu J Flores-Torres S Satyamurthy N Petric A. Binding characteristics of radiofluorinated 6-dialkylamino-2-naphthylethylidene derivatives as positron emission tomography imaging probes for beta-amyloid plaques in Alzheimer's disease. J Neurosci Off J Soc Neurosci. (2001) 21:RC189. 10.1523/JNEUROSCI.21-24-j0004.2001 11734604 PMC6763047

[114] Luurtsema G Schuit RC Takkenkamp K Lubberink M Hendrikse NH Windhorst AD. Peripheral metabolism of [(18)F]FDDNP and cerebral uptake of its labelled metabolites. Nucl Med Biol. (2008) 35:869–74. 10.1016/j.nucmedbio.2008.09.002 19026948

[115] Okamura N Suemoto T Furumoto S Suzuki M Shimadzu H Akatsu H. Quinoline and benzimidazole derivatives: candidate probes for *in vivo* imaging of tau pathology in Alzheimer's disease. J Neurosci Off J Soc Neurosci. (2005) 25:10857–62. 10.1523/JNEUROSCI.1738-05.2005 16306398 PMC6725872

[116] Fodero-Tavoletti MT Okamura N Furumoto S Mulligan RS Connor AR McLean CA. 18F-THK523: a novel *in vivo* tau imaging ligand for Alzheimer's disease. Brain J Neurol. (2011) 134(Pt 4):1089–100. 10.1093/brain/awr038 21436112

[117] Fodero-Tavoletti MT Furumoto S Taylor L McLean CA Mulligan RS Birchall I. Assessing THK523 selectivity for tau deposits in Alzheimer's disease and non-Alzheimer's disease tauopathies. Alzheimers Res Ther. (2014) 6:11. 10.1186/alzrt240 24572336 PMC3979096

[118] Villemagne VL Furumoto S Fodero-Tavoletti MT Mulligan RS Hodges J Harada R. *In vivo* evaluation of a novel tau imaging tracer for Alzheimer's disease. Eur J Nucl Med Mol Imaging. (2014) 41:816–26. 10.1007/s00259-013-2681-7 24514874

[119] Harada R Okamura N Furumoto S Furukawa K Ishiki A Tomita N. 18F-THK5351: a novel PET radiotracer for imaging neurofibrillary pathology in Alzheimer disease. J Nucl Med Off Publ Soc Nucl Med. (2016) 57:208–14. 10.2967/jnumed.115.164848 26541774

[120] Chien DT Bahri S Szardenings AK Walsh JC Mu F Su M-Y. Early clinical PET imaging results with the novel PHF-tau radioligand [F-18]-T807. J Alzheimers Dis JAD. (2013) 34:457–68. 10.3233/JAD-122059 23234879

[121] Chien DT Szardenings AK Bahri S Walsh JC Mu F Xia C. Early clinical PET imaging results with the novel PHF-tau radioligand [F18]-T808. J Alzheimers Dis JAD. (2014) 38:171–84. 10.3233/JAD-130098 23948934

[122] Johnson KA Schultz A Betensky RA Becker JA Sepulcre J Rentz D. Tau positron emission tomographic imaging in aging and early Alzheimer disease. Ann Neurol. (2016) 79:110–9. 10.1002/ana.24546 26505746 PMC4738026

[123] Brier MR Gordon B Friedrichsen K McCarthy J Stern A Christensen J. Tau and Aβ imaging, CSF measures, and cognition in Alzheimer's disease. Sci Transl Med. (2016) 8:338ra66. 10.1126/scitranslmed.aaf2362 27169802 PMC5267531

[124] Chhatwal JP Schultz AP Marshall GA Boot B Gomez-Isla T Dumurgier J. Temporal T807 binding correlates with CSF tau and phospho-tau in normal elderly. Neurology. (2016) 87:920–6. 10.1212/WNL.0000000000003050 27473132 PMC5035159

[125] Lowe VJ Curran G Fang P Liesinger AM Josephs KA Parisi JE. An autoradiographic evaluation of AV-1451 Tau PET in dementia. Acta Neuropathol Commun. (2016) 4:58. 10.1186/s40478-016-0315-6 27296779 PMC4906968

[126] Walji AM Hostetler ED Selnick H Zeng Z Miller P Bennacef I. Discovery of 6-(Fluoro-(18)F)-3-(1H-pyrrolo[2,3-c]pyridin-1-yl)isoquinolin-5-amine ([(18)F]-MK-6240): a Positron Emission Tomography (PET) Imaging Agent for Quantification of Neurofibrillary Tangles (NFTs). J Med Chem. (2016) 59:4778–89. 10.1021/acs.jmedchem.6b00166 27088900

[127] Hostetler ED Walji AM Zeng Z Miller P Bennacef I Salinas C. Preclinical characterization of 18F-MK-6240, a promising PET tracer for *in vivo* quantification of human neurofibrillary tangles. J Nucl Med Off Publ Soc Nucl Med. (2016) 57:1599–606. 10.2967/jnumed.115.171678 27230925

[128] Lohith TG Bennacef I Vandenberghe R Vandenbulcke M Salinas-Valenzuela C Declercq R First-in-human brain imaging of Alzheimer dementia patients and elderly controls with 18F-MK-6240, a PET tracer targeting neurofibrillary tangle pathology. J Nucl Med Off Publ Soc Nucl Med. (2019) 60:107–14. 10.2967/jnumed.118.208215 29880509

[129] Pedersen SF Sandholt BV Keller SH Hansen AE Clemmensen AE Sillesen H. 64Cu-DOTATATE PET/MRI for detection of activated macrophages in carotid atherosclerotic plaques. Arterioscler Thromb Vasc Biol. (2015) 35:1696–703. 10.1161/ATVBAHA.114.305067 25977567 PMC4479665

[130] Brosch JR Farlow MR Risacher SL Apostolova LG. Tau imaging in alzheimer's disease diagnosis and clinical trials. Neurotherapeutics. (2017) 14:62–8. 10.1007/s13311-016-0490-y 27873182 PMC5233632

[131] Rosenmann H. Immunotherapy for targeting tau pathology in Alzheimer's disease and tauopathies. Curr Alzheimer Res. (2013) 10:217–28. 10.2174/1567205011310030001 23534533

[132] Novak P Schmidt R Kontsekova E Zilka N Kovacech B Skrabana R. Safety and immunogenicity of the tau vaccine AADvac1 in patients with Alzheimer's disease: a randomised, double-blind, placebo-controlled, phase 1 trial. Lancet Neurol. (2017) 16:123–34. 10.1016/S1474-4422(16)30331-3 27955995

[133] Panza F Solfrizzi V Seripa D Imbimbo BP Lozupone M Santamato A. Tau-based therapeutics for Alzheimer's disease: active and passive immunotherapy. Immunotherapy. (2016) 8:1119–34. 10.2217/imt-2016-0019 27485083

[134] Barret O Alagille D Sanabria S Comley RA Weimer RM Borroni E. Kinetic modeling of the Tau PET tracer 18F-AV-1451 in human healthy volunteers and Alzheimer disease subjects. J Nucl Med Off Publ Soc Nucl Med. (2017) 58:1124–31. 10.2967/jnumed.116.182881 27908967

[135] Maass A Landau S Baker SL Horng A Lockhart SN La Joie R. Comparison of multiple tau-PET measures as biomarkers in aging and Alzheimer's disease. NeuroImage. (2017) 157:448–63. 10.1016/j.neuroimage.2017.05.058 28587897 PMC5814575

[136] Bejanin A Schonhaut DR La Joie R Kramer JH Baker SL Sosa N. Tau pathology and neurodegeneration contribute to cognitive impairment in Alzheimer's disease. Brain J Neurol. (2017) 140:3286–300. 10.1093/brain/awx243 29053874 PMC5841139

[137] Ossenkoppele R Rabinovici GD Smith R Cho H Schöll M Strandberg O. Discriminative accuracy of [18F]flortaucipir positron emission tomography for alzheimer disease vs other neurodegenerative disorders. JAMA. (2018) 320:1151–62. 10.1001/jama.2018.12917 30326496 PMC6233630

[138] Zhang W Arteaga J Cashion DK Chen G Gangadharmath U Gomez LF. A highly selective and specific PET tracer for imaging of tau pathologies. J Alzheimers Dis JAD. (2012) 31:601–12. 10.3233/JAD-2012-120712 22683529

[139] Choe YS Lee K-H. PET radioligands for imaging of tau pathology: current status. Nucl Med Mol Imaging. (2015) 49:251–7. 10.1007/s13139-015-0374-9 26550043 PMC4630339

[140] Harada R Okamura N Furumoto S Furukawa K Ishiki A Tomita N. [(18)F]THK-5117 PET for assessing neurofibrillary pathology in Alzheimer's disease. Eur J Nucl Med Mol Imaging. (2015) 42:1052–61. 10.1007/s00259-015-3035-4 25792456

[141] Okamura N Furumoto S Harada R Tago T Yoshikawa T Fodero-Tavoletti M. Novel 18F-labeled arylquinoline derivatives for noninvasive imaging of tau pathology in Alzheimer disease. J Nucl Med Off Publ Soc Nucl Med. (2013) 54:1420–7. 10.2967/jnumed.112.117341 23857514

[142] Okamura N Furumoto S Fodero-Tavoletti MT Mulligan RS Harada R Yates P. Non-invasive assessment of Alzheimer's disease neurofibrillary pathology using 18F-THK5105 PET. Brain J Neurol. (2014) 137(Pt 6):1762–71. 10.1093/brain/awu064 24681664

[143] Shimada H Kitamura S Shinotoh H Endo H Niwa F Hirano S Association between Aβ and tau accumulations and their influence on clinical features in aging and Alzheimer's disease spectrum brains: A [11C]PBB3-PET study. Alzheimers Dement Amst Neth. (2017) 6:11–20. 10.1016/j.jalz.2016.06.998 PMC5257028 28138509

[144] Perez-Soriano A Arena JE Dinelle K Miao Q McKenzie J Neilson N. PBB3 imaging in Parkinsonian disorders: evidence for binding to tau and other proteins. Mov Disord Off J Mov Disord Soc. (2017) 32:1016–24. 10.1002/mds.27029 28568506

[145] Shinotoh H Shimada H Kokubo Y Tagai K Niwa F Kitamura S. Tau imaging detects distinctive distribution of tau pathology in ALS/PDC on the Kii Peninsula. Neurology. (2019) 92:e136–47. 10.1212/WNL.0000000000006736 30530797 PMC6340344

[146] Narayanaswami V Dahl K Bernard-Gauthier V Josephson L Cumming P Vasdev N. Emerging PET radiotracers and targets for imaging of neuroinflammation in neurodegenerative diseases: outlook beyond TSPO. Mol Imaging. (2018) 17:1536012118792317. 10.1177/1536012118792317 30203712 PMC6134492

[147] Vivash L O'Brien TJ. Imaging microglial activation with TSPO PET: lighting up neurologic diseases? J Nucl Med Off Publ Soc Nucl Med. (2016) 57:165–8. 10.2967/jnumed.114.141713 26697963

[148] Dupont A-C Largeau B Santiago Ribeiro MJ Guilloteau D Tronel C Arlicot N. Translocator Protein-18 kDa (TSPO) Positron Emission Tomography (PET) imaging and its clinical impact in neurodegenerative diseases. Int J Mol Sci. (2017) 18:E785. 10.3390/ijms18040785 28387722 PMC5412369

[149] Fan Z Calsolaro V Atkinson RA Femminella GD Waldman A Buckley C. Flutriciclamide (18F-GE180) PET: first-in-human PET study of novel third-generation *in vivo* marker of human translocator protein. J Nucl Med Off Publ Soc Nucl Med. (2016) 57:1753–9. 10.2967/jnumed.115.169078 27261523

[150] Ikawa M Lohith TG Shrestha S Telu S Zoghbi SS Castellano S. 11C-ER176, a radioligand for 18-kDa translocator protein, has adequate sensitivity to robustly image all three affinity genotypes in human brain. J Nucl Med Off Publ Soc Nucl Med. (2017) 58:320–5. 10.2967/jnumed.116.178996 27856631 PMC5288742

[151] Rodriguez-Vieitez E Ni R Gulyás B Tóth M Häggkvist J Halldin C. Astrocytosis precedes amyloid plaque deposition in Alzheimer APPswe transgenic mouse brain: a correlative positron emission tomography and *in vitro* imaging study. Eur J Nucl Med Mol Imaging. (2015) 42:1119–32. 10.1007/s00259-015-3047-0 25893384 PMC4424277

[152] Shukuri M Takashima-Hirano M Tokuda K Takashima T Matsumura K Inoue O. *In vivo* expression of cyclooxygenase-1 in activated microglia and macrophages during neuroinflammation visualized by PET with 11C-ketoprofen methyl ester. J Nucl Med Off Publ Soc Nucl Med. (2011) 52:1094–101. 10.2967/jnumed.110.084046 21680698

[153] Ohnishi A Senda M Yamane T Mikami T Nishida H Nishio T. Exploratory human PET study of the effectiveness of (11)C-ketoprofen methyl ester, a potential biomarker of neuroinflammatory processes in Alzheimer's disease. Nucl Med Biol. (2016) 43:438–44. 10.1016/j.nucmedbio.2016.04.005 27183464

[154] Boutagy NE Wu J Cai Z Zhang W Booth CJ Kyriakides TC. *In vivo* reactive oxygen species detection with a novel positron emission tomography tracer, 18F-DHMT, allows for early detection of anthracycline-induced cardiotoxicity in rodents. JACC Basic Transl Sci. (2018) 3:378–90. 10.1016/j.jacbts.2018.02.003 30062224 PMC6058999

[155] Hou C Hsieh C-J Li S Lee H Graham TJ Xu K. Development of a positron emission tomography radiotracer for imaging elevated levels of superoxide in neuroinflammation. ACS Chem Neurosci. (2018) 9:578–86. 10.1021/acschemneuro.7b00385 29099578 PMC5865080

[156] Alam MM Lee J Lee S-Y. Recent progress in the development of TSPO PET ligands for neuroinflammation imaging in neurological diseases. Nucl Med Mol Imaging. (2017) 51:283–96. 10.1007/s13139-017-0475-8 29242722 PMC5721086

[157] Passamonti L Rodríguez PV Hong YT Allinson KSJ Bevan-Jones WR Williamson D. [11C]PK11195 binding in Alzheimer disease and progressive supranuclear palsy. Neurology. (2018) 90:e1989–96. 10.1212/WNL.0000000000005610 29703774 PMC5980519

[158] Edison P Archer HA Gerhard A Hinz R Pavese N Turkheimer FE Microglia, amyloid, and cognition in Alzheimer's disease: An [11C](R)PK11195-PET and [11C]PIB-PET study. Neurobiol Dis. (2008) 32:412–9. 10.1016/j.nbd.2008.08.001 18786637

[159] Hamelin L Lagarde J Dorothée G Leroy C Labit M Comley RA. Early and protective microglial activation in Alzheimer's disease: a prospective study using 18F-DPA-714 PET imaging. Brain J Neurol. (2016) 139(Pt 4):1252–64. 10.1093/brain/aww017 26984188

[160] Golla SSV Boellaard R Oikonen V Hoffmann A van Berckel BNM Windhorst AD Quantification of [18F]DPA-714 binding in the human brain: initial studies in healthy controls and Alzheimer's disease patients. J Cereb Blood Flow Metab Off J Int Soc Cereb Blood Flow Metab. (2015) 35:766–72. 10.1038/jcbfm.2014.261 PMC4420859 25649991

[161] Varrone A Mattsson P Forsberg A Takano A Nag S Gulyás B *In vivo* imaging of the 18-kDa translocator protein (TSPO) with [18F]FEDAA1106 and PET does not show increased binding in Alzheimer's disease patients. Eur J Nucl Med Mol Imaging. (2013) 40:921–31. 10.1007/s00259-013-2359-1 23436070

[162] Takano A Piehl F Hillert J Varrone A Nag S Gulyás B *In vivo* TSPO imaging in patients with multiple sclerosis: a brain PET study with [18F]FEDAA1106. EJNMMI Res. (2013) 3:30 10.1186/2191-219X-3-30 23618062 PMC3640965

[163] Wadsworth H Jones PA Chau W-F Durrant C Fouladi N Passmore J [^18^F]GE-180: a novel fluorine-18 labelled PET tracer for imaging Translocator protein 18 kDa (TSPO). Bioorg Med Chem Lett. (2012) 22:1308–13. 10.1016/j.bmcl.2011.12.084 22244939

[164] Vomacka L Albert NL Lindner S Unterrainer M Mahler C Brendel M. TSPO imaging using the novel PET ligand [18F]GE-180: quantification approaches in patients with multiple sclerosis. EJNMMI Res. (2017) 7:89. 10.1186/s13550-017-0340-x 29150726 PMC5693838

[165] Suridjan I Pollock BG Verhoeff NPLG Voineskos AN Chow T Rusjan PM *In-vivo* imaging of grey and white matter neuroinflammation in Alzheimer's disease: a positron emission tomography study with a novel radioligand, [18F]-FEPPA. Mol Psychiatry. (2015) 20:1579–87. 10.1038/mp.2015.1 25707397 PMC8026116

[166] Rodriguez-Vieitez E Saint-Aubert L Carter SF Almkvist O Farid K Schöll M. Diverging longitudinal changes in astrocytosis and amyloid PET in autosomal dominant Alzheimer's disease. Brain J Neurol. (2016) 139(Pt 3):922–36. 10.1093/brain/awv404 26813969 PMC4766380

[167] Singhal T O'Connor K Dubey S Belanger AP Hurwitz S Chu R. 18F-PBR06 versus 11C-PBR28 PET for assessing white matter translocator protein binding in multiple sclerosis. Clin Nucl Med. (2018) 43:e289–95. 10.1097/RLU.0000000000002179 30004939

[168] Colasanti A Guo Q Muhlert N Giannetti P Onega M Newbould RD. *In vivo* assessment of brain white matter inflammation in multiple sclerosis with (18)F-PBR111 PET. J Nucl Med Off Publ Soc Nucl Med. (2014) 55:1112–8. 10.2967/jnumed.113.135129 24904112

[169] Ottoy J De Picker L Verhaeghe J Deleye S Wyffels L Kosten L. 18F-PBR111 PET imaging in healthy controls and schizophrenia: test-retest reproducibility and quantification of neuroinflammation. J Nucl Med Off Publ Soc Nucl Med. (2018) 59:1267–74. 10.2967/jnumed.117.203315 29326362

[170] Amhaoul H Hamaide J Bertoglio D Reichel SN Verhaeghe J Geerts E. Brain inflammation in a chronic epilepsy model: Evolving pattern of the translocator protein during epileptogenesis. Neurobiol Dis. (2015) 82:526–39. 10.1016/j.nbd.2015.09.004 26388398

[171] Fuchigami T Nakayama M Yoshida S. Development of PET and SPECT probes for glutamate receptors. ScientificWorldJournal. (2015) 2015:716514. 10.1155/2015/716514 25874256 PMC4385697

[172] Bressan RA Erlandsson K Mulligan RS Gunn RN Cunningham VJ Owens J. Evaluation of NMDA receptors *in vivo* in schizophrenic patients with [123I]CNS 1261 and SPET: preliminary findings. Ann N Y Acad Sci. (2003) 1003:364–7. 10.1196/annals.1300.027 14684462

[173] Bressan RA Erlandsson K Stone JM Mulligan RS Krystal JH Ell PJ. Impact of schizophrenia and chronic antipsychotic treatment on [123I]CNS-1261 binding to N-methyl-D-aspartate receptors *in vivo*. Biol Psychiatry. (2005) 58:41–6. 10.1016/j.biopsych.2005.03.016 15992521

[174] Zanotti-Fregonara P Xu R Zoghbi SS Liow J-S Fujita M Veronese M. The PET radioligand 18F-FIMX images and quantifies metabotropic glutamate receptor 1 in proportion to the regional density of its gene transcript in human brain. J Nucl Med Off Publ Soc Nucl Med. (2016) 57:242–7. 10.2967/jnumed.115.162461 26514176 PMC4983720

[175] Barret O Tamagnan G Batis J Jennings D Zubal G Russell D Quantitation of glutamate mGluR5 receptor with 18F-FPEB PET in humans. J Nucl Med. (2010) 51(Suppl. 2):215 10.1016/j.neuroimage.2010.04.164

[176] Leurquin-Sterk G Ceccarini J Crunelle CL de Laat B Verbeek J Deman S. Lower limbic metabotropic glutamate receptor 5 availability in alcohol dependence. J Nucl Med Off Publ Soc Nucl Med. (2018) 59:682–90. 10.2967/jnumed.117.199422 29348321

[177] Abdallah CG Hannestad J Mason GF Holmes SE DellaGioia N Sanacora G. Metabotropic glutamate receptor 5 and glutamate involvement in major depressive disorder: a multimodal imaging study. Biol Psychiatry Cogn Neurosci Neuroimaging. (2017) 2:449–56. 10.1016/j.bpsc.2017.03.019 28993818 PMC5630181

[178] Fatemi SH Wong DF Brašić JR Kuwabara H Mathur A Folsom TD. Metabotropic glutamate receptor 5 tracer [18F]-FPEB displays increased binding potential in postcentral gyrus and cerebellum of male individuals with autism: a pilot PET study. Cerebellum Ataxias. (2018) 5:3. 10.1186/s40673-018-0082-1 29449954 PMC5810020

[179] Salabert A-S Fonta C Fontan C Adel D Alonso M Pestourie C Radiolabeling of [18F]-fluoroethylnormemantine and initial *in vivo* evaluation of this innovative PET tracer for imaging the PCP sites of NMDA receptors. Nucl Med Biol. (2015) 42:643–53. 10.1016/j.nucmedbio.2015.04.001 25963911

[180] Martinez D Slifstein M Nabulsi N Grassetti A Urban NBL Perez A. Imaging glutamate homeostasis in cocaine addiction with the metabotropic glutamate receptor 5 positron emission tomography radiotracer [(11)C]ABP688 and magnetic resonance spectroscopy. Biol Psychiatry. (2014) 75:165–71. 10.1016/j.biopsych.2013.06.026 24035345 PMC4106018

[181] Deschwanden A Karolewicz B Feyissa AM Treyer V Ametamey SM Johayem A. Reduced metabotropic glutamate receptor 5 density in major depression determined by [(11)C]ABP688 PET and postmortem study. Am J Psychiatry. (2011) 168:727–34. 10.1176/appi.ajp.2011.09111607 21498461 PMC3129412

[182] Leuzy A Zimmer ER Dubois J Pruessner J Cooperman C Soucy J-P. *In vivo* characterization of metabotropic glutamate receptor type 5 abnormalities in behavioral variant FTD. Brain Struct Funct. (2016) 221:1387–402. 10.1007/s00429-014-0978-3 25596865

[183] Akkus F Mihov Y Treyer V Ametamey SM Johayem A Senn S. Metabotropic glutamate receptor 5 binding in male patients with alcohol use disorder. Transl Psychiatry. (2018) 8:17. 10.1038/s41398-017-0066-6 29317611 PMC5802584

[184] Toyohara J Sakata M Oda K Ishii K Ito K Hiura M. Initial human PET studies of metabotropic glutamate receptor type 1 ligand 11C-ITMM. J Nucl Med Off Publ Soc Nucl Med. (2013) 54:1302–7. 10.2967/jnumed.113.119891 23804329

[185] Sephton SM Herde AM Mu L Keller C Rüdisühli S Auberson Y. Preclinical evaluation and test-retest studies of [(18)F]PSS232, a novel radioligand for targeting metabotropic glutamate receptor 5 (mGlu5). Eur J Nucl Med Mol Imaging. (2015) 42:128–37. 10.1007/s00259-014-2883-7 25139517

[186] Warnock G Sommerauer M Mu L Pla Gonzalez G Geistlich S Treyer V. A first-in-man PET study of [18F]PSS232, a fluorinated ABP688 derivative for imaging metabotropic glutamate receptor subtype 5. Eur J Nucl Med Mol Imaging. (2018) 45:1041–51. 10.1007/s00259-017-3879-x 29177707

[187] Laere KV Koole M Hoon J de Hecken AV Langlois X Andres JI Biodistribution, dosimetry and kinetic modeling of [11C]JNJ-42491293, a PET tracer for the mGluR2 receptor in the human brain. J Nucl Med. (2012) 53(Suppl. 1):355.

[188] Sahai S. Glutamate in the mammalian CNS. Eur Arch Psychiatry Clin Neurosci. (1990) 240:121–33. 10.1007/BF02189982 1981150

[189] Villmann C Strutz N Morth T Hollmann M. Investigation by ion channel domain transplantation of rat glutamate receptor subunits, orphan receptors and a putative NMDA receptor subunit. Eur J Neurosci. (1999) 11:1765–78. 10.1046/j.1460-9568.1999.00594.x 10215929

[190] Paarmann I Frermann D Keller BU Villmann C Breitinger HG Hollmann M. Kinetics and subunit composition of NMDA receptors in respiratory-related neurons. J Neurochem. (2005) 93:812–24. 10.1111/j.1471-4159.2005.03027.x 15857385

[191] Petrenko AB Yamakura T Baba H Shimoji K. The role of N-methyl-D-aspartate (NMDA) receptors in pain: a review. Anesth Analg. (2003) 97:1108–16. 10.1213/01.ANE.0000081061.12235.55 14500166

[192] Dingledine R Borges K Bowie D Traynelis SF. The glutamate receptor ion channels. Pharmacol Rev. (1999) 51:7–61. 10049997

[193] Song XJ Zhao ZQ. Cooperative interaction among the various regulatory sites within the NMDA receptor-channel complex in modulating the evoked responses to noxious thermal stimuli of spinal dorsal horn neurons in the cat. Exp Brain Res. (1998) 120:257–62. 10.1007/s002210050399 9629967

[194] Sattler R Xiong Z Lu WY Hafner M MacDonald JF Tymianski M. Specific coupling of NMDA receptor activation to nitric oxide neurotoxicity by PSD-95 protein. Science. (1999) 284:1845–8. 10.1126/science.284.5421.1845 10364559

[195] Leker RR Shohami E. Cerebral ischemia and trauma-different etiologies yet similar mechanisms: neuroprotective opportunities. Brain Res Brain Res Rev. (2002) 39:55–73. 10.1016/S0165-0173(02)00157-1 12086708

[196] Kalia LV Kalia SK Salter MW. NMDA receptors in clinical neurology: excitatory times ahead. Lancet Neurol. (2008) 7:742–55. 10.1016/S1474-4422(08)70165-0 18635022 PMC3589564

[197] Lau CG Zukin RS. NMDA receptor trafficking in synaptic plasticity and neuropsychiatric disorders. Nat Rev Neurosci. (2007) 8:413–26. 10.1038/nrn2153 17514195

[198] Ponchant M Kamenkaj M Crouzel C Synthesis of 3-[18F]-fluoromethyl-TCP1, A potential tool for pet study of the nmda receptor channel complex. J Label Compd Radiopharm. (1992) 31:955–60. 10.1002/jlcr.2580311115

[199] Ponchant M Crouzel C Kamenka JM Pappata S Synthesis of a new analog of PCP Fluorine-18 3-fluoromethyl-TCP, a potent ligand for the NMDA glutamatergic receptor. J Label Compd Radiopharm. (1993) 32:352–3.

[200] Shiue C-Y Vallabhahosula S Wolf AP Dewey SL Fowler JS Schlyer DJ. Carbon-11 labelled ketamine—Synthesis, distribution in mice and PET studies in baboons. Nucl Med Biol. (1997) 24:145–50. 10.1016/S0969-8051(96)00186-2 9089707

[201] Ametamey SM Bruehlmeier M Kneifel S Kokic M Honer M Arigoni M. PET studies of 18F-memantine in healthy volunteers. Nucl Med Biol. (2002) 29:227–31. 10.1016/S0969-8051(01)00293-1 11823128

[202] Ametamey SM Samnick S Leenders KL Vontobel P Quack G Parsons CG. Fluorine-18 radiolabelling, biodistribution studies and preliminary pet evaluation of a new memantine derivative for imaging the NMDA receptor. J Recept Signal Transduct. (1999) 19:129–41. 10.3109/10799899909036640 10071753

[203] Kiesewetter DO Finn RD Rice KC Monn JA Synthesis of 11C-labeled (±)-5-methyl-10, 11-dihydro-5H-dibenzo[a,d]cyclohepten-5, 10-imine [(±)-[11C]MK801]. Int J Rad Appl Instrum. (1990) 41:139–42. 10.1016/0883-2889(90)90098-2 2158943

[204] Brown DR Wyper DJ Owens J Patterson J Kelly RC Hunter R. 123Iodo-MK-801: a spect agent for imaging the pattern and extent of glutamate (NMDA) receptor activation in Alzheimer's disease. J Psychiatr Res. (1997) 31:605–19. 10.1016/S0022-3956(97)00031-9 9447566

[205] Majo VJ Prabhakaran J Mann JJ Kumar JSD. PET and SPECT tracers for glutamate receptors. Drug Discov Today. (2013) 18:173–84. 10.1016/j.drudis.2012.10.004 23092894

[206] Sobrio F Gilbert G Perrio C Barré L Debruyne D. PET and SPECT imaging of the NMDA receptor system: an overview of radiotracer development. Mini Rev Med Chem. (2010) 10:870–86. 10.2174/138955710791608299 20504276

[207] Stone JM. Imaging the glutamate system in humans: relevance to drug discovery for schizophrenia. Curr Pharm Des. (2009) 15:2594–602. 10.2174/138161209788957438 19689330

[208] McGinnity CJ Hammers A Riaño Barros DA Luthra SK Jones PA Trigg W. Initial evaluation of 18F-GE-179, a putative PET Tracer for activated N-methyl D-aspartate receptors. J Nucl Med Off Publ Soc Nucl Med. (2014) 55:423–30. 10.2967/jnumed.113.130641 24525206

[209] López-Picón F Snellman A Shatillo O Lehtiniemi P Grönroos TJ Marjamäki P. *Ex vivo* tracing of NMDA and GABA-A receptors in rat brain after traumatic brain injury using 18F-GE-179 and 18F-GE-194 autoradiography. J Nucl Med Off Publ Soc Nucl Med. (2016) 57:1442–7. 10.2967/jnumed.115.167403 27199360

[210] Zhou W Bao W Jiang D Kong Y Hua F Lu X. [18F]-GE-179 positron emission tomography (PET) tracer for N-methyl-d-aspartate receptors: One-pot synthesis and preliminary micro-PET study in a rat model of MCAO. Nucl Med Biol. (2018) 61:45–55. 10.1016/j.nucmedbio.2018.04.002 29747036

[211] Schoenberger M Schroeder FA Placzek MS Carter RL Rosen BR Hooker JM *In vivo* [18F]GE-179 brain signal does not show NMDA-specific modulation with drug challenges in rodents and nonhuman primates. ACS Chem Neurosci. (2018) 9:298–305. 10.1021/acschemneuro.7b00327 29050469 PMC5894869

[212] Salabert A-S Mora-Ramirez E Beaurain M Alonso M Fontan C Tahar HB. Evaluation of [18F]FNM biodistribution and dosimetry based on whole-body PET imaging of rats. Nucl Med Biol. (2018) 59:1–8. 10.1016/j.nucmedbio.2017.12.003 29413751

[213] van der Aart J Golla SSV van der Pluijm M Schwarte LA Schuit RC Klein PJ. First in human evaluation of [18F]PK-209, a PET ligand for the ion channel binding site of NMDA receptors. EJNMMI Res. (2018) 8:69. 10.1186/s13550-018-0424-2 30054846 PMC6063804

[214] Roger G Dollé F De Bruin B Liu X Besret L Bramoullé Y. Radiosynthesis and pharmacological evaluation of [11C]EMD-95885: a high affinity ligand for NR2B-containing NMDA receptors. Bioorg Med Chem. (2004) 12:3229–37. 10.1016/j.bmc.2004.03.065 15158791

[215] Labas R Gilbert G Nicole O Dhilly M Abbas A Tirel O. Synthesis, evaluation and metabolic studies of radiotracers containing a 4-(4-[18F]-fluorobenzyl)piperidin-1-yl moiety for the PET imaging of NR2B NMDA receptors. Eur J Med Chem. (2011) 46:2295–309. 10.1016/j.ejmech.2011.03.013 21453995

[216] Yamasaki T Maeda J Fujinaga M Nagai Y Hatori A Yui J. PET brain kinetics studies of (11)C-ITMM and (11)C-ITDM,radioprobes for metabotropic glutamate receptor type 1, in a nonhuman primate. Am J Nucl Med Mol Imaging. (2014) 4:260–9. 24795840 PMC3999406

[217] Pillai RLI Tipre DN. Metabotropic glutamate receptor 5 – a promising target in drug development and neuroimaging. Eur J Nucl Med Mol Imaging. (2016) 43:1151–70. 10.1007/s00259-015-3301-5 26743895

[218] Sullivan EV Adalsteinsson E Sood R Mayer D Bell R McBride W. Longitudinal brain magnetic resonance imaging study of the alcohol-preferring rat. Part I: adult brain growth. Alcohol Clin Exp Res. (2006) 30:1234–47. 10.1111/j.1530-0277.2006.00145.x 16792572

[219] Sullivan JM Lim K Labaree D Lin S McCarthy TJ Seibyl JP. Kinetic analysis of the metabotropic glutamate subtype 5 tracer [18F]FPEB in bolus and bolus-plus-constant-infusion studies in humans. J Cereb Blood Flow Metab. (2013) 33:532. 10.1038/jcbfm.2012.195 23250105 PMC3618388

[220] Kang Y Henchcliffe C Verma A Vallabhajosula S He B Kothari PJ. 18F-FPEB PET/CT shows mGluR5 upregulation in Parkinson's disease. J Neuroimaging Off J Am Soc Neuroimaging. (2019) 29:97–103. 10.1111/jon.12563 30230118

[221] de Laat B Weerasekera A Leurquin-Sterk G Gsell W Bormans G Himmelreich U. Effects of alcohol exposure on the glutamatergic system: a combined longitudinal 18 F-FPEB and 1 H-MRS study in rats. Addict Biol. (2018) 24:696–706. 10.1111/adb.12635 29790622

[222] Holmes SE Girgenti MJ Davis MT Pietrzak RH DellaGioia N Nabulsi N. Altered metabotropic glutamate receptor 5 markers in PTSD: *in vivo* and postmortem evidence. Proc Natl Acad Sci USA. (2017) 114:8390–5. 10.1073/pnas.1701749114 28716937 PMC5547601

[223] Ametamey SM Treyer V Streffer J Wyss MT Schmidt M Blagoev M. Human PET studies of metabotropic glutamate receptor subtype 5 with 11C-ABP688. J Nucl Med Off Publ Soc Nucl Med. (2007) 48:247–52. 17268022

[224] Burger C Deschwanden A Ametamey S Johayem A Mancosu B Wyss M. Evaluation of a bolus/infusion protocol for 11C-ABP688, a PET tracer for mGluR5. Nucl Med Biol. (2010) 37:845–51. 10.1016/j.nucmedbio.2010.04.107 20870160

[225] DuBois JM Rousset OG Guiot M-C Hall JA Reader AJ Soucy J-P Metabotropic Glutamate Receptor Type 5 (mGluR5) cortical abnormalities in focal cortical dysplasia identified *in vivo* with [11C]ABP688 Positron-Emission Tomography (PET) imaging. Cereb Cortex N Y N. (2016) 26:4170–9. 10.1093/cercor/bhw249 PMC5066831 27578494

[226] Akkus F Treyer V Ametamey SM Johayem A Buck A Hasler G. Metabotropic glutamate receptor 5 neuroimaging in schizophrenia. Schizophr Res. (2017) 183:95–101. 10.1016/j.schres.2016.11.008 27847228

[227] Kim J-H Joo Y-H Son Y-D Kim J-H Kim Y-K Kim H-K. *In vivo* metabotropic glutamate receptor 5 availability-associated functional connectivity alterations in drug-naïve young adults with major depression. Eur Neuropsychopharmacol. (2018) 29:278–90. 10.1016/j.euroneuro.2018.12.001 30553696

[228] Esterlis I DellaGioia N Pietrzak RH Matuskey D Nabulsi N Abdallah CG Ketamine-induced reduction in mGluR5 availability is associated with an antidepressant response: an [11C]ABP688 and PET imaging study in depression. Mol Psychiatry. (2018) 23:824–32. 10.1038/mp.2017.58 28397841 PMC5636649

[229] DeLorenzo C DellaGioia N Bloch M Sanacora G Nabulsi N Abdallah C. *In vivo* ketamine-induced changes in [^11^C]ABP688 binding to metabotropic glutamate receptor subtype 5. Biol Psychiatry. (2015) 77:266–75. 10.1016/j.biopsych.2014.06.024 25156701 PMC4277907

[230] Kosten L Verhaeghe J Wyffels L Stroobants S Staelens S Acute ketamine infusion in rat does not affect *in vivo* [11C]ABP688 binding to metabotropic glutamate receptor subtype 5. Mol Imaging. (2018) 17:1536012118788636 10.1177/1536012118788636 30213221 PMC6144515

[231] O'Gorman Tuura R Warnock G Ametamey S Treyer V Noeske R Buck A. Imaging glutamate redistribution after acute N-acetylcysteine administration: a simultaneous PET/MR study. NeuroImage. (2019) 184:826–33. 10.1016/j.neuroimage.2018.10.017 30296554

[232] Müller Herde A Boss SD He Y Schibli R Mu L Ametamey SM Ketamine and ceftriaxone-induced alterations in glutamate levels do not impact the specific binding of metabotropic glutamate receptor subtype 5 radioligand [18F]PSS232 in the rat brain. Pharm Basel Switz. (2018) 11:E83 10.3390/ph11030083 PMC6161118 30158438

[233] Leurquin-Sterk G Celen S Laere KV Koole M Bormans G Langlois X What we observe *in vivo* is not always what we see *in vitro*: development and validation of 11C-JNJ-42491293, a novel radioligand for mGluR2. J Nucl Med. (2017) 58:110–6. 10.2967/jnumed.116.176628 27469358

[234] Roy R Niccolini F Pagano G Politis M. Cholinergic imaging in dementia spectrum disorders. Eur J Nucl Med Mol Imaging. (2016) 43:1376–86. 10.1007/s00259-016-3349-x 26984612 PMC4865532

[235] Perry EK Gibson PH Blessed G Perry RH Tomlinson BE. Neurotransmitter enzyme abnormalities in senile dementia: choline acetyltransferase and glutamic acid decarboxylase activities in necropsy brain tissue. J Neurol Sci. (1977) 34:247–65. 10.1016/0022-510X(77)90073-9 144789

[236] Davis KL Mohs RC Marin D Purohit DP Perl DP Lantz M. Cholinergic markers in elderly patients with early signs of Alzheimer disease. JAMA. (1999) 281:1401–6. 10.1001/jama.281.15.1401 10217056

[237] Kuhl DE Koeppe RA Minoshima S Snyder SE Ficaro EP Foster NL. *In vivo* mapping of cerebral acetylcholinesterase activity in aging and Alzheimer's disease. Neurology. (1999) 52:691–9. 10.1212/WNL.52.4.691 10078712

[238] Shinotoh H Namba H Yamaguchi M Fukushi K Nagatsuka S Iyo M. Positron emission tomographic measurement of acetylcholinesterase activity reveals differential loss of ascending cholinergic systems in Parkinson's disease and progressive supranuclear palsy. Ann Neurol. (1999) 46:62–9. 10401781

[239] Bohnen NI Kaufer DI Ivanco LS Lopresti B Koeppe RA Davis JG. Cortical cholinergic function is more severely affected in parkinsonian dementia than in Alzheimer disease: an *in vivo* positron emission tomographic study. Arch Neurol. (2003) 60:1745–8. 10.1001/archneur.60.12.1745 14676050

[240] Bohnen NI Kaufer DI Hendrickson R Ivanco LS Lopresti B Davis JG. Cognitive correlates of alterations in acetylcholinesterase in Alzheimer's disease. Neurosci Lett. (2005) 380:127–32. 10.1016/j.neulet.2005.01.031 15854764

[241] Hilker R Thomas AV Klein JC Weisenbach S Kalbe E Burghaus L. Dementia in Parkinson disease: functional imaging of cholinergic and dopaminergic pathways. Neurology. (2005) 65:1716–22. 10.1212/01.wnl.0000191154.78131.f6 16344512

[242] Eggers C Herholz K Kalbe E Heiss W-D. Cortical acetylcholine esterase activity and ApoE4-genotype in Alzheimer disease. Neurosci Lett. (2006) 408:46–50. 10.1016/j.neulet.2006.08.061 16996687

[243] Irie T Fukushi K Akimoto Y Tamagami H Nozaki T. Design and evaluation of radioactive acetylcholine analogs for mapping brain acetylcholinesterase (AchE) *in vivo*. Nucl Med Biol. (1994) 21:801–8. 10.1016/0969-8051(94)90159-7 9234329

[244] Koeppe RA Frey KA Snyder SE Meyer P Kilbourn MR Kuhl DE Kinetic modeling of N-[11C]Methylpiperidin-4-yl propionate: alternatives for analysis of an irreversible positron emission tomography tracer for measurement of acetylcholinesterase activity in human brain. J Cereb Blood Flow Metab. (1999) 19:1150–63. 10.1097/00004647-199910000-00012 10532640

[245] Mazere J Meissner WG Sibon I Lamare F Tison F Allard M. [(123)I]-IBVM SPECT imaging of cholinergic systems in multiple system atrophy: a specific alteration of the ponto-thalamic cholinergic pathways (Ch5-Ch6). NeuroImage Clin. (2013) 3:212–7. 10.1016/j.nicl.2013.07.012 24179865 PMC3791287

[246] Nejad-Davarani S Koeppe RA Albin RL Frey KA Müller MLTM Bohnen NI Quantification of brain cholinergic denervation in dementia with Lewy bodies using PET imaging with [18F]-FEOBV. Mol Psychiatry. (2018) 24:322–7. 10.1038/s41380-018-0130-5 30082840 PMC6363916

[247] Petrou M Frey KA Kilbourn MR Scott PJH Raffel DM Bohnen NI. *In vivo* imaging of human cholinergic nerve terminals with (-)-5-(18)F-fluoroethoxybenzovesamicol: biodistribution, dosimetry, and tracer kinetic analyses. J Nucl Med Off Publ Soc Nucl Med. (2014) 55:396–404. 10.2967/jnumed.113.124792 24481024

[248] Nordberg A Hartvig P Lilja A Viitanen M Amberla K Lundqvist H. Decreased uptake and binding of 11C-nicotine in brain of Alzheimer patients as visualized by positron emission tomography. J Neural Transm Park Dis Dement Sect. (1990) 2:215–24. 10.1007/BF02257652 2257061

[249] Nordberg A Lundqvist H Hartvig P Lilja A Långström B. Kinetic analysis of regional (S)(-)11C-nicotine binding in normal and Alzheimer brains–*in vivo* assessment using positron emission tomography. Alzheimer Dis Assoc Disord. (1995) 9:21–7. 10.1097/00002093-199505000-00006 7605618

[250] Kadir A Almkvist O Wall A Långström B Nordberg A. PET imaging of cortical 11C-nicotine binding correlates with the cognitive function of attention in Alzheimer's disease. Psychopharmacology. (2006) 188:509–20. 10.1007/s00213-006-0447-7 16832659

[251] Sabbagh MN Shah F Reid RT Sue L Connor DJ Peterson LKN. Pathologic and nicotinic receptor binding differences between mild cognitive impairment, Alzheimer disease, and normal aging. Arch Neurol. (2006) 63:1771–6. 10.1001/archneur.63.12.1771 17172618

[252] O'Brien JT Colloby SJ Pakrasi S Perry EK Pimlott SL Wyper DJ. Alpha4beta2 nicotinic receptor status in Alzheimer's disease using 123I-5IA-85380 single-photon-emission computed tomography. J Neurol Neurosurg Psychiatry. (2007) 78:356–62. 10.1136/jnnp.2006.108209 17135460 PMC2077777

[253] Sabri O Kendziorra K Wolf H Gertz H-J Brust P. Acetylcholine receptors in dementia and mild cognitive impairment. Eur J Nucl Med Mol Imaging. (2008) 35 (Suppl. 1):S30–45. 10.1007/s00259-007-0701-1 18228017

[254] Okada H Ouchi Y Ogawa M Futatsubashi M Saito Y Yoshikawa E. Alterations in α4β2 nicotinic receptors in cognitive decline in Alzheimer's aetiopathology. Brain J Neurol. (2013) 136(Pt 10):3004–17. 10.1093/brain/awt195 23975517

[255] Hu M Waring JF Gopalakrishnan M Li J. Role of GSK-3beta activation and alpha7 nAChRs in Abeta(1-42)-induced tau phosphorylation in PC12 cells. J Neurochem. (2008) 106:1371–7. 10.1111/j.1471-4159.2008.05483.x 18485099

[256] Dziewczapolski G Glogowski CM Masliah E Heinemann SF. Deletion of the alpha 7 nicotinic acetylcholine receptor gene improves cognitive deficits and synaptic pathology in a mouse model of Alzheimer's disease. J Neurosci Off J Soc Neurosci. (2009) 29:8805–15. 10.1523/JNEUROSCI.6159-08.2009 19587288 PMC2753494

[257] Asahina M Suhara T Shinotoh H Inoue O Suzuki K Hattori T. Brain muscarinic receptors in progressive supranuclear palsy and Parkinson's disease: a positron emission tomographic study. J Neurol Neurosurg Psychiatry. (1998) 65:155–63. 10.1136/jnnp.65.2.155 9703164 PMC2170218

[258] Colloby SJ Pakrasi S Firbank MJ Perry EK Piggott MA Owens J. *In vivo* SPECT imaging of muscarinic acetylcholine receptors using (R,R) 123I-QNB in dementia with Lewy bodies and Parkinson's disease dementia. NeuroImage. (2006) 33:423–9. 10.1016/j.neuroimage.2006.07.026 16959499

[259] Kadir A Darreh-Shori T Almkvist O Wall A Grut M Strandberg B. PET imaging of the *in vivo* brain acetylcholinesterase activity and nicotine binding in galantamine-treated patients with AD. Neurobiol Aging. (2008) 29:1204–17. 10.1016/j.neurobiolaging.2007.02.020 17379359

[260] Otto CA Mulholland GK Perry SE Combs R Sherman PS Fisher SJ. *In vitro* and *ex vivo* evaluation of cyclic aminoalkyl benzilates as potential emission tomography ligands for the muscarinic receptor. Int J Rad Appl Instrum B. (1989) 16:51–5. 10.1016/0883-2897(89)90215-8 2785511

[261] Horti AG Koren AO Lee KS Mukhin AG Vaupel DB Kimes AS. Radiosynthesis and preliminary evaluation of 5-[123/125I]iodo-3-(2(S)-azetidinylmethoxy)pyridine: a radioligand for nicotinic acetylcholine receptors. Nucl Med Biol. (1999) 26:175–82. 10.1016/S0969-8051(98)00086-9 10100216

[262] Pakrasi S Colloby SJ Firbank MJ Perry EK Wyper DJ Owens J. Muscarinic acetylcholine receptor status in Alzheimer's disease assessed using (R, R) 123I-QNB SPECT. J Neurol. (2007) 254:907–13. 10.1007/s00415-006-0473-8 17361343

[263] Shimada H Hirano S Shinotoh H Aotsuka A Sato K Tanaka N. Mapping of brain acetylcholinesterase alterations in Lewy body disease by PET. Neurology. (2009) 73:273–8. 10.1212/WNL.0b013e3181ab2b58 19474411

[264] Aghourian M Legault-Denis C Soucy J-P Rosa-Neto P Gauthier S Kostikov A Quantification of brain cholinergic denervation in Alzheimer's disease using PET imaging with [18F]-FEOBV. Mol Psychiatry. (2017) 22:1531–8. 10.1038/mp.2017.183 28894304

[265] Yoshida T Kuwabara Y Ichiya Y Sasaki M Fukumura T Ichimiya A. Cerebral muscarinic acetylcholinergic receptor measurement in Alzheimer's disease patients on 11C-N-methyl-4-piperidyl benzilate–comparison with cerebral blood flow and cerebral glucose metabolism. Ann Nucl Med. (1998) 12:35–42. 10.1007/BF03165414 9559960

[266] Koren AO Horti AG Mukhin AG Gündisch D Kimes AS Dannals RF. 2-, 5-, and 6-Halo-3-(2(S)-azetidinylmethoxy)pyridines: synthesis, affinity for nicotinic acetylcholine receptors, and molecular modeling. J Med Chem. (1998) 41:3690–8. 10.1021/jm980170a 9733494

[267] Sihver W Fasth KJ Ogren M Bivehed H Bergström M Nordberg A. *In vitro* evaluation of 11C-labeled (S)-nicotine, (S)-3-methyl-5-(1-methyl-2-pyrrolidinyl)isoxazole, and (R,S)-1-methyl-2-(3-pyridyl)azetidine as nicotinic receptor ligands for positron emission tomography studies. J Neurochem. (1998) 71:1750–60. 10.1046/j.1471-4159.1998.71041750.x 9751211

[268] Jung YW Frey KA Mulholland GK del Rosario R Sherman PS Raffel DM. Vesamicol receptor mapping of brain cholinergic neurons with radioiodine-labeled positional isomers of benzovesamicol. J Med Chem. (1996) 39:3331–42. 10.1021/jm9507486 8765517

[269] Horti AG Gao Y Kuwabara H Wang Y Abazyan S Yasuda RP. 18F-ASEM, a radiolabeled antagonist for imaging the α7-nicotinic acetylcholine receptor with PET. J Nucl Med. (2014) 55:672–7. 10.2967/jnumed.113.132068 24556591 PMC4112566

[270] Wong DF Kuwabara H Horti AG Roberts JM Nandi A Cascella N Brain PET imaging of α7-nAChR with [18F]ASEM: reproducibility, occupancy, receptor density, and changes in schizophrenia. Int J Neuropsychopharmacol. (2018) 21:656–67. 10.1101/245118 29522184 PMC6030963

[271] Lin S-F Bois F Holden D Nabulsi N Pracitto R Gao H. The search for a subtype-selective PET imaging agent for the GABAA receptor complex: evaluation of the radiotracer [11C]ADO in nonhuman primates. Mol Imaging. (2017) 16:1536012117731258. 10.1177/1536012117731258 28929924 PMC5912275

[272] Stephens DN King SL Lambert JJ Belelli D Duka T. GABAA receptor subtype involvement in addictive behaviour. Genes Brain Behav. (2017) 16:149–84. 10.1111/gbb.12321 27539865

[273] Rodnick ME Hockley BG Sherman P Quesada C Battle MR Jackson A. Novel fluorine-18 PET radiotracers based on flumazenil for GABAA imaging in the brain. Nucl Med Biol. (2013) 40:901–5. 10.1016/j.nucmedbio.2013.06.004 23890694 PMC3769461

[274] Baldwin RM Horti AG Bremner JD Stratton MD Dannals RF Ravert HT. Synthesis and PET imaging of the benzodiazepine receptor tracer [N-methyl-11C]iomazenil. Nucl Med Biol. (1995) 22:659–65. 10.1016/0969-8051(94)00139-B 7581177

[275] Andersson JD Halldin C PET radioligands targeting the brain GABAA /benzodiazepine receptor complex. J Label Compd Radiopharm. (2013) 56:196–206. 10.1002/jlcr.3008 24285326

[276] Dobbs FR Banks W Fleishaker JC Valentine AD Kinsey BM Franceschini MP. Studies with [11C]alprazolam: an agonist for the benzodiazepine receptor. Nucl Med Biol. (1995) 22:459–66. 10.1016/0969-8051(94)00131-3 7550022

[277] Persson A Ehrin E Eriksson L Farde L Hedström CG Litton JE Imaging of [11C]-labelled Ro 15-1788 binding to benzodiazepine receptors in the human brain by positron emission tomography. J Psychiatr Res. (1985) 19:609–22. 10.1016/0022-3956(85)90080-9 3001301

[278] Pike VW Halldin C Crouzel C Barré L Nutt DJ Osman S. Radioligands for PET studies of central benzodiazepine receptors and PK (peripheral benzodiazepine) binding sites–current status. Nucl Med Biol. (1993) 20:503–25. 10.1016/0969-8051(93)90082-6 8389223

[279] Abadie P Rioux P Scatton B Zarifian E Barré L Patat A. Central benzodiazepine receptor occupancy by zolpidem in the human brain as assessed by positron emission tomography. Eur J Pharmacol. (1996) 295:35–44. 10.1016/0014-2999(95)00633-8 8925872

[280] Frankle WG Cho RY Narendran R Mason NS Vora S Litschge M. Tiagabine increases [^11^C]flumazenil binding in cortical brain regions in healthy control subjects. Neuropsychopharmacology. (2009) 34:624–33. 10.1038/npp.2008.104 18615011 PMC2754778

[281] Savic I Pauli S Thorell JO Blomqvist G. *In vivo* demonstration of altered benzodiazepine receptor density in patients with generalised epilepsy. J Neurol Neurosurg Psychiatry. (1994) 57:797–804. 10.1136/jnnp.57.7.797 8021664 PMC1073018

[282] la Fougère C Rominger A Förster S Geisler J Bartenstein P PET and SPECT in epilepsy: a critical review. Epilepsy Behav. (2009) 15:50–5. 10.1016/j.yebeh.2009.02.025 19236949

[283] Savic I Roland P Sedvall G Persson A Pauli S Widen L *In-vivo* demonstration of reduced benzodiazepine receptor binding in human epileptic FOCI. Lancet. (1988) 332:863–6. 10.1016/S0140-6736(88)92468-3 2902315

[284] Egerton A Modinos G Ferrera D McGuire P. Neuroimaging studies of GABA in schizophrenia: a systematic review with meta-analysis. Transl Psychiatry. (2017) 7:e1147. 10.1038/tp.2017.124 28585933 PMC5537645

[285] Heiss WD Sobesky J Smekal UV Kracht LW Lehnhardt FG Thiel A. Probability of cortical infarction predicted by flumazenil binding and diffusion-weighted imaging signal intensity. Stroke. (2004) 35:1892–8. 10.1161/01.STR.0000134746.93535.9b 15218157

[286] Jucaite A Cselényi Z Lappalainen J McCarthy DJ Lee C-M Nyberg S. GABAA receptor occupancy by subtype selective GABAAα2,3 modulators: PET studies in humans. Psychopharmacology. (2017) 234:707–16. 10.1007/s00213-016-4506-4 28013354 PMC5263201

[287] Taguchi Y Takashima S Noguchi K Tanaka K. Findings of 123I-iomazenil SPECT during and after stroke-like episodes in a patient with MELAS. Clin Nucl Med. (2014) 39:e334–5. 10.1097/RLU.0b013e318299610f 24097000

[288] Fujitani S Matsuda K Nakamura F Baba K Usui N Tottori T. Statistical parametric mapping of interictal 123I-iomazenil SPECT in temporal lobe epilepsy surgery. Epilepsy Res. (2013) 106:173–80. 10.1016/j.eplepsyres.2013.03.008 23582957

[289] Nagamitsu S Sakurai R Matsuoka M Chiba H Ozono S Tanigawa H. Altered SPECT (123)I-iomazenil binding in the cingulate cortex of children with anorexia nervosa. Front Psychiatry. (2016) 7:16. 10.3389/fpsyt.2016.00016 26909048 PMC4754452

[290] Lingford-Hughes A Hume SP Feeney A Hirani E Osman S Cunningham VJ Imaging the GABA-benzodiazepine receptor subtype containing the alpha5-subunit *in vivo* with [11C]Ro15 4513 positron emission tomography. J Cereb Blood Flow Metab Off J Int Soc Cereb Blood Flow Metab. (2002) 22:878–89. 10.1097/00004647-200207000-00013 12142573

[291] Maeda J Suhara T Kawabe K Okauchi T Obayashi S Hojo J. Visualization of alpha5 subunit of GABAA/benzodiazepine receptor by 11C Ro15-4513 using positron emission tomography. Synap N Y N. (2003) 47:200–8. 10.1002/syn.10169 12494402

[292] Lingford-Hughes A Reid AG Myers J Feeney A Hammers A Taylor LG A [11C]Ro15 4513 PET study suggests that alcohol dependence in man is associated with reduced α5 benzodiazepine receptors in limbic regions. J Psychopharmacol Oxf Engl. (2012) 26:273–81. 10.1177/0269881110379509 20870689

[293] Asai Y Takano A Ito H Okubo Y Matsuura M Otsuka A GABAA/Benzodiazepine receptor binding in patients with schizophrenia using [11C]Ro15-4513, a radioligand with relatively high affinity for alpha5 subunit. Schizophr Res. (2008) 99:333–40. 10.1016/j.schres.2007.10.014 18042347

[294] Dedeurwaerdere S Gregoire M-C Vivash L Roselt P Binns D Fookes C. *In-vivo* imaging characteristics of two fluorinated flumazenil radiotracers in the rat. Eur J Nucl Med Mol Imaging. (2009) 36:958–65. 10.1007/s00259-009-1066-4 19205698

[295] Sieghart W. Structure and pharmacology of gamma-aminobutyric acidA receptor subtypes. Pharmacol Rev. (1995) 47:181–234. 7568326

[296] Laruelle M Abi-Dargham A al-Tikriti MS Baldwin RM Zea-Ponce Y Zoghbi SS SPECT quantification of [123I]iomazenil binding to benzodiazepine receptors in nonhuman primates: II. Equilibrium analysis of constant infusion experiments and correlation with *in vitro* parameters. J Cereb Blood Flow Metab Off J Int Soc Cereb Blood Flow Metab. (1994) 14:453–65. 10.1038/jcbfm.1994.56 8163587

[297] Mendez MA Horder J Myers J Coghlan S Stokes P Erritzoe D The brain GABA-benzodiazepine receptor alpha-5 subtype in autism spectrum disorder: a pilot [11C]Ro15-4513 positron emission tomography study. Neuropharmacology. (2013) 68:195–201. 10.1016/j.neuropharm.2012.04.008 22546616 PMC4489617

[298] Vivash L Gregoire M-C Lau EW Ware RE Binns D Roselt P. 18F-flumazenil: a γ-aminobutyric acid A-specific PET radiotracer for the localization of drug-resistant temporal lobe epilepsy. J Nucl Med Off Publ Soc Nucl Med. (2013) 54:1270–7. 10.2967/jnumed.112.107359 23857513

[299] Hodolic M Topakian R Pichler R. (18)F-fluorodeoxyglucose and (18)F-flumazenil positron emission tomography in patients with refractory epilepsy. Radiol Oncol. (2016) 50:247–53. 10.1515/raon-2016-0032 27679539 PMC5024661

[300] Paterson LM Kornum BR Nutt DJ Pike VW Knudsen GM. 5-HT radioligands for human brain imaging with PET and SPECT. Med Res Rev. (2013) 33:54–111. 10.1002/med.20245 21674551 PMC4188513

[301] King MV Marsden CA Fone KCF. A role for the 5-HT(1A), 5-HT4 and 5-HT6 receptors in learning and memory. Trends Pharmacol Sci. (2008) 29:482–92. 10.1016/j.tips.2008.07.001 19086256

[302] Akimova E Lanzenberger R Kasper S. The serotonin-1A receptor in anxiety disorders. Biol Psychiatry. (2009) 66:627–35. 10.1016/j.biopsych.2009.03.012 19423077

[303] Nash JR Sargent PA Rabiner EA Hood SD Argyropoulos SV Potokar JP. Serotonin 5-HT1A receptor binding in people with panic disorder: positron emission tomography study. Br J Psychiatry J Ment Sci. (2008) 193:229–34. 10.1192/bjp.bp.107.041186 18757983

[304] Bailer UF Frank GK Henry SE Price JC Meltzer CC Mathis CA. Exaggerated 5-HT1A but normal 5-HT2A receptor activity in individuals ill with anorexia nervosa. Biol Psychiatry. (2007) 61:1090–9. 10.1016/j.biopsych.2006.07.018 17241616

[305] Kumar JSD Mann JJ. PET tracers for 5-HT(1A) receptors and uses thereof. Drug Discov Today. (2007) 12:748–56. 10.1016/j.drudis.2007.07.008 17826688

[306] Doder M Rabiner EA Turjanski N Lees AJ Brooks DJ 11C-WAY 100635 PET study. Tremor in Parkinson's disease and serotonergic dysfunction: an 11C-WAY 100635 PET study. Neurology. (2003) 60:601–5. 10.1212/01.WNL.0000031424.51127.2B 12601099

[307] Merlet I Ostrowsky K Costes N Ryvlin P Isnard J Faillenot I 5-HT1A receptor binding and intracerebral activity in temporal lobe epilepsy: an [18F]MPPF-PET study. Brain J Neurol. (2004) 127(Pt 4):900–13. 10.1093/brain/awh109 14985263

[308] Truchot L Costes SN Zimmer L Laurent B Le Bars D Thomas-Antérion C. Up-regulation of hippocampal serotonin metabolism in mild cognitive impairment. Neurology. (2007) 69:1012–7. 10.1212/01.wnl.0000271377.52421.4a 17785670

[309] Lothe A Merlet I Demarquay G Costes N Ryvlin P Mauguière F. Interictal Brain 5-HT1A receptors binding in migraine without Aura: a 18F-MPPF-PET study. Cephalalgia. (2008) 28:1282–91. 10.1111/j.1468-2982.2008.01677.x 18727636

[310] Demarquay G Lothe A Royet J Costes N Mick G Mauguière F. Brainstem changes in 5-HT1A receptor availability during migraine attack. Cephalalgia. (2011) 31:84–94. 10.1177/0333102410385581 21036859

[311] Lothe A Didelot A Hammers A Costes N Saoud M Gilliam F Comorbidity between temporal lobe epilepsy and depression: a [18F]MPPF PET study. Brain J Neurol. (2008) 131(Pt 10):2765–82. 10.1093/brain/awn194 PMC6276903 18765418

[312] Carson RE Lang L Watabe H Der MG Adams HR Jagoda E. PET evaluation of [(18)F]FCWAY, an analog of the 5-HT(1A) receptor antagonist, WAY-100635. Nucl Med Biol. (2000) 27:493–7. 10.1016/S0969-8051(00)00118-9 10962257

[313] Toczek MT Carson RE Lang L Ma Y Spanaki MV Der MG. PET imaging of 5-HT1A receptor binding in patients with temporal lobe epilepsy. Neurology. (2003) 60:749–56. 10.1212/01.WNL.0000049930.93113.20 12629228

[314] Giovacchini G Toczek MT Bonwetsch R Bagic A Lang L Fraser C. 5-HT 1A receptors are reduced in temporal lobe epilepsy after partial-volume correction. J Nucl Med Off Publ Soc Nucl Med. (2005) 46:1128–35. 16000281 PMC1454475

[315] Hasler G Bonwetsch R Giovacchini G Toczek MT Bagic A Luckenbaugh DA. 5-HT1A receptor binding in temporal lobe epilepsy patients with and without major depression. Biol Psychiatry. (2007) 62:1258–64. 10.1016/j.biopsych.2007.02.015 17588547 PMC2170875

[316] Neumeister A Bain E Nugent AC Carson RE Bonne O Luckenbaugh DA. Reduced serotonin type 1A receptor binding in panic disorder. J Neurosci Off J Soc Neurosci. (2004) 24:589–91. 10.1523/JNEUROSCI.4921-03.2004 14736842 PMC6729263

[317] Bonne O Bain E Neumeister A Nugent AC Vythilingam M Carson RE. No change in serotonin type 1A receptor binding in patients with posttraumatic stress disorder. Am J Psychiatry. (2005) 162:383–5. 10.1176/appi.ajp.162.2.383 15677606

[318] Choi JY Lyoo CH Kim JS Kim KM Kang JH Choi S-H 18F-Mefway PET imaging of serotonin 1A receptors in humans: a comparison with 18F-FCWAY. PLoS ONE. (2015) 10:e0121342 10.1371/journal.pone.0121342 25830772 PMC4382022

[319] Milak MS DeLorenzo C Zanderigo F Prabhakaran J Kumar JSD Majo VJ. *In vivo* quantification of human serotonin 1A receptor using 11C-CUMI-101, an agonist PET radiotracer. J Nucl Med Off Publ Soc Nucl Med. (2010) 51:1892–900. 10.2967/jnumed.110.076257 21098796 PMC3856257

[320] Travis MJ Busatto GF Pilowsky LS Mulligan R Acton PD Gacinovic S. 5-HT2A receptor blockade in patients with schizophrenia treated with risperidone or clozapine. A SPET study using the novel 5-HT2A ligand 123I-5-I-R-91150. Br J Psychiatry J Ment Sci. (1998) 173:236–41. 10.1192/bjp.173.3.236 9926100

[321] Jones HM Travis MJ Mulligan R Bressan RA Visvikis D Gacinovic S. *In vivo* 5-HT2A receptor blockade by quetiapine: an R91150 single photon emission tomography study. Psychopharmacology. (2001) 157:60–6. 10.1007/s002130100761 11512044

[322] Versijpt J Van Laere KJ Dumont F Decoo D Vandecapelle M Santens P. Imaging of the 5-HT2A system: age-, gender-, and Alzheimer's disease-related findings. Neurobiol Aging. (2003) 24:553–61. 10.1016/S0197-4580(02)00137-9 12714112

[323] van Heeringen C Audenaert K Van Laere K Dumont F Slegers G Mertens J. Prefrontal 5-HT2a receptor binding index, hopelessness and personality characteristics in attempted suicide. J Affect Disord. (2003) 74:149–58. 10.1016/S0165-0327(01)00482-7 12706516

[324] Goethals I Vervaet M Audenaert K Jacobs F Ham H Van de Wiele C. Differences of cortical 5-HT2A receptor binding index with SPECT in subtypes of anorexia nervosa: relationship with personality traits? J Psychiatr Res. (2007) 41:455–8. 10.1016/j.jpsychires.2005.04.002 15925385

[325] Sheline YI Mintun MA Barch DM Wilkins C Snyder AZ Moerlein SM Decreased hippocampal 5-HT(2A) receptor binding in older depressed patients using [18F]altanserin positron emission tomography. Neuropsychopharmacol Off Publ Am Coll Neuropsychopharmacol. (2004) 29:2235–41. 10.1038/sj.npp.1300555 15367923

[326] Meltzer CC Price JC Mathis CA Greer PJ Cantwell MN Houck PR. PET imaging of serotonin type 2A receptors in late-life neuropsychiatric disorders. Am J Psychiatry. (1999) 156:1871–8. 10588399 10.1176/ajp.156.12.1871

[327] Haugbøl S Pinborg LH Regeur L Hansen ES Bolwig TG Nielsen FA. Cerebral 5-HT2A receptor binding is increased in patients with Tourette's syndrome. Int J Neuropsychopharmacol. (2007) 10:245–52. 10.1017/S1461145706006559 16945163

[328] Erritzoe D Rasmussen H Kristiansen KT Frokjaer VG Haugbol S Pinborg L. Cortical and subcortical 5-HT2A receptor binding in neuroleptic-naive first-episode schizophrenic patients. Neuropsychopharmacol Off Publ Am Coll Neuropsychopharmacol. (2008) 33:2435–41. 10.1038/sj.npp.1301656 18288096

[329] Frank GK Kaye WH Meltzer CC Price JC Greer P McConaha C. Reduced 5-HT2A receptor binding after recovery from anorexia nervosa. Biol Psychiatry. (2002) 52:896–906. 10.1016/S0006-3223(02)01378-1 12399143

[330] Adams KH Hansen ES Pinborg LH Hasselbalch SG Svarer C Holm S. Patients with obsessive-compulsive disorder have increased 5-HT2A receptor binding in the caudate nuclei. Int J Neuropsychopharmacol. (2005) 8:391–401. 10.1017/S1461145705005055 15801987

[331] Erlandsson K Sivananthan T Lui D Spezzi A Townsend CE Mu S. Measuring SSRI occupancy of SERT using the novel tracer [123I]ADAM: a SPECT validation study. Eur J Nucl Med Mol Imaging. (2005) 32:1329–36. 10.1007/s00259-005-1912-y 16133377

[332] Klein N Sacher J Geiss-Granadia T Attarbaschi T Mossaheb N Lanzenberger R *In vivo* imaging of serotonin transporter occupancy by means of SPECT and [123I]ADAM in healthy subjects administered different doses of escitalopram or citalopram. Psychopharmacology. (2006) 188:263–72. 10.1007/s00213-006-0486-0 16955282

[333] Herold N Uebelhack K Franke L Amthauer H Luedemann L Bruhn H. Imaging of serotonin transporters and its blockade by citalopram in patients with major depression using a novel SPECT ligand [123I]-ADAM. J Neural Transm. (2006) 113:659–70. 10.1007/s00702-005-0429-7 16465456

[334] Meyer JH Wilson AA Ginovart N Goulding V Hussey D Hood K Occupancy of serotonin transporters by paroxetine and citalopram during treatment of depression: a [(11)C]DASB PET imaging study. Am J Psychiatry. (2001) 158:1843–9. 10.1176/appi.ajp.158.11.1843 11691690

[335] Parsey RV Kent JM Oquendo MA Richards MC Pratap M Cooper TB Acute occupancy of brain serotonin transporter by sertraline as measured by [11C]DASB and positron emission tomography. Biol Psychiatry. (2006) 59:821–8. 10.1016/j.biopsych.2005.08.010 16213473

[336] Voineskos AN Wilson AA Boovariwala A Sagrati S Houle S Rusjan P. Serotonin transporter occupancy of high-dose selective serotonin reuptake inhibitors during major depressive disorder measured with [11C]DASB positron emission tomography. Psychopharmacology. (2007) 193:539–45. 10.1007/s00213-007-0806-z 17497139

[337] Lundberg J Christophersen JS Petersen KB Loft H Halldin C Farde L. PET measurement of serotonin transporter occupancy: a comparison of escitalopram and citalopram. Int J Neuropsychopharmacol. (2007) 10:777–85. 10.1017/S1461145706007486 17201996

[338] Newberg AB Amsterdam JD Wintering N Ploessl K Swanson RL Shults J. 123I-ADAM binding to serotonin transporters in patients with major depression and healthy controls: a preliminary study. J Nucl Med Off Publ Soc Nucl Med. (2005) 46:973–7. 15937308

[339] Schuh-Hofer S Richter M Geworski L Villringer A Israel H Wenzel R. Increased serotonin transporter availability in the brainstem of migraineurs. J Neurol. (2007) 254:789–96. 10.1007/s00415-006-0444-0 17351723

[340] Bhagwagar Z Murthy N Selvaraj S Hinz R Taylor M Fancy S 5-HTT binding in recovered depressed patients and healthy volunteers: a positron emission tomography study with [11C]DASB. Am J Psychiatry. (2007) 164:1858–65. 10.1176/appi.ajp.2007.06111933 18056241

[341] Frankle WG Narendran R Huang Y Hwang D-R Lombardo I Cangiano C Serotonin transporter availability in patients with schizophrenia: a positron emission tomography imaging study with [11C]DASB. Biol Psychiatry. (2005) 57:1510–6. 10.1016/j.biopsych.2005.02.028 15953487

[342] Reimold M Smolka MN Zimmer A Batra A Knobel A Solbach C. Reduced availability of serotonin transporters in obsessive-compulsive disorder correlates with symptom severity – a [11C]DASB PET study. J Neural Transm. (2007) 114:1603–9. 10.1007/s00702-007-0785-6 17713719

[343] Brown AK George DT Fujita M Liow J-S Ichise M Hibbeln J. PET [11C]DASB imaging of serotonin transporters in patients with alcoholism. Alcohol Clin Exp Res. (2007) 31:28–32. 10.1111/j.1530-0277.2006.00261.x 17207098

[344] Cannon DM Ichise M Fromm SJ Nugent AC Rollis D Gandhi SK Serotonin transporter binding in bipolar disorder assessed using [11C]DASB and positron emission tomography. Biol Psychiatry. (2006) 60:207–17. 10.1016/j.biopsych.2006.05.005 16875929

[345] Kalbitzer J Frokjaer VG Erritzoe D Svarer C Cumming P Nielsen FÅ. The personality trait openness is related to cerebral 5-HTT levels. NeuroImage. (2009) 45:280–5. 10.1016/j.neuroimage.2008.12.001 19135154

[346] Kalbitzer J Erritzoe D Holst KK Nielsen FA Marner L Lehel S Seasonal changes in brain serotonin transporter binding in short serotonin transporter linked polymorphic region-allele carriers but not in long-allele homozygotes. Biol Psychiatry. (2010) 67:1033–9. 10.1016/j.biopsych.2009.11.027 20110086

[347] Huang Y-Y Huang W-S Ma K-H Chou T-K Kuo Y-Y Cheng C-Y Synthesis and comparison of 4-[18F]F-ADAM, 2-[18F]F-ADAM, N-Desmethyl-4-[18F]F-ADAM and [18F]F-AFM as serotonin transporter imaging agents. Appl Radiat Isot. (2012) 70:2298–307. 10.1016/j.apradiso.2012.06.005 22868170

[348] Yeh Y-W Ho P-S Kuo S-C Chen C-Y Liang C-S Yen C-H Disproportionate reduction of serotonin transporter may predict the response and adherence to antidepressants in patients with major depressive disorder: a positron emission tomography study with 4-[18F]-ADAM. Int J Neuropsychopharmacol. (2015) 18:pyu120 10.1093/ijnp/pyu120 25568284 PMC4540099

[349] Sullivan GM Oquendo MA Simpson N Van Heertum RL Mann JJ Parsey RV. Brain serotonin1A receptor binding in major depression is related to psychic and somatic anxiety. Biol Psychiatry. (2005) 58:947–54. 10.1016/j.biopsych.2005.05.006 16039621

[350] Santhosh L Estok KM Vogel RS Tamagnan GD Baldwin RM Mitsis EM. Regional distribution and behavioral correlates of 5-HT2A receptors in Alzheimer's disease with [18F]deuteroaltanserin and PET. Psychiatry Res Neuroimaging. (2009) 173:212–7. 10.1016/j.pscychresns.2009.03.007 19682865

[351] Bhagwagar Z Hinz R Taylor M Fancy S Cowen P Grasby P Increased 5-HT(2A) receptor binding in euthymic, medication-free patients recovered from depression: a positron emission study with [(11)C]MDL 100,907. Am J Psychiatry. (2006) 163:1580–7. 10.1176/ajp.2006.163.9.1580 16946184

[352] Perani D Garibotto V Gorini A Moresco RM Henin M Panzacchi A. *In vivo* PET study of 5HT(2A) serotonin and D(2) dopamine dysfunction in drug-naive obsessive-compulsive disorder. NeuroImage. (2008) 42:306–14. 10.1016/j.neuroimage.2008.04.233 18511303

[353] Saigal N Pichika R Easwaramoorthy B Collins D Christian BT Shi B. Synthesis and biologic evaluation of a novel serotonin 5-HT1A receptor radioligand, 18F-labeled mefway, in rodents and imaging by PET in a nonhuman primate. J Nucl Med Off Publ Soc Nucl Med. (2006) 47:1697–706. 17015907

[354] Wilson AA Ginovart N Hussey D Meyer J Houle S *In vitro* and *in vivo* characterisation of [11C]-DASB: a probe for *in vivo* measurements of the serotonin transporter by positron emission tomography. Nucl Med Biol. (2002) 29:509–15. 10.1016/S0969-8051(02)00316-5 12088720

[355] Selvaraj S Turkheimer F Rosso L Faulkner P Mouchlianitis E Roiser JP Measuring endogenous changes in serotonergic neurotransmission in humans: a [^11^C]CUMI-101 PET challenge study. Mol Psychiatry. (2012) 17:1254–60. 10.1038/mp.2012.78 22665264

[356] Elfving B Madsen J Knudsen GM. Neuroimaging of the serotonin reuptake site requires high-affinity ligands. Synapse. (2007) 61:882–8. 10.1002/syn.20443 17657807

[357] Maziere B Crouzel C Venet M Stulzaft O Sanz G Ottaviani M. Synthesis, affinity and specificity of 18F-setoperone, a potential ligand for *in-vivo* imaging of cortical serotonin receptors. Int J Rad Appl Instrum B. (1988) 15:463–8. 10.1016/0883-2897(88)90018-9 3255742

[358] Blin J Baron JC Dubois B Crouzel C Fiorelli M Attar-Lévy D Loss of brain 5-HT2 receptors in Alzheimer's disease. *In vivo* assessment with positron emission tomography and [18F]setoperone. Brain J Neurol. (1993) 116 (Pt 3):497–510. 10.1093/brain/116.3.497 8513389

[359] Chabriat H Tehindrazanarivelo A Vera P Samson Y Pappata S Boullais N. 5HT2 receptors in cerebral cortex of migraineurs studied using PET and 18F-fluorosetoperone. Cephalalgia Int J Headache. (1995) 15:104–8; discussion 77. 10.1046/j.1468-2982.1995.015002104.x 7641243

[360] Véra P Zilbovicius M Chabriat H Amarenco P Kerdraon J Ménard JF. Post-stroke changes in cortical 5-HT2 serotonergic receptors. J Nucl Med Off Publ Soc Nucl Med. (1996) 37:1976–81. 8970517

[361] Massou JM Trichard C Attar-Levy D Feline A Corruble E Beaufils B. Frontal 5-HT2A receptors studied in depressive patients during chronic treatment by selective serotonin reuptake inhibitors. Psychopharmacology. (1997) 133:99–101. 10.1007/s002130050377 9335087

[362] Shiue GG Choi S-R Fang P Hou C Acton PD Cardi C N,N-Dimethyl-2-(2-Amino-4-18F-Fluorophenylthio)-Benzylamine (4-18F-ADAM): an improved PET radioligand for serotonin transporters. J Nucl Med. (2003) 44:1890–7. 14660713

[363] Shah M Seibyl J Cartier A Bhatt R Catafau AM. Molecular imaging insights into neurodegeneration: focus on α-synuclein radiotracers. J Nucl Med Off Publ Soc Nucl Med. (2014) 55:1397–400. 10.2967/jnumed.113.136515 25091474

[364] Burré J Sharma M Tsetsenis T Buchman V Etherton MR Südhof TC. Alpha-synuclein promotes SNARE-complex assembly *in vivo* and *in vitro*. Science. (2010) 329:1663–7. 10.1126/science.1195227 20798282 PMC3235365

[365] El-Agnaf OMA Salem SA Paleologou KE Cooper LJ Fullwood NJ Gibson MJ. Alpha-synuclein implicated in Parkinson's disease is present in extracellular biological fluids, including human plasma. FASEB J Off Publ Fed Am Soc Exp Biol. (2003) 17:1945–7. 10.1096/fj.03-0098fje 14519670

[366] Lee H-J Patel S Lee S-J. Intravesicular localization and exocytosis of alpha-synuclein and its aggregates. J Neurosci Off J Soc Neurosci. (2005) 25:6016–24. 10.1523/JNEUROSCI.0692-05.2005 15976091 PMC6724798

[367] Dickson DW Braak H Duda JE Duyckaerts C Gasser T Halliday GM. Neuropathological assessment of Parkinson's disease: refining the diagnostic criteria. Lancet Neurol. (2009) 8:1150–7. 10.1016/S1474-4422(09)70238-8 19909913

[368] Brooks DJ Tambasco N. Imaging synucleinopathies. Mov Disord Off J Mov Disord Soc. (2016) 31:814–29. 10.1002/mds.26547 26879635

[369] Paleologou KE Kragh CL Mann DMA Salem SA Al-Shami R Allsop D. Detection of elevated levels of soluble alpha-synuclein oligomers in post-mortem brain extracts from patients with dementia with Lewy bodies. Brain J Neurol. (2009) 132(Pt 4):1093–101. 10.1093/brain/awn349 19155272

[370] Choi JH Stubblefield B Cookson MR Goldin E Velayati A Tayebi N. Aggregation of α-synuclein in brain samples from subjects with glucocerebrosidase mutations. Mol Genet Metab. (2011) 104:185–8. 10.1016/j.ymgme.2011.06.008 21742527 PMC3352315

[371] Kotzbauer PT Cairns NJ Campbell MC Willis AW Racette BA Tabbal SD. Pathologic accumulation of α-synuclein and Aβ in parkinson disease patients with dementia. Arch Neurol. (2012) 69:1326–31. 10.1001/archneurol.2012.1608 22825369 PMC3616136

[372] Schildknecht S Gerding HR Karreman C Drescher M Lashuel HA Outeiro TF. Oxidative and nitrative alpha-synuclein modifications and proteostatic stress: implications for disease mechanisms and interventions in synucleinopathies. J Neurochem. (2013) 125:491–511. 10.1111/jnc.12226 23452040

[373] Uchihara T Nakamura A Mochizuki Y Hayashi M Orimo S Isozaki E. Silver stainings distinguish Lewy bodies and glial cytoplasmic inclusions: comparison between Gallyas-Braak and Campbell-Switzer methods. Acta Neuropathol. (2005) 110:255–60. 10.1007/s00401-005-1044-2 16003542

[374] Anderson JP Walker DE Goldstein JM de Laat R Banducci K Caccavello RJ. Phosphorylation of Ser-129 is the dominant pathological modification of alpha-synuclein in familial and sporadic Lewy body disease. J Biol Chem. (2006) 281:29739–52. 10.1074/jbc.M600933200 16847063

[375] Maetzler W Reimold M Liepelt I Solbach C Leyhe T Schweitzer K. [11C]PIB binding in Parkinson's disease dementia. NeuroImage. (2008) 39:1027–33. 10.1016/j.neuroimage.2007.09.072 18035558

[376] Fodero-Tavoletti MT Mulligan RS Okamura N Furumoto S Rowe CC Kudo Y. *In vitro* characterisation of BF227 binding to alpha-synuclein/Lewy bodies. Eur J Pharmacol. (2009) 617:54–8. 10.1016/j.ejphar.2009.06.042 19576880

[377] Kikuchi A Takeda A Okamura N Tashiro M Hasegawa T Furumoto S. *In vivo* visualization of alpha-synuclein deposition by carbon-11-labelled 2-[2-(2-dimethylaminothiazol-5-yl)ethenyl]-6-[2-(fluoro)ethoxy]benzoxazole positron emission tomography in multiple system atrophy. Brain J Neurol. (2010) 133(Pt 6):1772–8. 10.1093/brain/awq091 20430832

[378] Verdurand M Levigoureux E Lancelot S Zeinyeh W Billard T Quadrio I Amyloid-beta radiotracer [18F]BF-227 does not bind to cytoplasmic glial inclusions of postmortem multiple system atrophy brain tissue. Contrast Media Mol Imaging. (2018) 2018:9165458 10.1155/2018/9165458 29551958 PMC5818909

[379] Bagchi DP Yu L Perlmutter JS Xu J Mach RH Tu Z. Binding of the radioligand SIL23 to α-synuclein fibrils in Parkinson disease brain tissue establishes feasibility and screening approaches for developing a Parkinson disease imaging agent. PLoS ONE. (2013) 8:e55031. 10.1371/journal.pone.0055031 23405108 PMC3566091

[380] Verdurand M Levigoureux E Zeinyeh W Berthier L Mendjel-Herda M Cadarossanesaib F. *In silico, in vitro*, and *in vivo* evaluation of new candidates for α-synuclein PET imaging. Mol Pharm. (2018) 15:3153–66. 10.1021/acs.molpharmaceut.8b00229 29989823

[381] Ye L Velasco A Fraser G Beach TG Sue L Osredkar T *In vitro* high affinity alpha-synuclein binding sites for the amyloid imaging agent PIB are not matched by binding to Lewy bodies in postmortem human brain. J Neurochem. (2008) 105:1428–37. 10.1111/j.1471-4159.2008.05245.x 18221373 PMC2408655

[382] Fodero-Tavoletti MT Smith DP McLean CA Adlard PA Barnham KJ Foster LE. *In vitro* characterization of pittsburgh compound-B binding to lewy bodies. J Neurosci. (2007) 27:10365–71. 10.1523/JNEUROSCI.0630-07.2007 17898208 PMC6673163

[383] McKeith IG. Consensus guidelines for the clinical and pathologic diagnosis of dementia with Lewy bodies (DLB): report of the Consortium on DLB International Workshop. J Alzheimers Dis JAD. (2006) 9(Suppl. 3):417–23. 10.3233/JAD-2006-9S347 16914880

[384] Gilman S Low PA Quinn N Albanese A Ben-Shlomo Y Fowler CJ. Consensus statement on the diagnosis of multiple system atrophy. J Neurol Sci. (1999) 163:94–8. 10.1016/S0022-510X(98)00304-9 10223419

[385] Deutsch SI Rosse RB Schwartz BL Mastropaolo J. A revised excitotoxic hypothesis of schizophrenia: therapeutic implications. Clin Neuropharmacol. (2001) 24:43. 10.1097/00002826-200101000-00008 11290881

